# Neuroprotective Effect of Remote Ischemic Conditioning on Patients Undergoing Intravenous Thrombolysis: A Randomized Controlled Trial

**DOI:** 10.1002/mco2.70696

**Published:** 2026-03-18

**Authors:** Shuang Qi, Yang Qu, Jia Liu, Kangjia Song, Yucen Ma, Yao‐De He, Peng Zhang, Yi Gao, Yuli Fu, Pan‐Deng Zhang, Yi Yang, Zhen‐Ni Guo

**Affiliations:** ^1^ Stroke Center, Department of Neurology The First Hospital of Jilin University Chang Chun China; ^2^ Laboratory For Engineering and Scientific Computing, Institute of Advanced Computing and Digital Engineering, Shenzhen Institute of Advanced Technology Chinese Academy of Sciences Shenzhen China; ^3^ Neuroscience Research Centre The First Hospital of Jilin University Chang Chun China

**Keywords:** brain injury biomarker, cerebral autoregulation, intravenous thrombolysis, neuron‐specific enolase, remote ischemic conditioning

## Abstract

Intravenous thrombolysis (IVT) is the treatment with the highest level of evidence for acute ischemic stroke, but about half of patients fail to achieve a favorable prognosis. This study (NCT05598658) proposed a treatment strategy of adjunctive two sessions of remote ischemic conditioning (RIC) within 24 h after IVT, and evaluated the effects through cerebral autoregulation (CA) and brain‐injury biomarkers. Patients were randomized (1:1) to the RIC or sham‐RIC groups, which received 200 and 60 mmHg RIC, respectively, at 6 and 18–24 h after IVT. CA was assessed at 2 and 7 days after IVT and serum brain‐injury biomarkers were evaluated at 24 h after IVT. The primary outcome was CA at 2 days after IVT. A total of 100 patients were randomized to the RIC or sham‐RIC group. Ipsilateral CA was significantly higher in the RIC group than in the sham‐RIC group at 2 days (β: 14.970 [95% confidence interval, 7.741–22.199; *p* < 0.001]) and 7 days after IVT. Simultaneously, neuron‐specific enolase level at 24 h after IVT was significantly lower in the RIC than the sham‐RIC group. These results suggest that adjunctive two sessions of RIC within 24 h after IVT can effectively exert neuroprotective effects in patients with IVT.

## Graphical abstract

Adjunctive remote ischemic conditioning (RIC) administered twice within 24 h after intravenous thrombolysis (IVT) improved cerebral autoregulation, suggesting that RIC exerted neuroprotective effects in patients undergoing IVT.

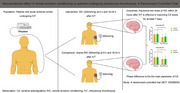

## 1 Introduction

Stroke remains a major cause of morbidity and mortality worldwide [1], with ischemic stroke accounting for approximately 62.4% of all stroke cases [2]. Intravenous thrombolysis (IVT) is the treatment with the highest level of evidence for efficacy in the acute phase of ischemic stroke [3], but only approximately half of the patients achieve favorable outcomes [4]. Therefore, it is necessary to explore new IVT combination therapies to improve the proportion of favorable outcomes in patients undergoing IVT.

Remote ischemic conditioning (RIC), which refers to brief transient episodes of ischemia and reperfusion applied to distant tissues or organs (often the arms and legs), renders remote tissues and organs resistant to subsequent prolonged ischemic insult [5]. Animal studies have shown that RIC can reduce infarct volume [6] and alleviate damage in the blood–brain barrier [7, 8, 9], and a large‐scale clinical trial has demonstrated its safety and efficacy in patients with moderate acute ischemic stroke [10]. However, results are inconsistent with regard to RIC being beneficial when IVT is applied [11, 12, 13].

Recent advances in stroke research have fundamentally reshaped the understanding of ischemic stroke as a systemic disease rather than a purely focal cerebral event. Acute cerebral ischemia triggers extensive bidirectional cross‐talk between the central nervous system and peripheral organs, including the heart, lungs, liver, kidneys, spleen, and immune system, through neural, humoral, and immunological pathways [14, 15]. This complex brain–body interaction plays a critical role in shaping vascular function, inflammatory responses, blood–brain barrier stability, and secondary neuronal injury after stroke. From this systems biology perspective, therapeutic strategies capable of modulating systemic responses may exert neuroprotection beyond the ischemic lesion itself. Notably, RIC is thought to induce a systemic protective phenotype by activating endogenous defense mechanisms across multiple organs, making it particularly well suited for targeting this central nervous system–systemic organ cross‐talk.

In this study, we proposed a treatment strategy of adjunctive two sessions of RIC within 24 h after IVT, because the use of antiplatelet agents within 24 h of IVT is limited, and there is no recommended neuroprotective treatment strategy. This study focused on changes in cerebrovascular function and levels of biomarkers of brain injury to evaluate the effect of this combination therapy strategy on patients undergoing IVT.

Cerebral autoregulation (CA) refers to the mechanism of maintaining stable brain perfusion and is an important indicator of cerebrovascular function [16, 17, 18]. This dynamic process ensures continuous oxygen and nutrient delivery to meet the metabolic demands of the brain tissue, safeguarding neuronal function and integrity. Therefore, it is important to explore effective methods to improve CA in patients undergoing IVT. Our previous study found that RIC significantly improved CA in healthy adults [19]. In this study, we explored whether RIC could improve CA in patients undergoing IVT. In addition, we investigated the effects of RIC on three classic biomarkers of brain injury, ubiquitin C‐terminal hydrolase‐L1 (UCH‐L1), neuron‐specific enolase (NSE), and S100β, which reflect neuronal and astroglial damage.

We used a randomized controlled trial design to assess the neuroprotective effects of RIC in terms of protecting CA and reducing the extent of brain damage. We compared CA at 2 and 7 days after IVT following two rounds of RIC or sham‐RIC at 6 and 18–24 h after IVT. Additionally, we measured the serum levels of the three biomarkers (UCH‐L1, NSE, and S100β) before and 24 h after IVT.

## 2 Results

### 2.1 Participant Characteristics

**FIGURE 1:**
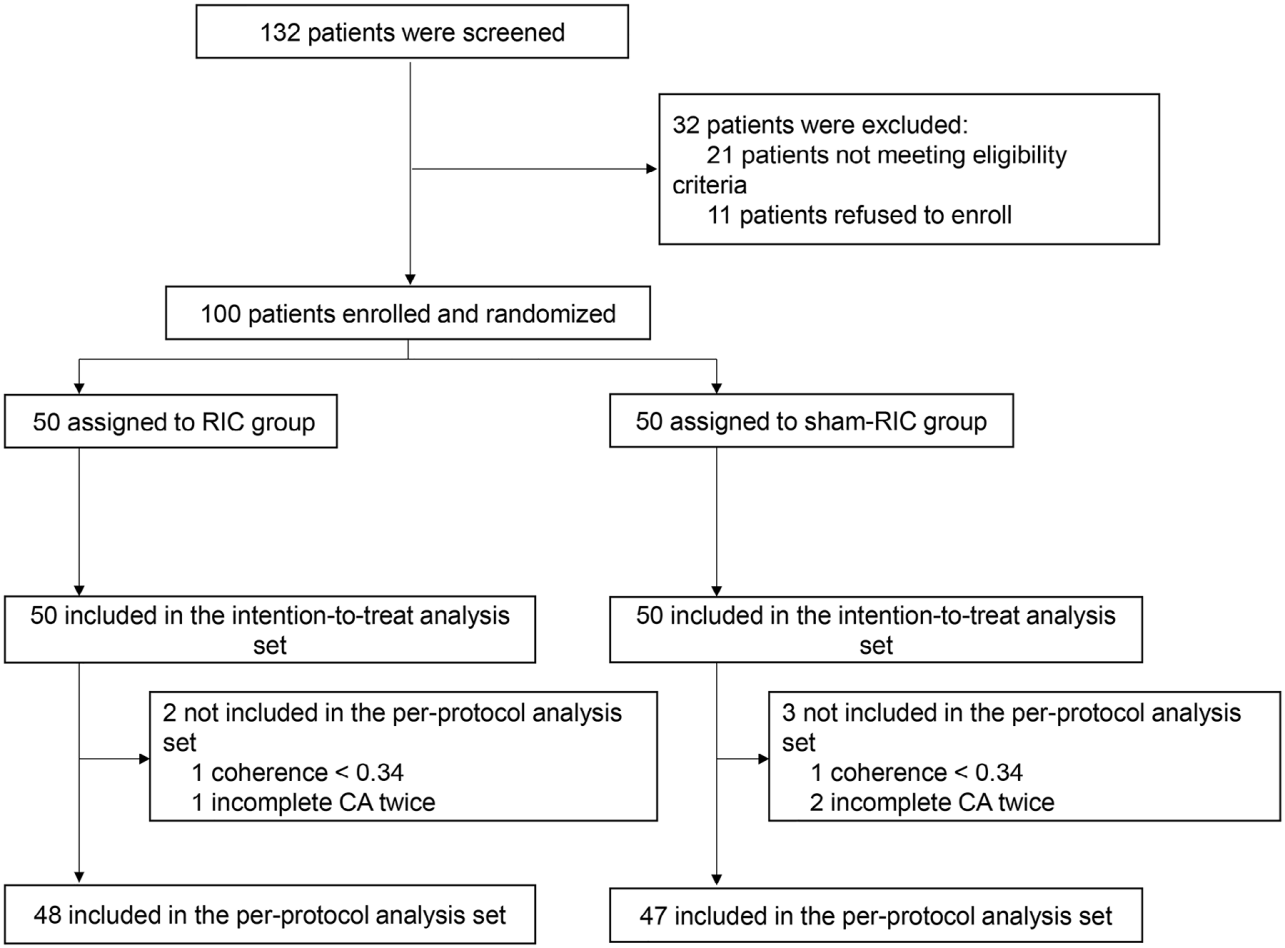
Flowchart of the study. RIC, remote ischemic conditioning; CA, cerebral autoregulation.

The study initially enrolled 100 patients (50 each in the RIC and sham‐RIC groups), who were included in the intention‐to‐treat (ITT) analysis set. Two patients with coherence <0.34 and three patients who did not complete CA monitoring twice were excluded. Finally, 48 patients in the RIC group and 47 in the sham‐RIC group were included in the per‐protocol (PP) analysis set (Figure 1). There were no significant differences in demographic information, vascular risk factors, laboratory test results, or clinical characteristics between RIC and sham‐RIC group patients in the ITT and PP sets (Table 1).

**TABLE 1:** Baseline demographic and clinical characteristics of patients.

	Per‐protocol analysis set (*N* = 95)			Intention‐to‐treat analysis set (*N* = 100)		
Characteristic	RIC group (*n* = 48)	Sham‐RIC group (*n* = 47)	*p*	RIC group (*n* = 50)	Sham‐RIC group (*n* = 50)	*p*
Age (years)	58.19 ± 9.52	59.57 ± 11.58	0.525	58.10 ± 9.37	59.50 ± 11.46	0.505
Sex (male, %)	40 (83.3)	38 (80.9)	0.752	42 (84.0)	41 (82.0)	0.790
Vascular risk factors, *n* (%)						
Hypertension	28 (58.3)	27 (57.4)	0.930	29 (58.0)	30 (60.0)	0.839
Diabetes mellitus	15 (31.3)	13 (27.7)	0.701	15 (30.0)	13 (26.0)	0.656
Smoking	24 (50.0)	30 (63.8)	0.174	26 (52.0)	33 (66.0)	0.155
Drinking	28 (58.3)	25 (53.2)	0.614	30 (60.0)	26 (52.0)	0.420
CHD	5 (10.4)	3 (6.4)	0.714^*^	5 (10.0)	3 (6.0)	0.175^*^
Hematologic indices						
PNR	34.83 (24.90–51.45)	38.60 (28.79–49.31)	0.671	34.83 (25.49–51.59)	38.50 (26.89–49.31)	0.940
HCY (µmol/L)	12.62 (10.46–18.54)	13.73 (10.80–16.20)	0.818	12.62 (10.95–19.83)	13.85 (10.82–16.71)	0.994
hs‐CRP (mg/L)	3.13 (2.03–3.60)	3.23 (2.07–7.07)	0.275	3.13 (2.09–3.53)	3.18 (2.06–6.01)	0.327
LDL‐C (mmol/L)	3.23 ± 0.70	3.33 ± 0.75	0.486	3.22 ± 0.69	3.35 ± 0.75	0.372
Hemoglobin A1c (%)	5.70 (5.40–6.28)	6.00 (5.50–7.20)	0.196	5.70 (5.40–6.23)	5.90 (5.50–6.90)	0.239
Pre‐thrombolysis NIHSS score	7.00 (5.25–11.00)	7.00 (6.00–10.00)	0.904	7.50 (5.75–11.00)	7.00 (6.00–10.00)	0.738
SBP on admission (mmHg)	155.85 ± 23.41	159.49 ± 23.87	0.456	156.20 ± 23.76	159.72 ± 23.40	0.457
DBP on admission (mmHg)	90.52 ± 15.07	92.36 ± 13.75	0.536	90.60 ± 15.33	92.54 ± 13.36	0.502
HR on admission (beats/min)	72.00 (67.00–82.00)	72.00 (65.00–88.00)	0.549	72.00 (67.00–83.75)	72.00 (64.50–88.00)	0.583
TOAST			0.881			0.883
LAA	10 (20.8)	11 (23.8)		10 (20.0)	12 (24.0)	
SVO	30 (62.5)	27 (57.4)		31 (62.0)	29 (58.0)	
UE	8 (16.7)	9 (19.1)		9 (18.0)	9 (18.0)	
End‐tidal CO_2_ at 2 days^a^	41.50 (38.25–43.75)	40.00 (37.00–42.00)	0.064	41.50 (38.00–43.25)	40.00 (37.00–42.00)	0.076
End‐tidal CO_2_ at 7 days^b^	40.00 (36.00–43.00)	40.00 (38.00–42.00)	0.489	40.00 (36.00–43.00)	40.00 (38.00–42.00)	0.414
Coherence at 2 days^c^						
Affected	0.67 (0.50–0.81)	0.70 (0.52–085)	0.671	0.67 (0.51–0.82)	0.70 (0.51–0.84)	0.845
Unaffected	0.70 (0.58–0.85)	0.69 (0.54–0.86)	0.929	0.71 (0.59–0.86)	0.69 (0.55–0.85)	0.769
Coherence at 7 days^d^						
Affected	0.68 (0.54–0.79)	0.71 (0.51–0.81)	0.532	0.68 (0.54–0.79)	0.71 (0.52–0.81)	0.433
Unaffected	0.68 (0.54–0.78)	0.69 (0.50–0.84)	0.470	0.68 (0.54–0.78)	0.70 (0.52–0.84)	0.375
Pre‐thrombolysis brain‐injury biomarker^e^						
UCH‐L1 (pg/mL)	86.65 (60.85–208.04)	94.35 (30.90–172.10)	0.536	86.65 (58.48–212.37)	94.04 (32.31–171.71)	0.503
NSE (ng/mL)	9.57 (7.37–15.35)	12.75 (8.32–16.01)	0.408	9.57 (7.19–15.10)	12.64 (8.34–15.89)	0.333
S100β (ng/mL)	0.02 (0.01–0.05)	0.04 (0.00–0.10)	0.200	0.02 (0.01–0.05)	0.03 (0.00–0.09)	0.165

Abbreviations: CHD, coronary artery heart disease; DBP, diastolic blood pressure; HCY, homocysteine; HR, heart rate; hs‐CRP, high‐sensitivity C‐reactive protein; LAA, large artery atherosclerosis; LDL‐C, low‐density lipoprotein; NIHSS, National Institutes of Health Stroke Scale; PNR, platelet‐to‐neutrophil ratio; RIC, remote ischemic conditioning; SBP, systolic blood pressure; SVO, small artery occlusion; TOAST, trial of org 10 172 in acute stroke treatment; UE, stroke of undetermined etiology.

* Fisher exact test.

^a^ Data on end‐tidal CO_2_ at 2 days were available for 99 patients in Intention‐to‐treat analysis set (RIC group, *n* = 50; sham‐RIC group, *n* = 49).

^b^ Data on end‐tidal CO_2_ at 7 days were available for 96 patients in Intention‐to‐treat analysis set (RIC group, *n* = 48; sham‐RIC group, *n* = 48).

^c^ Data on coherence at 2 days were available for 99 patients in Intention‐to‐treat analysis set (RIC group, *n* = 50; sham‐RIC group, *n* = 49).

^d^ Data on coherence at 7 days were available for 96 patients in Intention‐to‐treat analysis set (RIC group, *n* = 48; sham‐RIC group, *n* = 48).

^e^ Data on pre‐thrombolysis brain‐injury biomarker were available for 72 patients in Intention‐to‐treat analysis set (RIC group, *n* = 36; sham‐RIC group, *n* = 36) and 69 patients in per‐protocol analysis set (RIC group, *n* = 34; sham‐RIC group, *n* = 35).

### 2.2 Primary Outcome

**FIGURE 2:**
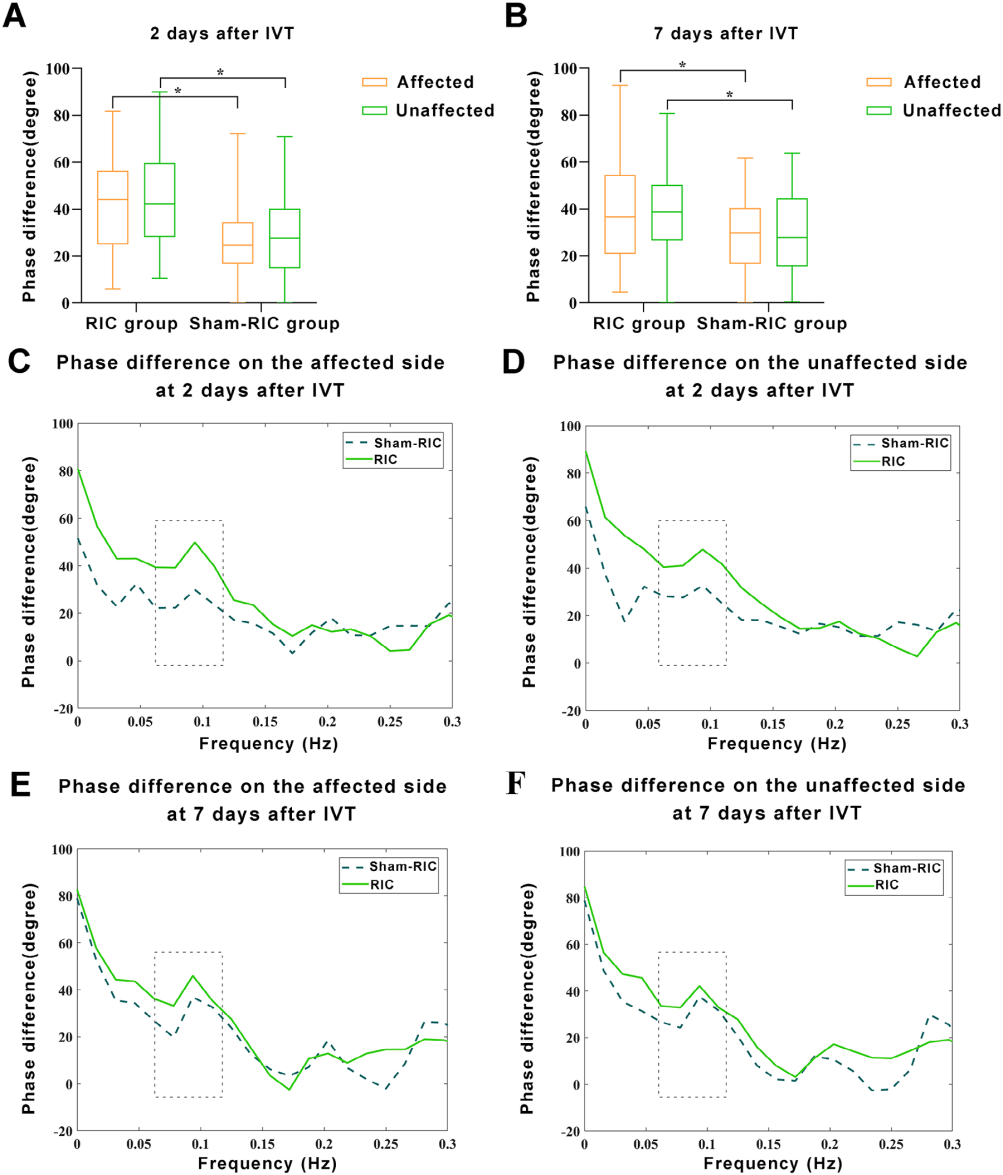
Comparison of PD between the RIC and sham‐RIC groups at 2 and 7 days after IVT in the intention‐to‐treat analysis set. Box plots show the statistical distribution of PD at low frequency (0.06–0.12 Hz) for both groups (A and B). PD values are displayed across the frequency domain on both affected and unaffected sides (C–F). *p< 0.001 in PD on both sides at 2 days after IVT; *p* = 0.009 in PD on the unaffected side at 7 days after IVT; *p* = 0.008 in PD on the affected side at 7 days after IVT. RIC, remote ischemic conditioning; PD, phase difference; IVT, intravenous thrombolysis.

In the ITT analysis set, phase difference (PD) was significantly higher on the affected side of the RIC group than in the sham‐RIC group at 2 days after IVT (Figure 2A). The adjusted beta coefficient was 14.970 (95% confidence interval [CI], 7.741–22.199; *p* < 0.001) (Table 2). Figure 2C shows the PD in the frequency domain on the affected side at 2 days after IVT. In the PP analysis set, the PD result on the affected side at 2 days after IVT was similar to that of the ITT analysis set (Table 3, Figure S1).

**TABLE 2:** The association between remote ischemic conditioning and outcomes in intention‐to‐treat analysis set.

Variability	Measure of effect	Unadjusted value (95% CI)	*p*	Adjusted value^a^ (95% CI)	*p*
Primary outcomes					
Cerebral autoregulation					
2 days after IVT					
Phase difference, degree					
Affected	Beta coefficient	15.453 (8.220–22.687)	< 0.001	14.970 (7.741–22.199)	< 0.001
Secondary outcomes					
Cerebral autoregulation					
2 days after IVT					
Phase difference, degree					
Unaffected	Beta coefficient	13.694 (6.276–21.113)	< 0.001	13.433 (6.077–20.789)	< 0.001
Gain, %/mmHg					
Unaffected	Beta coefficient	0.002 (−0.187 to 0.192)	0.981	0.001 (−0.190 to 0.192)	0.993
Affected	Beta coefficient	−0.021 (−0.206 to 0.163)	0.820	−0.023 (−0.210 to 0.164)	0.808
7 days after IVT					
Phase difference, degree					
Unaffected	Beta coefficient	10.517 (3.209–17.824)	0.005	9.874 (2.638–17.109)	0.007
Affected	Beta coefficient	10.665 (2.860–18.470)	0.007	9.905 (2.353–17.457)	0.010
Gain, %/mmHg					
Unaffected	Beta coefficient	0.013 (−0.205 to 0.231)	0.908	0.007 (−0.211 to 0.224)	0.953
Affected	Beta coefficient	0.023 (−0.161 to 0.207)	0.807	0.021 (−0.164 to 0.207)	0.821
NIHSS at 24 h	Beta coefficient	−0.540 (−1.949 to 0.869)	0.452	−0.726 (−2.018 to 0.566)	0.271
NIHSS at 7 days	Beta coefficient	−0.280 (−1.498 to 0.938)	0.652	−0.352 (−1.582 to 0.878)	0.575
Favorable outcome (mRS 0–2)	Odds ratio	1.000 (0.375–2.664)	> 0.999	0.955 (0.349–2.608)	0.928
Hemorrhagic transformation	Odds ratio	0.235 (0.025–2.178)	0.202	0.075 (0.003–1.792)	0.110

Abbreviations: IVT, intravenous thrombolysis; mRS, modified Rankin Scale; NIHSS, National Institutes of Health Stroke Scale; RIC, remote ischemic conditioning.

^a^ Adjusted for age, sex, and pre‐thrombolysis NIHSS score.

**TABLE 3:** The association between remote ischemic conditioning and outcomes in per‐protocol analysis set.

Variability	RIC group (*n* = 48)	Sham‐RIC group (*n* = 47)	Measure of effect	Unadjusted value (95% CI)	*p*	Adjusted value^a^ (95% CI)	*p*
Primary outcomes							
Cerebral autoregulation							
2 days after IVT							
Phase difference, degree							
Affected	41.77 ± 19.79	26.79 ± 17.05	Beta coefficient	14.977 (7.444–22.511)	< 0.001	14.508 (6.959–22.056)	< 0.001
Secondary outcomes							
Cerebral autoregulation							
2 days after IVT							
Phase difference, degree							
Unaffected	43.56 ± 19.67	30.49 ± 18.39	Beta coefficient	13.064 (5.302–20.826)	0.001	12.905 (5.209–20.602)	0.001
Gain, %/mmHg							
Unaffected	0.99 (0.78–1.20)	0.88 (0.67–1.23)	Beta coefficient	0.034 (−0.159 to 0.228)	0.724	0.031 (−0.165 to 0.227)	0.758
Affected	0.85 (0.69–1.24)	0.75 (0.62–1.23)	Beta coefficient	0.013 (−0.176 to 0.203)	0.888	0.009 (−0.184 to 0.202)	0.926
7 days after IVT							
Phase difference, degree							
Unaffected	39.55 ± 19.25	29.47 ± 117.81	Beta coefficient	10.083 (2.522–17.644)	0.010	9.486 (1.983–16.989)	0.014
Affected	40.26 ± 21.95	29.56 ± 17.15	Beta coefficient	10.690 (2.654–18.727)	0.010	9.927 (2.174–17.680)	0.013
Gain, %/mmHg							
Unaffected	1.17 (0.79–1.42)	1.00 (0.70–1.52)	Beta coefficient	0.035 (−0.185 to 0.255)	0.753	0.029 (−0.191 to 0.248)	0.797
Affected	1.03 (0.75–1.41)	0.99 (0.73–1.30)	Beta coefficient	0.035 (−0.154 to 0.223)	0.716	0.031 (−0.159 to 0.221)	0.746
Brain‐injury biomarkers^b^							
UCH‐L1 (pg/mL)	156.66 (106.89–271.83)	158.46 (83.06‐231.03)	Beta coefficient	−1.118 (−73.387 to 71.150)	0.975	4.604 (−68.867 to 78.075)	0.900
NSE (ng/mL)	10.80 (8.66–14.22)	15.12 (9.68‐22.65)	Beta coefficient	−6.613 (−12.392 to −0.835)	0.026	−8.410 (−14.182 to −2.637)	0.005
S100β (ng/mL)	0.03 (0.01–0.07)	0.02 (0.00‐0.06)	Beta coefficient	−0.024 (−0.112 to 0.065)	0.592	−0.036 (−0.127 to 0.055)	0.430
NIHSS at 24 h	4.00 (2.00–7.00)	4.00 (2.00‐7.00)	Beta coefficient	−0.458 (−1.940 to 1.025)	0.541	−0.602 (−1.956 to 0.752)	0.379
NIHSS at 7 days	2.50 (1.00–5.75)	4.00 (1.00‐5.00)	Beta coefficient	−0.309 (−1.583 to 0.964)	0.631	−0.360 (−1.650 to 0.930)	0.581
Favorable outcome (mRS 0–2)	38 (79.2)	37 (78.7)	Odds ratio	0.974 (0.363–2.611)	0.958	0.932 (0.338–2.567)	0.892
Hemorrhagic transformation	1 (2.1)	3 (6.4)	Odds ratio	0.312 (0.031–3.113)	0.321	0.186 (0.007–4.679)	0.307
Infarct volume^c^ (mL)	2.42 (0.59–8.31)	4.69 (0.85‐13.45)	Beta coefficient	−3.176 (−13.263 to 6.910)	0.533	−3.288 (−13.316 to 6.739)	0.516

Abbreviations: IVT, intravenous thrombolysis; mRS, modified Rankin Scale; NIHSS, National Institutes of Health Stroke Scale; NSE, neuron‐specific enolase; RIC, remote ischemic conditioning; UCH‐L1, ubiquitin C‐terminal hydrolase‐L1.

^a^ Adjusted for age, sex, and pre‐thrombolysis NIHSS score.

^b^ Data on brain‐injury biomarkers were available for 49 patients (RIC group, *n* = 24; sham‐RIC group, *n* = 25).

^c^ Data on infarct volume were available for 92 patients (RIC group, *n* = 46; sham‐RIC group, *n* = 46).

### 2.3 Secondary Outcome

In the ITT analysis set, PD was significantly higher on the unaffected side in the RIC than in the sham‐RIC group at 2 days after IVT (Figure 2A). The adjusted beta coefficient was 13.433 (95% CI, 6.077–20.789; *p* < 0.001) (Table 2). Figure 2D shows the PD in the frequency domain on the unaffected side at 2 days after IVT. The PD result on the unaffected side at 2 days after IVT in the PP analysis set was similar to that in the ITT analysis set (Table 3, Figure S1).

In the ITT analysis set, PD were significantly higher on the affected and unaffected sides in the RIC group than in the sham‐RIC group at 7 days after IVT (Figure 2B). The adjusted beta coefficients for PD on the affected and unaffected sides at 7 days after IVT were 9.905 (95% CI, 2.353–17.457; *p* = 0.010) and 9.874 (95% CI, 2.638–17.109; *p* = 0.007), respectively (Table 2). Figure 2E,F shows the PD in the frequency domain on both sides at 7 days after IVT. The PD results on the affected and unaffected sides at 7 days after IVT in the PP analysis set were similar to those in the ITT analysis set (Table 3, Figure S1).

For the parameter of gain, no difference was found between the two groups on either the affected or unaffected side at both 2 and 7 days, and the results were similar in the ITT and PP data sets.

The results of CA parameters at very low frequency (VLF, 0.02–0.07 Hz), low frequency (LF, 0.07–0.20 Hz), and high frequency (HF, 0.20–0.50 Hz) are shown in Table S1. PD at 2 days after IVT on the affected side was significantly higher in the RIC group than in the sham‐RIC group at VLF and LF frequencies, while no significant difference was found in the unaffected side of PD at this time point. For PD at HF measured at 2 days after IVT, and both PD and gain measured at 7 days, no significant increased effect of RIC was observed.

**FIGURE 3:**
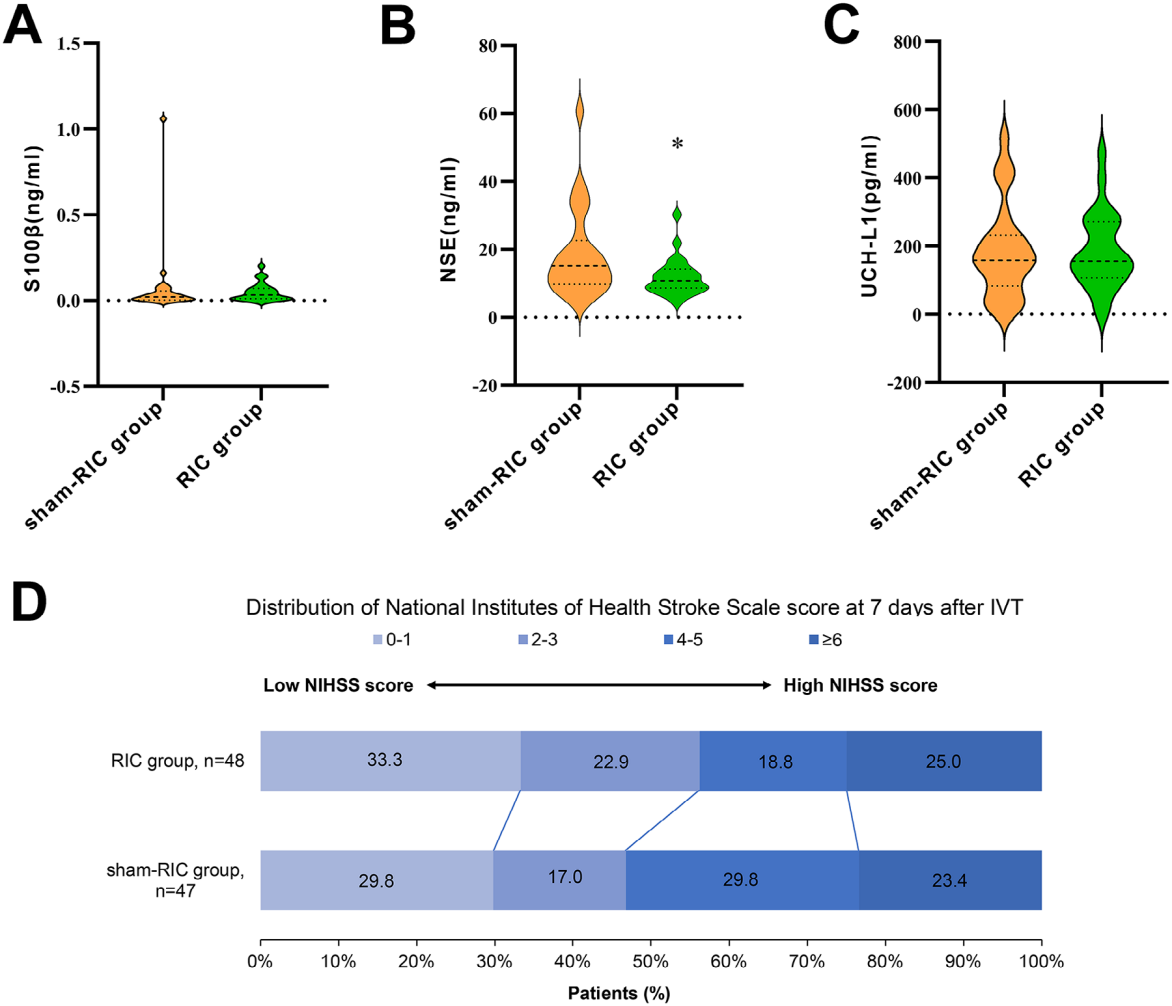
Levels of serum biomarkers at 24 h after IVT and distribution of stroke severity scores at 7 days after IVT. Ubiquitin C‐terminal hydrolase‐L1, NSE, and S100β in the RIC group and sham‐RIC group at 24 h after IVT (A–C) and the distribution of National Institutes of Health Stroke Scale in the RIC group and sham‐RIC group at 7 days after IVT in the per‐protocol analysis set (D). *p = 0.049 in NSE at 24 h after IVT. RIC, remote ischemic conditioning; NSE, neuron‐specific enolase; IVT, intravenous thrombolysis.

There were no significant differences in the National Institutes of Health Stroke Scale (NIHSS) scores at 24 h and 7 days, proportion of patients with favorable outcomes at 90 days, incidence of hemorrhagic transformation, levels of brain‐injury markers (UCH‐L1 and S100β) at 24 h, or infarct volume between the groups in both the ITT analysis set and the PP analysis set (Tables 2 and 3). In contrast, in the PP analysis set, serum NSE levels were lower in the RIC group than in the sham‐RIC group at 24 h after IVT, and the adjusted beta coefficient was −8.410 (95% CI, −14.182 to −2.637; *p* = 0.005, Table 3 and Figure 3A–C). Figure 3D shows the distribution of the NIHSS scores in the PP analysis set at 7 days after IVT. Although no statistical difference was observed, the proportion of an NIHSS score of 0–3 at 7 days after IVT in the RIC group was higher than that in the sham‐RIC group (56.2% vs. 46.8%).

### 2.4 Safety Outcome

A total of 100 patients were enrolled in the safety analysis set, and one patient in the RIC group died at 3 months of follow‐up throughout the study. No other definite adverse events occurred.

## 3 Discussion

Our findings reveal that a treatment strategy of adjunctive two sessions of RIC within 24 h after IVT is effective in improving CA and reducing serum NSE levels in patients undergoing IVT, which suggests that RIC plays a neuroprotective role in these patients.

CA maintains cerebral blood flow within a relatively constant range during fluctuations in blood pressure [20]. It has been demonstrated that CA is impaired in the ultra‐early phase of acute ischemic stroke and is closely related to the prognosis of acute ischemic stroke [21]. Therefore, CA is an important target for improvement in patients with acute ischemic stroke, but currently, limited methods to improve CA are available. We have previously found that RIC can improve CA in healthy adults, but whether RIC can improve CA in patients with acute ischemic stroke, especially patients who receive IVT, remains unclear [19]. In the present study, we proposed a treatment strategy involving adjunctive two sessions of RIC within 24 h after IVT, and we observed significant improvements, with markedly higher CA levels sustained over a relatively long period in the RIC group after two sessions of RIC. By enhancing this regulatory mechanism, RIC may help maintain adequate cerebral perfusion, which is essential for minimizing ischemic damage and improving patient outcomes.

Various mechanisms potentially underlie the RIC‐mediated enhancement of CA; we think that the improvement of CA by RIC is likely attributable to its protective effects on the neurovascular unit through suppression of inflammatory responses and oxidative stress. Both oxidative stress and inflammation are key pathological processes associated with cerebral ischemia/reperfusion injury [22]. Previous animal studies have demonstrated that RIC may activate the Nrf2/HO‐1 pathway to mitigate oxidative stress and inflammatory responses, thereby ameliorating neurobehavioral outcomes [23]. Other potential mechanisms may be that RIC alleviates hypoxia signals, enhances tissue oxygen exchange, improves oxygen supply to brain tissue, and reduces apoptosis after ischemia‐reperfusion [24]. Further research is needed to unambiguously reveal the specific mechanism.

We also examined the effects of RIC on three serum brain‐injury biomarkers and found that serum NSE levels were lower in the RIC group than in the sham‐RIC group. NSE is a specific marker for neuronal injury, and elevated levels in serum are indicative of neuronal damage. The significant reduction in NSE levels found in the RIC group in the present study intuitively illustrates the protective effect of RIC on neurons in patients who undergo IVT. After an acute ischemic stroke, the blood–brain barrier breaks down, and nerve cells become ischemic and hypoxic, leading to the release of certain substances into the bloodstream [25]. Previous studies have shown that RIC can partially prevent blood–brain barrier destruction aggravated by recombinant tissue plasminogen activator (rt‐PA) after ischemic stroke [7], which may explain the decrease in serum NSE levels observed in this study.

We did not observe the effects of RIC on clinical outcomes in this study. This is consistent with the results of the SERIC‐IVT study, which administered RIC twice daily for 7 days to patients undergoing IVT, but did not observe that RIC improved the proportion of excellent outcomes [26]. The following are several possible reasons for the inconsistency between CA and NSE and clinical outcomes. First, stroke prognosis is inherently determined by a complex interplay of multiple factors, including infarct location and volume, collateral circulation, reperfusion quality, systemic inflammation, comorbidities, and post‐stroke care. Improvements in CA and NSE, although statistically significant, may represent only partial modulation of this multifactorial process and may be insufficient on their own to produce detectable changes in global functional outcomes. Second, the observed changes in CA and NSE reflect early vascular stabilization and attenuation of neuronal injury, which constitute important biological signals of neuroprotection. However, such intermediate endpoints do not necessarily translate into long‐term functional improvement. Third, the sample size of this study was relatively small. Finally, the included patients had mild‐to‐moderate stroke severity, and their relatively favorable natural prognosis may have produced a ceiling effect in these patients.

In this study, we specifically focused on patients undergoing IVT. Given that hemodynamic alterations in patients receiving bridging therapy may differ from those of patients undergoing IVT alone, potentially influencing the effects of RIC, we excluded patients who underwent endovascular therapy. In addition, we excluded patients with atrial fibrillation, as it remains a significant cause of acute ischemic stroke and can lead to hemodynamic disturbances in these patients. Moreover, we observed that the proportion of patients with IVT caused by small artery occlusion was higher than that of other Trial of Org 10172 in Acute Stroke Treatment classifications, about 60%. One possible reason is that all included patients had mild‐to‐moderate symptoms. The median pre‐thrombolytic NIHSS score of our patients was 7, indicating that more than half of the patients had mild‐to‐moderate stroke and that small‐vessel disease was more common in these patients.

Our study has several limitations. First, we excluded patients with atrial fibrillation and those receiving bridging therapy to reduce hemodynamic and etiologic heterogeneity; although these criteria enhanced internal validity, they inevitably limit the generalizability of our findings to the broader population of patients treated with IVT in clinical practice. Second, our sample size was relatively small, which may underlie the lack of improvement in 90‐day modified Rankin Scale (mRS) scores after RIC in patients undergoing IVT. Third, the enrolled patients predominantly had small infarct volumes and mild‐to‐moderate stroke severity at baseline (median NIHSS 7, with approximately 60% classified as small‐vessel occlusion). The inherently good natural prognosis of these mild strokes may create a ceiling effect that may have considerably limited our ability to detect additional functional benefits from the intervention at 90 days. Fourth, although serum brain‐injury biomarkers were analyzed in a single batch using the same assay platform, reagent lot, and calibration protocol, potential assay variability and pre‐analytical factors (including sample handling and storage) cannot be entirely excluded, which may have contributed to variability in biomarker measurements.

## 4 Conclusions

This study confirms that adjunctive two sessions of RIC within 24 h after IVT are effective in improving CA levels for at least 7 days and also reducing serum NSE levels in patients undergoing IVT, suggesting a neuroprotective role in patients with IVT.

## 5 Materials and Methods

### 5.1 Study Design

The Effect of Remote Ischemic Conditioning on Cerebral Hemodynamics in Patients After Intravenous Thrombolysis (RICCH‐IVT) was a single‐center, single‐blinded, endpoint‐blinded, randomized controlled clinical trial, registered at ClinicalTrials.gov (NCT 05598658). The study was approved by the Ethics Committee of the First Hospital of Jilin University (23K028). Written informed consent was obtained from all participants. All procedures performed in studies involving human participants were in accordance with the 1964 Helsinki declaration and its later amendments or comparable ethical standards. The participants had the right to withdraw from the study at any point.

### 5.2 Participants

Patients with acute ischemic stroke who received IVT at the First Hospital of Jilin University between April 2023 and January 2024 were enrolled in this study, and the date of final follow‐up was April 2024. The inclusion criteria were as follows: (1) age ≥ 18 and < 80 years, both sexes; (2) a clear clinical diagnosis of acute ischemic stroke and treatment with standard rt‐PA (0.9 mg/kg) IVT within 4.5 h of stroke onset; (3) pre‐onset mRS score ≤ 1; (4) baseline NIHSS score ≥ 5 and ≤ 25; and (5) Glasgow Coma Scale score ≥ 8. The exclusion criteria were as follows: (1) having received bridging therapy (IVT plus mechanical thrombectomy); (2) previous history of atrial fibrillation or electrocardiographic evidence of atrial fibrillation; (3) contraindications to RIC treatment or previous RIC treatment or similar treatment; (4) pregnancy or breastfeeding; (5) life expectancy of ≤ 3 months or inability to complete the study for other reasons; (6) unwillingness to be followed up or poor treatment compliance or participation in other clinical studies; and (7) insufficient bilateral temporal bone windows for insonation of the middle cerebral artery.

### 5.3 Randomization and Blinding

Randomization was established by a researcher who was not involved in the recruitment and assessment of the participants. A random numerical sequence with a 1:1 allocation ratio was computer generated by an independent biostatistician using SPSS (IBM Corp., Armonk, NY, USA) before starting the study. The randomization code was concealed using sequentially numbered sealed opaque envelopes. After the baseline assessment and obtaining written informed consent, a researcher who was not involved in the data analysis or clinical ratings opened the sealed envelope to identify the group to which the participant was allocated (RIC or sham‐RIC group) and performed the treatment.

To maintain strict participant blinding, multiple complementary measures were implemented. First, the automated RIC device used identical pre‐programmed modes that produced the same acoustic signals, cuff inflation/deflation rhythms, cycle duration, total procedure time, and visual cues. The selection buttons had no indicator lights or markings after being pressed, and the devices were identical in appearance, color, weight, and sound. Second, the treating investigator who selected the mode and initiated the procedure was strictly prohibited from discussing any aspect of pressure settings or intervention details with patients, bedside nurses, or outcome assessors. Third, patients enrolled during the same period were placed in different wards and managed by the same medical team. Participants were unaware of their assigned groups.

### 5.4 Procedures

Once written informed consent was obtained, the patients were randomly assigned to the RIC or sham RIC group. The patients in the RIC and sham‐RIC groups received RIC at 200 and 60 mmHg, respectively, 6 and 18–24 h after IVT. Patients in both groups underwent CA at 2 and 7 days after IVT. Blood samples were drawn from the cubital vein of the patients both immediately before IVT and again at 24 h after IVT, and stored at −80°C in the Biobank Department of Jilin University's First Hospital Clinical Research Division after centrifugation. These blood samples were analyzed in a single batch by the same technician using the same reagent lot and the same calibration curve once all patients had completed enrollment. Magnetic resonance diffusion‐weighted imaging was conducted 3–7 days after IVT to measure the infarct volume.

All patients received standard medical treatment according to the guidelines for the management of acute ischemic stroke [27]. Other data, including demographic information, vascular risk factors, laboratory test results, clinical characteristics, and follow‐up data, were collected.

### 5.5 Outcomes

The primary endpoint was PD (the main parameter of CA) on the affected side at 2 days after IVT. The secondary outcomes included PD on the unaffected side at 2 days after IVT, gain (the parameter of CA) on the affected and unaffected sides at 2 and 7 days after IVT, PD on the affected and unaffected sides at 7 days after IVT, NIHSS score at 24 h and 7 days after IVT, proportion of patients with favorable outcomes at 90 days, final infarct volume, incidence of hemorrhagic transformation within 24 h, and serum levels of brain‐injury biomarkers at 24 h after IVT. Safety outcomes included mortality and all adverse events within 90 days. A favorable outcome was defined as an mRS score of ≤ 2 at 90 days. Hemorrhagic transformation was determined according to the European‐Australasian Acute Stroke Study classification criteria 24 h after IVT [28]. The outcome observers were blinded to group information.

### 5.6 Sample Size

We estimated that PD on the affected side at 2 days after IVT would be 30.12° in the sham‐RIC group, according to our pre‐experimental work. Here, we estimated a 9° improvement with RIC based on the results of preliminary observations. Using a two‐tailed *t*‐test to compare the difference between means and assuming α = 0.05 and statistical power of 80%, the estimated sample size was 80 patients (40 each in the RIC and sham‐RIC groups). Considering a 20% loss to follow up, we estimated that 100 patients would be required (50 each in the RIC and sham‐RIC groups).

### 5.7 RIC Procedure

RIC was performed using an automatic medical device (BB‐RIC‐D5/LAPUL Medical Devices Co., Ltd., Beijing, China). In the RIC group, one round of RIC was composed of four cycles of 5 min of ischemia (at 200 mmHg) followed by 5 min of reperfusion on the unilateral upper limb of the unaffected side. In the sham‐RIC group, the patients received RIC at 60 mmHg; the rest of the operation was the same as that of the RIC group. For the enrolled patients, when a venous indwelling needle was required for intravenous treatment or blood sampling, it was routinely placed in the affected side arm, which was not used for RIC. This was done to ensure the safety of both the infusion and the RIC treatment. Similar to previous studies, 60 mmHg was selected as the control pressure mainly to exclude interfering factors of ineffective pressure and ensure reliability of the study results. Importantly, 60 mmHg is generally considered insufficient to trigger effective ischemic conditioning effects [29, 30].

The device has four pre‐programmed modes (M1–M4). Before the start of the trial, M3 mode was permanently configured for RIC (200 mmHg, 5 min inflation/5 min deflation × 4 cycles, total duration 35 min), and M4 mode was permanently configured for sham‐RIC (60 mmHg, same cycle timing and duration). After randomization, the treating investigator—who was not involved in patient assessment or outcome evaluation—selected only the corresponding mode (M3 for RIC group, M4 for sham‐RIC group) by pressing the power button followed by the M3 or M4 button. The device then operated fully automatically and stopped after exactly 35 min.

### 5.8 CA Assessment and Analysis

In both groups, CA was monitored twice, at 2 and 7 days after IVT, and evaluated as previously reported [19]. Before the examination, all patients were instructed to relax in the supine position for 10 min in a dedicated quiet examination room with a controlled temperature ranging from 20°C to 24°C. The bilateral cerebral blood flow velocity (CBFV) in the middle cerebral artery was measured using transcranial Doppler (MultiDop X4; DWL, Sipplingen, Germany) sonography. Two 2‐MHz probes were fixed with a customized head frame in the bilateral temporal bone window at a depth of 45–60 mm. Simultaneously, arterial blood pressure in the digital artery (measured using a servo‐controlled plethysmograph [Finometer Model 1; FMS, Amsterdam, the Netherlands]) was continuously recorded. The end‐tidal CO_2_ was measured using a capnograph (MultiDop X4; DWL) with a nasal cannula. The real‐time recordings lasted 10 min and were stored for further analysis.

All CA data were processed via transfer function analysis using MATLAB (MathWorks, Natick, MA, USA). PD, gain, and coherence function within a low‐frequency range (0.06–0.12 Hz) were generated as the main observation indexes in this study. PD at VLF (0.02–0.07 Hz), LF (0.07–0.20 Hz), and HF (0.20–0.50 Hz) were also calculated in our study. PD reflected the delay of CBFV following changes in arterial blood pressure. PD is widely regarded as the most robust, reproducible, and clinically relevant index of dynamic CA by transcranial doppler, as it directly quantifies the temporal buffering capacity of the cerebral vasculature against blood pressure oscillations [31]. Gain reflected the transfer of the amplitude of arterial blood pressure to CBFV. Therefore, a low PD and high gain indicated impaired CA. The coherence function was used to test the linearity of the CBFV and arterial blood pressure values. A coherence value < 0.34 indicates excessive noise or non‐linear/non‐stationary signal behavior, rendering transfer function estimates (phase, gain) unreliable [32]. Only data with coherence ≥ 0.34 (number of windows: 5; critical values of coherence: 5%) were included in subsequent statistical analyses [32].

### 5.9 Serum Brain‐Injury Biomarker Testing

The levels of UCH‐L1, NSE, and S100β were detected using the MS‐Fast/Aceso 80A automated magnetic particle‐based chemiluminescence enzyme immunoassay system developed by Sophonix. Biotinylated capture antibodies are linked to various proteins and alkaline phosphatase‐labeled detection antibodies in a sandwich manner and react with excess magnetic particles of streptomycin avidin layers to form a complex. In a magnetic field, complexes aggregate, and sensitivity is thus increased. The coefficient of variation was < 8.0%.

### 5.10 Infarct Volume Measurement

Infarct volume was determined using magnetic resonance diffusion‐weighted imaging, and the lesion contour was plotted for each individual to calculate the area, using the Philips IntelliSpace Portal imaging tool (Philips, Eindhoven, the Netherlands). The thickness of each layer was multiplied by the infarct area and added to obtain the infarct volume [33]. Professionals blinded to the clinical data and randomization performed these measurements.

### 5.11 Statistical Analyses

SPSS 27.0 was used for the statistical analyses. The Shapiro–Wilk test was used to test the distribution of continuous variables. Normally distributed continuous variables are described as the mean ± standard deviation, and Student's *t*‐test was used to compare differences. Non‐normally distributed continuous variables are presented as medians (quartiles), and differences were compared using the Mann–Whitney *U* test. Categorical variables were described as frequencies and percentages, and examined using the chi‐square test. Both the ITT analysis set and the data of the PP analysis set were used to analyze the effect of RIC on the primary and secondary outcomes. Multiple imputations, based on five replications, were used to impute missing values in the ITT analysis set. Unadjusted and adjusted associations between RIC and outcomes were estimated using linear or logistic regression models, as appropriate. Multivariable models were prespecified in the statistical analysis plan and adjusted for the following baseline covariates: age, sex, and pre‐thrombolysis NIHSS score. Because of significant missing data, the infarct volume and serum levels of brain‐injury biomarkers were only analyzed in the PP analysis set. In addition, to observe the effect of RIC on short‐term prognosis, we divided the RIC and sham‐RIC groups into four subgroups based on the NIHSS quartile of all patients at 7 days after IVT in the PP analysis set, and showed the distribution. A two‐tailed *p*‐value < 0.05 was considered statistically significant.

## Author Contributions

S.Q. and Y.Q. contributed to drafting the text and figures. S.Q., K.S., Y.M., Y.H., Y.G., and Y.F. conducted the experiments; S.Q., Y.Q., K.S., and P.Z. contributed analysis methods. S.Q., Y.Q., J.L., K.S., Y.M., Y.H., P.Z., Y.G., Y.F., and P.Z. contributed to data acquisition and analyses. J.L. and P.Z. provided technical advice. S.Q., Y.Q., Y.Y., and Z.G. contributed to study concept and design. Z.G. and Y.Y. review the text and provide fundings. All authors read and approved the final manuscript.

## Funding

This project was supported by the National Natural Science Foundation of China (U24A20686), the Norman Bethune Health Science Center of Jilin University (2022JBGS03), Science and Technology Department of Jilin Province (YDZJ202302CXJD061, 20220303002SF), the Jilin Provincial Key Laboratory (YDZJ202302CXJD017) to Yi Yang and the Talent Reserve Program of the First Hospital of Jilin University (JDYYCB‐2023002) to Zhen‐Ni Guo.

## Ethics Statement

The study was approved by the Ethics Committee of the First Hospital of Jilin University (23K028). Written informed consent was obtained from all participants.

## Conflicts of Interest

The authors declare no conflicts of interest.

## Supporting information




**Table S1**: The association between remote ischemic conditioning and cerebral autoregulation at very low frequency (0.02–0.07 Hz), low frequency (0.07–0.20 Hz) and high frequency (0.20–0.50 Hz) in intention‐to‐treat analysis set
**Figure S1**: Comparison of PD between the RIC and sham‐RIC groups at 2 and 7 days after IVT in per‐protocol analysis set.

## Data Availability

The datasets generated and analyzed during the current study are available from the corresponding author on reasonable request.
