# Supplementary material for: Neuroprotective Effect of Remote Ischemic Conditioning on Patients Undergoing Intravenous Thrombolysis: A Randomized Controlled Trial
Source: MedComm (2020). 2026 Mar 18;7(4):e70696. doi: 10.1002/mco2.70696 (PMC13042378; doi:10.1002/mco2.70696)
Supplement: Supplementary file 1 — Table S1: The association between remote ischemic conditioning and cerebral autoregulation at very low frequency (0.02–0.07 Hz), low frequency (0.07–0.20 Hz) and high frequency (0.20–0.50 Hz) in intention‐to‐treat analysis set Figure S1: Comparison of PD between the RIC and sham‐RIC groups at 2 and 7 days after IVT in per‐protocol analysis set. [file MCO2-7-e70696-s001.pdf]

Neuroprotective effect of remote ischemic conditioning on patients undergoing intravenous thrombolysis: A randomized controlled trial

Running title: Neuroprotective effect of RIC

Author (s):

Shuang Qi, MD[1]; Yang Qu, MD[1]; Jia Liu, PhD.[3]; Kangjia Song, MD[1]; Yucen Ma, MD[1]; Yao-De He, MD[1]; Peng Zhang, MD[1], Yi Gao, MD[1], Yuli Fu, MD[1]; Pan-Deng Zhang, PhD[3]; Yi Yang, MD, PhD[1,2]*; Zhen-Ni Guo, MD, PhD[1,2]*

Equal Author Contributions:

Shuang Qi and Yang Qu contributed equally to the manuscript. Yi Yang and Zhen-Ni Guo are corresponding authors of the manuscript.

Correspondence Author:

Yi Yang, MD, PhD, Stroke Center, Department of Neurology, the First Hospital of Jilin University, Chang Chun, China; Neuroscience Research Centre, the First Hospital of Jilin University, Chang Chun, China (yang_yi@jlu.edu.cn).

Zhen-Ni Guo, MD, PhD, Department of Neurology, the First Hospital of Jilin University, Chang Chun, China; Neuroscience Research Centre, the First Hospital of Jilin University, Chang Chun, China (zhen1ni2@jlu.edu.cn)

Affiliations: 1. Stroke Center, Department of Neurology, the First Hospital of Jilin University, Chang Chun, China; 2. Neuroscience Research Centre, the First Hospital of Jilin University, Chang Chun, China; 3. Laboratory for Engineering and Scientific Computing, Institute of Advanced Computing and Digital Engineering, Shenzhen Institute of Advanced Technology, Chinese Academy of Sciences, Shenzhen, China.

Table S1: The association between remote ischemic conditioning and cerebral autoregulation at very low frequency (0.02–0.07 Hz), low frequency (0.07–0.20 Hz) and high frequency (0.20–0.50 Hz) in intention-to-treat analysis set.

Figure S1: Comparison of PD between the RIC and sham-RIC groups at 2 and 7 days after IVT in per-protocol analysis set. Data were available for 95 patients at 2 and 7 days (RIC group, n=48; sham-RIC group, n=47). Box plots show the statistical distribution of PD at low frequency (0.06–0.12 Hz) for both groups (A-B). PD values are displayed across the frequency domain on both affected and unaffected sides (C-F). *:P<0.05. RIC, remote ischemic conditioning; PD, phase difference; IVT, intravenous thrombolysis.

Supplementary Materials

Supplemental Table S1: The association between remote ischemic conditioning and cerebral autoregulation at very low frequency (0.02–0.07 Hz), low frequency (0.07–0.20 Hz) and high frequency (0.20–0.50 Hz) in intention-to-treat analysis set 3

Supplemental Figure S1: Comparison of PD between the RIC and sham-RIC groups at 2 and 7 days after IVT in per-protocol analysis set 5

RICCH-IVT Study Protocols 6

Final Protocol (Issue Date: March 05, 2023) 6

Original Protocol (Issue Date: December 26, 2022) 28

Summary of change 50

Statistical Analysis Plan 57

Statistical Analysis Plan Version 2.0 57

Statistical Analysis Plan Version 1.0 69

Details of Statistical Analysis Plan Change 81

This supplemental material was provided by the authors to provide readers with additional information on their work.

Table S1 The association between remote ischemic conditioning and cerebral autoregulation at very low frequency (0.02–0.07 Hz), low frequency (0.07–0.20 Hz) and high frequency (0.20–0.50 Hz) in intention-to-treat analysis set

Variability	Measure of effect	Unadjusted value (95% CI)	P	Adjusted value[£] (95% CI)	p
**2 days after IVT**					
**Phase difference at VLF, degree**					
Unaffected	Beta coefficient	12.326 (0.202-24.451)	0.046	11.558 (-0.625-23.740)	0.063
Affected	Beta coefficient	13.477 (1.921-25.033)	0.022	12.468 (1.103-23.834)	0.032
**Gain at VLF, %/mmHg**					
Unaffected	Beta coefficient	0.041 (-0.120-0.202)	0.619	0.042 (-0.122-0.205)	0.616
Affected	Beta coefficient	0.026 (-0.124-0.177)	0.731	0.029 (-0.122-0.181)	0.705
**Phase difference at LF, degree**					
Unaffected	Beta coefficient	5.287 (-1.467-12.040)	0.125	4.961 (-1.670-11.591)	0.143
Affected	Beta coefficient	8.233 (1.436-15.030)	0.018	7.920 (1.244-14.597)	0.020
**Gain at LF, %/mmHg**					
Unaffected	Beta coefficient	0.139 (-0.072-0.351)	0.196	0.143 (-0.070-0.355)	0.187
Affected	Beta coefficient	0.149 (-0.048-0.346)	0.138	0.151 (-0.047-0.350)	0.136
**Phase difference at HF, degree**					
Unaffected	Beta coefficient	-4.985 (-10.755-0.785)	0.090	-4.968 (-10.810-0.874)	0.096
Affected	Beta coefficient	-4.350 (-9.681-0.981)	0.110	-4.257 (-9.636-1.122)	0.121
**Gain at HF, %/mmHg**					
Unaffected	Beta coefficient	0.018 (-0.185-0.220)	0.863	0.024 (-0.179-0.228)	0.816
Affected	Beta coefficient	0.064 (-0.124-0.253)	0.504	0.070 (-0.118-0.259)	0.463
**7 days after IVT**					
**Phase difference at VLF, degree**					
Unaffected	Beta coefficient	8.590 (-3.303-20.483)	0.157	9.522 (-2.161-21.206)	0.110
Affected	Beta coefficient	4.656 (-7.185-16.497)	0.441	4.970 (-6.666-16.606)	0.403
**Gain at VLF, %/mmHg**					
Unaffected	Beta coefficient	0.059 (-0.083-0.202)	0.414	0.055 (-0.090-0.201)	0.455
Affected	Beta coefficient	0.006 (-0.123-0.134)	0.931	0.008 (-0.123-0.139)	0.906
**Phase difference at LF, degree**					
Unaffected	Beta coefficient	6.553 (-0.587-13.693)	0.072	6.387 (-0.756-13.531)	0.080
Affected	Beta coefficient	5.121 (-1.979-12.222)	0.157	4.946 (-2.055-11.947)	0.166
**Gain at LF, %/mmHg**					
Unaffected	Beta coefficient	0.049 (-0.160-0.258)	0.645	0.043 (-0.167-0.253)	0.689
Affected	Beta coefficient	0.082 (-0.098-0.261)	0.373	0.078 (-0.104-0.260)	0.400

**Phase difference at HF, degree**					
Unaffected	Beta coefficient	1.559 (-4.178-7.836)	0.626	1.461 (-4.765-7.688)	0.645
Affected	Beta coefficient	2.573 (-3.874-9.020)	0.434	2.670 (-3.601-8.941)	0.404
**Gain at HF, %/mmHg**					
Unaffected	Beta coefficient	0.158 (-0.075-0.392)	0.184	0.153 (-0.084-0.389)	0.205
Affected	Beta coefficient	0.171 (-0.049-0.391)	0.125	0.170 (-0.049-0.390)	0.128

Note:

£: Adjusted for group, age, sex, and baseline NIHSS score.

VLF: 0.02–0.07 Hz, LF: 0.07–0.20 Hz, HF: 0.20–0.50 Hz.

Abbreviation:

RIC: remote ischemic conditioning; CA: cerebral autoregulation; VLF: very low frequency; LF: low frequency; HF: high frequency; IVT: intravenous thrombolysis.

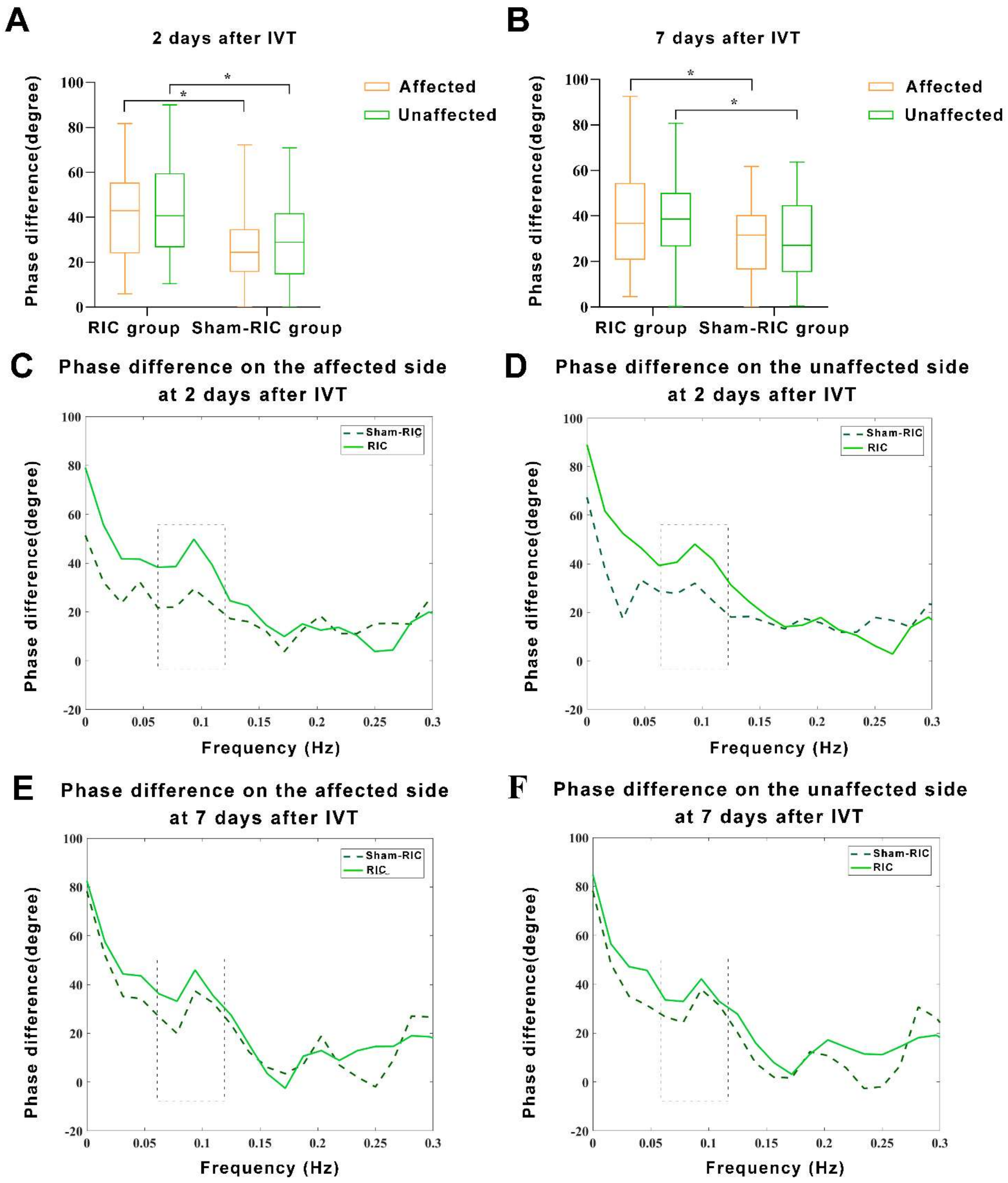

Figure S1: Comparison of PD between the RIC and sham-RIC groups at 2 and 7 days after IVT in per-protocol analysis set. Data were available for 95 patients at 2 and 7 days (RIC group, n=48; sham-RIC group, n=47). Box plots show the statistical distribution of PD at low frequency (0.06–0.12 Hz) for both groups (A-B). PD values are displayed across the frequency domain on both affected and unaffected sides (C-F). *:P<0.05. RIC, remote ischemic conditioning; PD, phase difference; IVT, intravenous thrombolysis.

RICCH-IVT Study Protocols

Effect of Remote Ischemic Conditioning on Cerebral Hemodynamics in Patients After Intravenous Thrombolysis

(RICCH-IVT)

Final protocol (Version 2.0, 05 March 2023)

Contents

1. Study Synopsis **9**

2. List of Abbreviations **12**

3. Background Information **14**

4. Study Objectives and Outcomes **15**

4.1 Study Objectives 15

4.2 Study Outcomes 15

4.3 Defination of Outcome Parameter 15

5. Sample Size **16**

6. Trial Design **16**

7. Randomization **16**

8. The Flow of the Trial **16**

8.1 Visit 1: Screening and Baseline Stage (-4.5-6 h) 16

8.2 Visit 2: Treatment Stage (6-24 h) 17

8.3 Visit 3: Follow-up Stage (1-7 d) 18

8.4 Visit 4: Follow-up Stage (90±3 d) 18

9. Combination Medications **18**

10. Patient Population **19**

10.1 Inclusion Criteria 19

10.2 Exclusion Criteria 19

10.3 Suspension Criteria 19

10.4 Dropout Criteria 19

11. Adverse Events **19**

11.1 Definition of Adverse Events 20

11.2 Observation, Assessment, Recording, and Reporting of Adverse Events 20

11.3 Severity Grading Criteria for Adverse Events 20

11.4 Association between Adverse Events and Study Treatment 20

11.5 Management of Adverse Events 21

11.6 Serious Adverse Events 21

12. Statistical Analysis **22**

13. Appendix **23**

13.1 Appendix1: Study Flow Chart 23

13.2 Appendix 2: Modified Rankin Scale 24

13.3 Appendix 3: National Institutes of Health Stroke Scale..25
14. Reference 27

1. Study Synopsis

**Study Tiltle**	Effect of Remote Ischemic Conditioning on Cerebral Hemodynamics in Patients After Intravenous Thrombolysis (RICCH-IVT)
**Principal Research Center**	The First Hospital of Jilin University
**Principal Investigator**	Pro. Yi Yang, M.D, Ph.D. Stroke Center, Department of Neurology, the First Hospital of Jilin University, Chang Chun, China
**Objective**	To determine the effect of remote ischemic conditioning on cerebral hemodynamics in patients after intravenous thrombolysis.
**Trial Design**	This study aims to enroll 100 consecutive patients with acute ischemic stroke who receive intravenous thrombolytic (IVT) therapy. The enrolment follows the principles of randomization and parallel control. The patients will be randomly assigned to remote ischemic conditioning (RIC) group or sham-RIC group, maintaining a 1:1 ratio for a study period of 90 days.<br>Both groups will receive cerebral autoregulation (CA) assessments at 2 and 7 days after IVT. The RIC group will undergo RIC at 6 and 18–24 h after IVT, involving pressurization of the unaffected arm with a cuff at a threshold of 200 mmHg (at the same position as the blood pressure measuring cuff) for 5 min, followed by reperfusion for 5 min. This process will be repeated for four cycles, totaling 40 min.<br>Patients in the sham-RIC group will undergo sham RIC at 6 and 18–24 h after IVT, which includes pressurization of the unaffected arm with a cuff at 60 mmHg (at the same position as the blood pressure measuring cuff) for 5 min, followed by reperfusion for 5 min. This will be repeated for four cycles, totaling 40 min.<br>Follow-up will continue until 90 days after IVT in both groups to determine the effect of RIC on cerebral hemodynamics in patients after IVT.
**Outcomes**	**Primary Efficacy Outcome:**<br>The primary endpoint is phase difference (PD, the main parameter of CA) on the affected side at 2 days after IVT.<br><br>**Secondary Efficacy Outcomes:**<br>1) PD on the unaffected side at 2 days after IVT.<br>2) Gain (the parameter of CA) on the affected side at 2 days after IVT.<br>3) Gain on the unaffected side at 2 days after IVT.<br>4) PD on the affected side at 7 days after IVT.

	5) PD on the unaffected side at 7 days after IVT.<br>6) Gain on the affected side at 7 days after IVT.<br>7) Gain on the unaffected side at 7 days after IVT.<br>8) NIHSS score at 24 h and 7 days after IVT.<br>9) Proportion of patients with favorable outcomes (mRS score≤ 2) at 90 days.<br>10) Final infarct volume.<br>11) Incidence of hemorrhagic transformation within 24 hours.<br>12) Serum levels of brain-injury biomarkers at 24 h after IVT.<br><br>**Safety Outcomes:**<br>1) Mortality within 90 days.<br>2) All adverse events within 90 days.
**Inclusion Criteria**	1) Age ≥ 18 and < 80 years, both sexes.<br>2) A clear clinical diagnosis of acute ischemic stroke and treatment with standard rt-PA (0.9 mg/kg) IVT within 4.5 h of stroke onset.<br>3) Pre-onset modified Rankin Scale (mRS) score ≤1.<br>4) Baseline National Institute of Health Stroke Scale (NIHSS) score ≥5 and ≤25.<br>5) Glasgow Coma Scale score ≥8.
**Exclusion Criteria**	1) Having received bridging therapy (IVT plus mechanical thrombectomy).<br>2) Previous history of atrial fibrillation or electrocardiographic evidence of atrial fibrillation.<br>3) Contraindications to RIC treatment or previous RIC treatment or similar treatment.<br>4) Pregnancy or breastfeeding.<br>5) Life expectancy of ≤3 months or inability to complete the study for other reasons.<br>6) Unwillingness to be followed up or poor treatment compliance or participation in other clinical studies.<br>7) Had insufficient bilateral temporal bone windows for insonation of the middle cerebral artery.
**Interventions**	All subjects will receive standard clinically routine treatments for acute ischemic stroke and other underlying diseases.<br><br>**RIC group:** RIC (200 mmHg) combined with alteplase IVT group<br>1) Method: external use, cuff fixed on the unaffected upper arm (same position as the blood pressure measuring cuff).<br>2) Treatment pressure: 200 mmHg.<br>3) Treatment mode: 5 min of pressurization followed by 5 min of rest as a cycle, repeated for four cycles, with a total duration

	of 40 min.<br>4) Treatment cycle: 6 and 18–24 h after IVT.<br><br>**Sham-RIC group:** Sham RIC (60 mmHg) combined with alteplase IVT group<br>1) Method: external use, cuff fixed on the unaffected upper arm (same position as the blood pressure measuring cuff).<br>2) Treatment pressure: 60 mmHg.<br>3) Treatment mode: 5 min of pressurization, followed by 5 min of rest as a cycle, repeated for four cycles, with a total duration of 40 min.<br>4) Treatment cycle: 6 and 18–24 h after IVT.
**Follow-up**	Follow-up will continue until 90 days after IVT in both groups.
**Sample Size**	100

2. List of Abbreviations

**Abbreviations**	**Full title**
AIS	Acute Ischemic Stroke
AE	Adverse Event
CRF	Case Report Form
CT	Computed Tomography
DWI	Diffusion Weighted Imaging
ICH	Intracranial Hemorrhage
IVT	Intravenous Thrombolysis
mRS	modified Rankin Scale
MRI	Magnetic Resonance Imaging
NIHSS	National Institute of Health Stroke Scale
NRS	Numeric Rating Scale
PD	Phase difference
RIC	Remote Ischemic Conditioning
WBC	White Blood Cell
NEUT	Neutrophil
LYM	Lymphocyte
RBC	Red Blood Cell
HGB	Hemoglobin
PLT	Platelet
PT	Prothrombin Time
TT	Thrombin Time
APTT	Activated Partial Thromboplastin Time
FIB	Fibrinogen
INR	International Normalized Ratio
PTA	Prothrombin activity
PTR	Prothrombin time ratio
Cr	Creatinine
BUN	Blood Urea Nitrogen

AST	Aspartate Transaminase
ALT	Alanine Transaminase
T-BiL	Total Bilirubin
D-BiL	Direct Bilirubin
I-BiL	Indirect Bilirubin
GLU	Glucose
TG	Triglyceride
TCH	Total Cholesterol
HDL-C	High-Density Lipoprotein Cholesterol
LDL-C	Low-Density Lipoprotein Cholesterol
HbA1c	Glycosylated Hemoglobin
hs-CRP	Hypersensitive C-reactive Protein
Hcy	Homocysteine
NT-proBNP	N-terminal pro-B-type natriuretic peptide

3. Background Information

Stroke is a leading cause of global mortality and disability[1]. Intravenous thrombolysis (IVT) within 4.5 h of stroke onset is recognized as one of the most effective treatments for acute ischemic stroke (AIS)[2-4]. Even so, about 2/3 patients still do not benefit from alteplase intravenous thrombolysis[5-6]. Therefore, how to increase the benefit of IVT, improve the success rate of acute ischemic stroke patients and improve the quality of life of patients have become difficult problems for scientists in various countries.

Cerebral hemodynamics is closely related to the prognosis of ischemic stroke patients. As an important index of cerebral hemodynamics, cerebral autoregulation (CA) refers to a complex process in which intracranial arterioles maintain relatively stable cerebral blood by contraction or relaxation when systemic arterial blood pressure changes, causing changes in intracranial pressure, which can avoid hyperperfusion or hypoperfusion of brain tissue[7-9]. Our previous studies have shown that CA impairment is closely associated with 3-month prognosis in patients with ischemic stroke, and early CA decline is an independent risk factor for poor prognosis.

Remote ischemic conditioning (RIC) is a treatment method that gives intermittent non-lethal reversible ischemic stimulation to a remote vascular bed, organ or tissue to activate endogenous protective mechanisms to protect important ischemic organs[10]. The underlying neuroprotective mechanism triggered by RIC induces gene expression, alters pathways, promotes neurogenesis and blood vessel development, reduces oxidative stress and neuronal apoptosis, and inhibits proinflammatory signals in the ischemic brain[11]. In 2019, our team studied the effects of RIC on CA and blood biomarkers in healthy adults. The results showed that the ability of dynamic CA in healthy adults continued to increase within 6 hours to at least 24 hours after RIC[12]; In addition, at least 2 neuroprotective factors and 4 inflammation-related biological markers in blood changed rapidly within a short time after ischemia, thus providing new evidence for RIPC to induce neuroprotection and improve cerebral vascular function. Recently, our team investigated the effects of a series of RIC on dynamic CA and brain damage-related biomarkers, and showed that after four RIC sessions, the CA was significantly higher than baseline in healthy adults, after 7-day RIC, the CA increase lasts for at least 28 days[13]. At the same time, there were no differences in different serum biomarkers associated with brain damage before and after RIC. The study provides new support for the safety and efficacy of RIC.

However, there is still a lack of studies on the relationship between RIC and cerebral hemodynamics in patients with IVT. Based on the above background, our team designed this study to explore the effect of RIC on cerebral hemodynamics in patients with IVT.

4. Study Objective and Outcomes

4.1 Study Objectives

The primary objective of the study is to determine the effect of RIC on cerebral hemodynamics in patients after IVT.

4.2 Study Outcomes

4.2.1 Primary Efficacy Outcome

The primary endpoint is PD on the affected side at 2 days after IVT.

4.2.2 Secondary Efficacy Outcomes

1) PD on the unaffected side at 2 days after IVT.
2) Gain on the affected side at 2 days after IVT.
3) Gain on the unaffected side at 2 days after IVT.
4) PD on the affected side at 7 days after IVT.
5) PD on the unaffected side at 7 days after IVT.
6) Gain on the affected side at 7 days after IVT.
7) Gain on the unaffected side at 7 days after IVT.
8) NIHSS score at 24 h and 7 days after IVT.
9) Proportion of patients with favorable outcomes (mRS score$\leq$ 2) at 90 days.
10) Final infarct volume.
11) Incidence of hemorrhagic transformation within 24 hours.
12) Serum levels of brain-injury biomarkers at 24 h after IVT.

4.2.3 Safety Outcomes

1) Mortality within 90 days.
2) All adverse events within 90 days.

4.3 Definition of Outcome Parameter

1) Hemorrhagic transformation: Hemorrhagic transformation is determined based on the computer tomography scan conducted 24 hours after IVT, using the European Cooperative Acute Stroke Study (ECASS) criteria[14].

2) Death: All deaths during the study period will be recorded. The cause of each death will be determined based on clinical assessments carried out by the physicians involved in the study.

3) CA assessments: Before the examination, all patients are instructed to relax in the supine position for 10 min in a dedicated quiet examination room with a controlled temperature ranging from 20°C to 24°C. The bilateral cerebral blood flow velocity (CBFV) in the middle cerebral artery is measured using transcranial Doppler (MultiDop X4; DWL, Sipplingen, Germany) sonography. Two 2-MHz probes are fixed with a customized head frame in the bilateral temporal bone window at a depth of 45–60 mm. Simultaneously, arterial blood pressure in the digital artery (measured using a servo-controlled plethysmograph [Finometer Model 1; FMS, Amsterdam, the Netherlands]) is continuously recorded. The end-tidal CO2 is measured using a capnograph (MultiDop X4; DWL) with a nasal cannula. The real-time recordings last 10 min and are stored for further analysis.

4) mRS (see appendix 2).

5) NIHSS (see appendix 3).

5. Sample Size

We estimate that the PD on the affected sides at 2 days after IVT will be 30.12° in the sham-RIC group, according to our pre-experimental work. Here, we estimate a 9-degree improvement with RIC based on the results of preliminary observations. Using a two-tailed t-test to compare the difference between means and assuming $\alpha=0.05$ and statistical power of 80%, the estimate sample size is 80 patients (40 each in the RIC and sham-RIC groups). Considering a 20% loss to follow up, we estimate that 100 patients will be required (50 each in the RIC and sham-RIC groups).

6.Trial Design

This study aims to enroll 100 consecutive patients with acute ischemic stroke who receive IVT therapy. The enrolment follows the principles of randomization and parallel control. The patients will be randomly assigned to RIC group or sham-RIC group, maintaining a 1:1 ratio for a study period of 90 days.

Both groups will receive CA assessments at 2 and 7 days after IVT. The RIC group will undergo RIC at 6 and 18–24 h after IVT, involving pressurization of the unaffected arm with a cuff at a threshold of 200 mmHg (at the same position as the blood pressure measuring cuff) for 5 min, followed by reperfusion for 5 min. This process will be repeated for four cycles, totaling 40 min.

Patients in the sham-RIC group will undergo sham RIC at 6 and 18–24 h after IVT, which includes pressurization of the unaffected arm with a cuff at 60 mmHg (at the same position as the blood pressure measuring cuff) for 5 min, followed by reperfusion for 5 min. This will be repeated for four cycles, totaling 40 min.

Follow-up will continue until 90 days after IVT in both groups to determine the effect of RIC on cerebral hemodynamics in patients after IVT.

7. Randomization

Randomization is established by a researcher who is not involved in the recruitment and assessment of the participants. A random numerical sequence with a 1:1 allocation ratio is computer-generated by an independent biostatistician using SPSS (IBM Corp., Armonk, NY, USA) before starting the study. The randomization code is concealed using sequentially numbered sealed opaque envelopes. After the baseline assessment and obtaining written informed consent, a researcher who is not involved in the data analysis or clinical ratings open the sealed envelope to identify the group to which the participant is allocated (RIC or sham-RIC group) and perform the treatment.

8. The Flow of the Trial

8.1 Visit 1: Screening and Baseline Stage (-4.5-6 h)

The investigator will carefully complete the case screening form, ensuring eligibility per the criteria. If a case does not meet the criteria, the process should be terminated after documenting the reasons. For eligible cases, participants will sign the “Informed Consent Form”, complete the Case Report Form (CRF), and provide the following information:

1) Demographic details: age, sex, nationality, education level, occupation, home address, and contact number, etc.

2) Previous medical and drug treatment history.

a) Previous medical history: history of smoking and alcohol consumption, stroke (if any, specifying the type of stroke), heart disease, hypertension, diabetes, or lipid disorders, as well as

family history of cerebrovascular disease.

b) Concomitant medications: whether the participant took any medication before the onset, such as antiplatelet drugs, anticoagulants, lipid-lowering drugs, antihypertensive drugs, and antidiabetic drugs, etc. The names, durations, and doses of the medications used, as well as the dosage, total amount, and duration of drugs used in the current episode, should be recorded.

3) Present medical history: onset time (last known as normal), premorbid mRS score, clinical presentation, physical examination (height, weight, waist circumference, pre-thrombolysis NIHSS score).

4) Vital signs: heart rate, blood pressure, respiration, and temperature at baseline.

5) Blood sample collection before thrombolysis (optional):

a) Blood samples (two yellow blood collection tube, one purple blood collection tube):

b) Yellow tube: Separate serum within 2 h of blood collection. For short-term storage, place the serum in a freezer at -80°C. For long-term storage, place the serum in liquid nitrogen.

c) Purple tube: Separate plasma and cellular components as soon as possible after blood collection. For short-term storage, place in a -80°C freezer; for long-term storage, place in liquid nitrogen.

6) Results of the first brain computed tomography (CT)/magnetic resonance imaging (MRI), electrocardiogram (ECG), etc.

7) Laboratory tests:

a) Blood routine examination: White Blood Cell Count (WBC), Absolute Neutrophil Count (NEUT#), Absolute Lymphocyte Count (LYM#), Red Blood Cell Count (RBC), Hemoglobin (HGB), Platelet Count (PLT).

b) Routine coagulation examination: Prothrombin Time (PT), Thrombin Time (TT), Activated Partial Thromboplastin Time (APTT), Fibrinogen (FIB), International Normalized Ratio (INR), Prothrombin activity (PTA), Prothrombin time ratio (PTR).

c) Renal function tests (ask about a history of kidney disease; if no relevant disease occurs, results are not required but should be completed within 24 h): Creatinine (Cr) and blood urea nitrogen (BUN)/urea.

d) Liver function tests (ask about a history of liver disease; if no relevant disease occurs, results are not required but should be completed within 24 h): Aspartate transaminase (AST), alanine transaminase (ALT), total bilirubin (T-BiL), direct bilirubin (D-BiL), indirect bilirubin (I-BiL).

e) Fingerstick blood glucose: Glucose (GLU).

f) Other laboratory texts such as glycosylated Hemoglobin (HbA1c), hypersensitive C-reactive Protein (hs-CRP), Homocysteine (Hcy), etc.

8) Fill in the above information into the CRF for Visit 1.

8.2 Visit 2: Treatment Stage (6-24 h)

Collect and record the following information:

1) Concurrent medications within 24 h after IVT, along with vital signs, NIHSS score at 24 h after IVT.

2) Brain CT result (re-examined at 24 h after IVT), whether hemorrhagic transformation.

3) Blood sample collection:

a) Blood samples (two yellow blood collection tube, one purple blood collection tube:):

b) Yellow tube: Separate serum within 2 h of blood collection. For short-term storage, place

the serum in a freezer at -80°C. For long-term storage, place the serum in liquid nitrogen.

c) Purple tube: Separate plasma and cellular components as soon as possible after blood collection. Short-term storage: place in a freezer at -80°C; long-term storage: place in liquid nitrogen.

4) Complete two times of RIC treatment at 6 and 18-24 h after IVT, recording the treatment site, number of cycles, time, and the occurrence of adverse events (AEs).

5) Fill in the above information into the CRF for Visit 2.

During this period, the investigator should closely observe the patient's condition and note any AEs or recurrence/progression of stroke; if any of these occur, detailed records should be made, and a decision on whether to terminate the study should be made based on established standards. Except for death, withdrawal of informed consent, or a request from the investigator administrative department/ethics committee to terminate the study, follow-up must be completed for patients who discontinue the study.

8.3 Visit 3: Follow-up Stage (1-7 d)

1) Concurrent medications within the hospitalization; recording vital signs, NIHSS score of participants at 7 days after IVT or at discharge. If the patient is still in the hospital, record the scores at 7 days; if the patient is discharged before 7 days, record the corresponding scores at the time of discharge; if the patient is discharged after 7 days, record the corresponding scores at 7 days.

2) Patients in both groups undergo CA assessments at 2 and 7 days after IVT.

3) Blood pressure variability and heart rate variability results are recorded at 2 and 7 days after IVT.

4) Brain diffusion-weighted magnetic resonance imaging (MRI+DWI) scan results after two times of RIC treatment, recording the infarct location and volume.

5) Discharge diagnosis and medications information.

6) Fill the above information into the CRF for Visit 3.

During this period, the investigator should closely observe the patient's condition and note any AEs or recurrence/progression of stroke; if any of these occur, detailed records should be made, and a decision on whether to terminate the study should be made based on established standards. Except for death, withdrawal of informed consent, or a request from the investigator administrative department/ethics committee to terminate the study, follow-up must be completed for patients who discontinue the study.

8.4 Visit 4: Follow-up Stage (90±3 d)

This trial requires once follow-up visit after discharge, which will be conducted via telephone and video. The follow-up visit is scheduled at 90±3 days after IVT. During follow-up, it is essential to inquire about the occurrence of endpoints, deaths, and AEs. Additionally, details regarding the patient's medication regimen, and mRS score, should be obtained and documented.

Participants have the right to withdraw from the study at any point. Participants are required to complete the study-designed follow-up visit. Except for death, withdrawal of informed consent, or a request from the investigator administrative department/ethics committee to terminate the study, follow-up must be completed for patients who discontinue the study.

9. Combination Medications

This study does not restrict any combination medications, but all combination medications or alterations in dosing regimens must be documented in the CRF. Each comorbid medication should

include details like the drug name, dosage, purpose, and frequency/duration of use for analysis and reporting during the summary.

10. Patient Population

10.1 Inclusion Criteria

1) Age ≥ 18 and < 80 years, both sexes.
2) A clear clinical diagnosis of acute ischemic stroke and treatment with standard rt-PA (0.9 mg/kg) IVT within 4.5 h of stroke onset.
3) Pre-onset modified Rankin Scale (mRS) score ≤1.
4) Baseline National Institute of Health Stroke Scale (NIHSS) score ≥5 and ≤25.
5) Glasgow Coma Scale score ≥8.

10.2 Exclusion Criteria

1) Having received bridging therapy (IVT plus mechanical thrombectomy).
2) Previous history of atrial fibrillation or electrocardiographic evidence of atrial fibrillation.
3) Contraindications to RIC treatment or previous RIC treatment or similar treatment.
4) Pregnancy or breastfeeding.
5) Life expectancy of ≤3 months or inability to complete the study for other reasons.
6) Unwillingness to be followed up or poor treatment compliance or participation in other clinical studies.
7) Had insufficient bilateral temporal bone windows for insonation of the middle cerebral artery.

10.3 Suspension Criteria

Trial suspension means that participants who have been enrolled in the study cannot continue during the study, and treatment prescribed by the protocol is discontinued early, but safety or post-treatment visits can continue. The goal is to protect participants' rights and interests, ensure trial quality, and prevent unnecessary economic losses. Suspension may occur under the following circumstances:

1) Serious safety issues occur during the trial, and the suspension is deemed necessary by the investigator.
2) Trial should be suspended when participants develop unstable vital signs or coma after treatment.
3) The participants experience complications, AEs, or special physiological changes during the trial that may affect their efficacy and safety assessments.
4) Significant trial design defects or major deviations are identified during implementation.
5) Regulatory authorities or ethics committees request study suspension.
6) Other reasons.

10.4 Dropout Criteria

Determination of dropout: Participants who signed an informed consent form and qualified during screening but does not complete the entire clinical trial should be considered dropouts, including voluntary withdrawal, revoked consent, or being lost to follow-up. The investigator should record the reason for dropout in CRF and retain source documents for reference. The dropout rate should not exceed 20%.

11. Adverse Events

11.1 Definition of Adverse Events

Any adverse medical event that occurs from the time of randomization until the last follow-up, whether related to the study treatment, is considered an AE. Following the regulatory requirements, any undesired event that occurs after a participant has signed an informed consent form to participate in a clinical study until its completion or withdrawal from that clinical study is an AE. The investigator should report all AEs directly observed by the physician or spontaneously reported by the participants, using concise medical terminology. AEs are reactions that are directly or indirectly related to the treatment method. In the case of events for which it is difficult to determine the relationship and prevent omission, any AEs should be recorded in a CRF documenting the time of occurrence, severity, duration, measures taken, and regression of the AE, with a separate statement of its possible relevance to the treatment.

11.2 Observation, Assessment, Recording, and Reporting of Adverse Events

Safety assessments will be conducted for all participants who receive treatment at least once. This includes recording the name of the AE, severity, outcome, relationship with treatment, and measures taken. The following should also be recorded: death, recurrent stroke, termination of treatment for any reason, abnormal laboratory test results, changes in vital signs, and changes in physical examination results. The researcher will assess, sign, and date the relationship with the investigational treatment. AEs will be recorded from randomization until the last follow-up. If an AE occurs, follow-up should continue until it disappears, the patient recovers to baseline levels, or becomes clinically insignificant. All AEs during the study will be recorded in the designated CRF AE form.

11.3 Severity Grading Criteria for Adverse Events

AEs will be reported and graded according to the National Cancer Institute’s (NCI) Common Terminology Criteria for Adverse Events (CTCAE) version 5.0. Severity is classified as mild, moderate, severe, extremely severe or death and defined as follows:

1) Mild: No or mild symptoms or diseases, only clinically or diagnostically observed, rapid recovery after stopping the study intervention, and no treatment required.

2) Moderate: Causing temporary damage requires minor, local, or non-invasive treatment; easy recovery; and does not require an extended hospital stay.

3) Severe: Causes serious or medically significant but not immediately life-threatening damage requiring extended hospitalization (more than 7 days) or permanent damage to systems/organs.

4) Extremely severe: Life-threatening conditions (with symptoms such as suffocation, shock, or coma) require emergency rescue treatment.

5) Death: Death related to the AE.

11.4 Association between Adverse Events and Study Treatment

Investigators should assess the potential association between AEs and study treatment, as well as concomitant drug use, based on the following 5-level classification criteria:

1) Related: The occurrence of AEs follows a reasonable temporal sequence with the study treatment; AEs match known types of adverse reactions to the suspected study treatment; the severity of AEs improves or disappears after stopping or reducing the study treatment; the AE reappears upon re-administration of the study treatment and cannot be explained by the participant’s disease or

concomitant drug use.
2) Probably related: The occurrence of AEs follows a reasonable temporal sequence with the study treatment; AEs match known types of adverse reactions to the suspected study treatment; the severity of AEs can or cannot be improved or resolved after stopping or reducing the study treatment; the AE can or cannot recur upon re-administration of the study treatment; the patient’s clinical condition or other treatments (such as concomitant drug use) could also produce an AE.
3) Probably unrelated: The occurrence of AEs follows a reasonable temporal sequence with the study treatment; AEs do not match known types of adverse reactions to the suspected study treatment; the severity of AEs can or cannot be improved or resolved after stopping or reducing the study treatment; the AE can or cannot recur upon re-administration of the study treatment; and the patient’s clinical condition or other treatments (such as concomitant drug use) could also produce an AE.
4) Unrelated: The occurrence of AEs does not follow a reasonable temporal sequence with the study treatment; AEs do not match known types of adverse reactions to the suspected study treatment; the severity of AEs did not improve or disappear after stopping or reducing the study treatment; the AE does not occur upon re-administration of the study treatment; the patient’s clinical condition or other treatments (such as concomitant drug use) could produce AEs.
5) Unjudged: A judgment cannot be made due to contradictory information or the fact that the information cannot be supplemented and verified.

11.5 Management of Adverse Events

1) When an AE is discovered, researchers should decide whether to suspend observation based on the patient’s condition. In the case of a serious AE, the clinical research unit must take immediate measures to protect the safety of the participants. All AEs will be tracked and investigated in detail, and the process and results of handling will be recorded until proper resolution is achieved or conditions stabilized. If the laboratory test results are abnormal, follow-up should be continued until the results are normal. Follow-up methods can be selected based on the severity of the AEs, including hospitalizations, outpatient visits, home visits, telephone calls, and correspondence.
2) Emergencies, including serious (particularly life-threatening) AEs, should be reported to the provincial, autonomous region, and municipal adverse reaction monitoring professional institutions using the fastest communication method.

11.6 Serious Adverse Events

1) Serious AEs are defined as AEs that lead to the following results:

a) Death

b) Life-threatening (refers to a situation where the subject is at risk of death when an AE occurs, not events that might lead to death under hypothetical worsening conditions)

c) Requires hospitalization or prolongation of hospitalization

d) Causes persistent or significant disability or dysfunction

e) Events that the investigator judges as medical events can also be judged as serious AEs

2) Suppose a subject experiences a serious AE during the trial, regardless of whether it is related to the study treatment. In that case, the investigator will immediately implement appropriate treatment measures to ensure the safety of the subject. The event must be reported within 24 hours to the principal research center, local ethics committee. Simultaneously, the investigator must complete a

serious AE.

3) Serious AEs that are not resolved when the study ends or the subject withdraws prematurely must be followed up until any of the following conditions are met:

a) The event disappears or is resolved;

b) The event stabilizes;

c) The event returns to the baseline level (if baseline values are available);

d) The event alleviates to a clinically insignificant level;

e) The event can be attributed to a drug other than the study treatment, factors unrelated to the study, or when more information is unlikely to be obtained (the patient or caregiver refuses to provide more information, or there is evidence that after the best effort has been made, the patient is still lost to follow-up).

12. Statistical Analysis

Intention-to-treat (ITT) population: According to the basic principle of intention-to-treat (ITT), all randomly assigned patients will be included in the ITT population. The ITT population will be the main analysis population for the efficacy endpoint, and subjects will be analysed according to the treatment group to which they are assigned at randomization.

Per-Protocol (PP) population: The PP population is a subset of ITT population, including all randomized subjects who have been treated in the study without major protocol deviations that may significantly impact the interpretation of efficacy results.

Safety population: The safety population includes all patients who received at least one RIC or sham-RIC treatment.

13. Appendix

13.1 Appendix 1: Study Flow Chart

<table>
<tr><th>Stage Item</th><th>Screening</th><th>Treatment Stage</th><th colspan="2">Follow-up</th></tr>
<tr><th>Visit</th><th>Visit 1</th><th>Visit 2</th><th>Visit 3</th><th>Visit 4</th></tr>
<tr><th>Time</th><th>-4.5 h-6 h</th><th>6 h-24 h</th><th>1 d-7 d</th><th>90±3 d</th></tr>
<tr><td>Signed informed consent</td><td>×</td><td></td><td></td><td></td></tr>
<tr><td>Demographic characteristics</td><td>×</td><td></td><td></td><td></td></tr>
<tr><td>Medical history</td><td>×</td><td></td><td></td><td></td></tr>
<tr><td>Combination medications</td><td>×</td><td>×</td><td>×</td><td>×</td></tr>
<tr><td>Present medical history</td><td>×</td><td></td><td></td><td></td></tr>
<tr><td>Vital signs</td><td>×</td><td>×</td><td>×</td><td></td></tr>
<tr><td>NIHSS score</td><td>×</td><td>×</td><td>×</td><td></td></tr>
<tr><td>mRS score</td><td>×</td><td></td><td></td><td>×</td></tr>
<tr><td>Laboratory tests</td><td colspan="3">×</td><td></td></tr>
<tr><td>ECG</td><td>×</td><td></td><td></td><td></td></tr>
<tr><td>Brain CT scan</td><td>×</td><td>×</td><td></td><td></td></tr>
<tr><td>Brain MRI+DWI</td><td></td><td></td><td>×</td><td></td></tr>
<tr><td>Blood specimen</td><td>× (optional)</td><td>×</td><td></td><td></td></tr>
<tr><td>RIC</td><td></td><td>×</td><td></td><td></td></tr>
<tr><td>CA</td><td></td><td></td><td>×</td><td></td></tr>
<tr><td>Discharge diagnosis</td><td></td><td></td><td>×</td><td></td></tr>
<tr><td>Adverse events</td><td>×</td><td>×</td><td>×</td><td>×</td></tr>
</table>

0 h: Immediately after receiving intravenous thrombolysis.

Laboratory tests include routine blood tests, coagulation routine tests, kidney function, liver function, HbA1c, hs-CRP, hcy, fasting blood glucose, NT-proBNP, uric acid, erythrocyte sedimentation rate, serum cysteine c, blood Lipids, serum ion, etc.

Abbreviation: RIC, remote ischemic conditioning; CA, cerebral autoregulation; NIHSS, National Institute of Health Stroke Scale; mRS: modified Rankin Scale; ECG, Electrocardiograph; CT, computed tomography; MRI+DWI, diffusion-weighted magnetic resonance imaging.

13.2 Appendix 2: Modified Rankin Scale

The mRS is used to measure the patient's functional recovery post-stroke. Please note that only symptoms that occurred after the stroke are considered. A patient is considered able to walk independently if they could walk without external help despite the use of assistive devices.

In cases where both levels appear applicable and further questioning would not lead to a correct choice, a more severe level should be chosen.

Grade	Description
0	No symptoms
1	Symptoms without any incapacity (able to perform all usual activities)
2	Mild incapacity (unable to perform all usual activities but able to look after his/her affairs alone)
3	Moderate incapacity (requires assistance but walks alone)
4	Severe incapacity (requires assistance for walking and physical body needs)
5	Severe incapacity (bedbound, incontinent, permanent surveillance required)
6	Death

13.3 Appendix 3: National Institutes of Health Stroke Scale

Check	Scores	Point
**1a. Level of Consciousness (0-3):** Verbal or painful stimulation of the patient (left to the choice of the examiner)	0 = Aware, responds briskly.<br>1 = Responds to minor stimuli.<br>2 = Responds to repeated or painful stimuli.<br>3 = Stereotypic or flaccid response.	
**1b. Consciousness level (0-2):**<br>- "How old are you?"<br>- "What month is this?"	0 = Correct answer to the 2 questions.<br>1 = One single correct answer or intubated, or severe dysarthria or language barrier.<br>2 = No correct response or aphasic.	
**1c. Consciousness-commands (0-2):**<br>- "Open and close your eyes"<br>- "Close and open your hand"<br>To be imitated if the command is not followed	0 = Performs both tasks correctly.<br>1 = Performs one task correctly.<br>2 = Performs neither task correctly.	
**2. Horizontal oculomotilities (0-2):** Test voluntary and reflex horizontal movements without a heat test (finger following): "Follow my finger".	0 = Normal oculomotility.<br>1 = Conjugate deviation of the eyes which may be reduced by voluntary or reflex activity or isolated damage to a cranial nerve.<br>2 = Complete paralysis of the side.	
**3. Visual field (0-3):**<br>Test the visual field by quadrants (upper and lower) using finger counting or if necessary the menace blink reflex.	0 = Normal visual field.<br>1 = Partial HLH or loss of vision.<br>2 = Complete HLH.<br>3 = Dual HLH or cortical blindness.	
**4. Facial paralysis (0-3):**<br>"Show me your teeth, raise your eyebrows and close your eyes"<br>*To be imitated if command not performed or Pierre Marie and Foix manoeuvre.*	0 = Normal.<br>1 = Slight central facial paralysis (FP).<br>2 = Clear central PF (total inferior).<br>3 = Dual PF or total PF.	
**5. Upper limb motility:**<br>5.1 "Stretch out your arm and left hand" (0-4).<br>5.2 "Stretch out your arm and right hand" (0-4) for 10 seconds.	0 = Normal.<br>1 = Resists a weight (arm falls within 10 sec).<br>2 = Does not resist (arm touches the bed before 10 sec).<br>3 = Does not lift limb (contraction without movement).<br>4 = No movement.	5a Left upper extremity<br>5b Right upper limb

**6. Lower limb motility:**<br>6.1 "Stretch out your left leg" (30°) (0-4)<br>6.2 "Stretch out your right leg" (0-4) for 5 seconds	0 = Normal.<br>1 = Resists a weight (arm falls within 5 sec).<br>2 = Does not resist (arm touches the bed before 5 sec).<br>3 = Does not lift limb (contraction without movement).<br>4 = No movement.	6a Left lower extremity<br>6b Right lower extremity
**7. Limb ataxia (0-2):**<br>"Place your index finger on your nose" "Place your heel on the opposite knee" *Bilateral manoeuvre*	0 = Normal or impossible because of paralysis or aphasia.<br>1 = Ataxia of one limb.<br>2 = Ataxia of 2 limbs.<br>9 = Amputation or joint block.	
**8. Sensitivity (0-2):**<br>Examine sensation to pin prick or withdrawal following nociceptive stimulation if confused or aphasic (arm, leg, face, trunk, bilaterally).	0 = Normal.<br>1 = Hypoesthesia or aphasia or stupor.<br>2 = Severe to total deficit.	
**9. Language (0-3):**<br>"Describe the following scene"<br>"Tell me the name of these objects" "Read these phrases"<br>If visual disturbance, identify the objects in a hand and have the words repeated.<br>*Assess writing in an intubated patient*	0 = Normal.<br>1 = Aphasic but communicates.<br>2 = Quasi-impossible communication.<br>3 Total aphasia, mutism or coma.	
**10. Dysarthria (0-2):**<br>"Repeat the following words" (cf. text 2 p 3)<br>***including** in aphasic patients.*	0 = Normal articulation.<br>1 = Comprehensible.<br>2 = Incomprehensible, anarthria or mutism.<br>9 = Intubation or mechanical obstruction.	
**11. Extinction or negligence:**<br>Test bilateral simultaneous sensitivity<br>Test perception in both temporal visual fields simultaneously Investigate for anosognosia and visuospatial neglect.	0 = No extinction or complete HLH (if sensory extinction) and vice versa or aphasia or gives the impression of understanding.<br>1 = Extinction of one modality.<br>2 = Extinction of several modalities or visuospatial neglect or anosognosia.	

14. Reference

1. Roth GA, Mensah GA, Johnson CO, et al. Global Burden of Cardiovascular Diseases and Risk Factors, 1990-2019: Update From the GBD 2019 Study. J Am Coll Cardiol. 2020;76(25):2982-3021. doi:10.1016/ j. jacc.2020.11.010.

2. Powers WJ, Rabinstein AA, Ackerson T, et al. Guidelines for the Early Management of Patients With Acute Ischemic Stroke: 2019 Update to the 2018 Guidelines for the Early Management of Acute Ischemic Stroke: A Guideline for Healthcare Professionals From the American Heart Association/American Stroke Association. Stroke. 2019;50(12): e344-e418. doi:10.1161/STR.0000000000000211.

3. Berge E, Whiteley W, Audebert H, et al. European Stroke Organisation (ESO) guidelines on intravenous thrombolysis for acute ischaemic stroke. Eur Stroke J. 2021;6(1): I-LXII. doi:10.1177/2396987321989865.

4. Chinese Society of Neurology; Chinese Stroke Society. Chinese Guideline for Endovascular Treatment of Acute Ischemic Stroke 2018. Chin J Neurol. 2018; 51:666-682. doi:10.3969/ j. issn.1673-5765.2018.07.014.

5. Hankey GJ. Stroke. Lancet. 2017;389(10069):641-654. doi:10.1016/S0140-6736(16)30962-X.

6. Emberson J, Lees KR, Lyden P, et al. Effect of treatment delay, age, and stroke severity on the effects of intravenous thrombolysis with alteplase for acute ischaemic stroke: a meta-analysis of individual patient data from randomised trials. Lancet. 2014;384(9958):1929-1935. doi:10.1016/S0140-6736(14)60584-5.

7. MacGregor DG, Carswell HV, Graham DI, et al. Impaired cerebral autoregulation 24 h after induction of transient unilateral focal ischaemia in the rat. Eur J Neurosci. 2000;12(1):58-66.

8. Fan JL, O'Donnell T, Lanford J, et al. Dietary nitrate reduces blood pressure and cerebral artery velocity fluctuations and improves cerebral autoregulation in transient ischemic attack patients. J Appl Physiol (1985). 2020;129(3):547-57.

9. Aaslid R LK, Sorteberg W, Nornes H. Cerebral autoregulation dynamics in humans. Stroke. 1989;20(1):45-52.

10. Murry CE, Jennings RB, Reimer KA. Preconditioning with ischemia: a delay of lethal cell injury in ischemic myocardium. Circulation. 1986;74(5):1124-1136. doi:10.1161/01. cir. 74.5.1124.

11. Weir P, Maguire R, O'Sullivan SE, England TJ. A meta-analysis of remote ischaemic conditioning in experimental stroke. J Cereb Blood Flow Metab. 2021;41(1):3-13. doi: 10.1177/0271678X20924077.

12. Guo ZN, Guo WT, Liu J, et al. Changes in cerebral autoregulation and blood biomarkers after remote ischemic preconditioning. Neurology. 2019;93(1): e8-e19.

13. Qu Y, Zhang P, He QY, et al. The Impact of Serial Remote Ischemic Conditioning on Dynamic Cerebral Autoregulation and Brain Injury Related Biomarkers. Front Physiol. 2022; 13:835173.

14. Hacke W, Kaste M, Fieschi C, et al. Intravenous thrombolysis with recombinant tissue plasminogen activator for acute hemispheric stroke. The European Cooperative Acute Stroke Study (ECASS). JAMA. 1995 Oct 4;274(13):1017-25.

RICCH-IVT Study Protocols

Effect of Remote Ischemic Conditioning on Cerebral Hemodynamics in Patients After Intravenous Thrombolysis

(RICCH-IVT)

Original protocol (Version 1.0, 26 December 2022)

Contents

1. Study Synopsis **31**

2. List of Abbreviations **34**

3. Background Information **36**

4. Study Objectives and Outcomes **37**

4.1 Study Objectives 37

4.2 Study Outcomes 37

4.3 Defination of Outcome Parameter 37

5. Sample Size **38**

6. Trial Design **38**

7. Randomization **38**

8. The Flow of the Trial **38**

8.1 Visit 1: Screening and Baseline Stage (-4.5-6 h) 38

8.2 Visit 2: Treatment Stage (6-24 h) 39

8.3 Visit 3: Follow-up Stage (1-10 d) 40

8.4 Visit 4: Follow-up Stage (90±3 d) 40

9. Combination Medications **40**

10. Patient Population **41**

10.1 Inclusion Criteria 41

10.2 Exclusion Criteria 41

10.3 Suspension Criteria 41

10.4 Dropout Criteria 41

11. Adverse Events **42**

11.1 Definition of Adverse Events 42

11.2 Observation, Assessment, Recording, and Reporting of Adverse Events 42

11.3 Severity Grading Criteria for Adverse Events 42

11.4 Association between Adverse Events and Study Treatment 42

11.5 Management of Adverse Events 43

11.6 Serious Adverse Events 43

12. Statistical Analysis **44**

13. Appendix **45**

13.1 Appendix1: Study Flow Chart 45

13.2 Appendix 2: Modified Rankin Scale 46

13.3 Appendix 3: National Institutes of Health Stroke Scale..47

14. Reference 49

1. Study Synopsis

**Study Tiltle**	Effect of Remote Ischemic Conditioning on Cerebral Hemodynamics in Patients After Intravenous Thrombolysis (RICCH-IVT)
**Principal Research Center**	The First Hospital of Jilin University
**Principal Investigator**	Pro. Yi Yang, M.D, Ph.D. Stroke Center, Department of Neurology, the First Hospital of Jilin University, Chang Chun, China
**Objective**	To determine the effect of remote ischemic conditioning on cerebral hemodynamics in patients after intravenous thrombolysis.
**Trial Design**	This study aims to enroll 100 consecutive patients with acute ischemic stroke who receive intravenous thrombolytic (IVT) therapy. The enrolment follows the principles of randomization and parallel control. The patients will be randomly assigned to remote ischemic conditioning (RIC) group or sham-RIC group, maintaining a 1:1 ratio for a study period of 90 days.<br>Both groups will receive cerebral autoregulation (CA) assessments at 1-2 and 7-10 days after IVT. The RIC group will undergo RIC at 6 and 18–24 h after IVT, involving pressurization of the unaffected arm with a cuff at a threshold of 200 mmHg (at the same position as the blood pressure measuring cuff) for 5 min, followed by reperfusion for 5 min. This process will be repeated for four cycles, totaling 40 min.<br>Patients in the sham-RIC group will undergo sham RIC at 6 and 18–24 h after IVT, which includes pressurization of the unaffected arm with a cuff at 60 mmHg (at the same position as the blood pressure measuring cuff) for 5 min, followed by reperfusion for 5 min. This will be repeated for four cycles, totaling 40 min.<br>Follow-up will continue until 90 days after IVT in both groups to determine the effect of RIC on cerebral hemodynamics in patients after IVT.
**Outcomes**	**Primary Efficacy Outcome:**<br>The primary endpoint is phase difference (PD, the main parameter of CA) on the affected side at 1-2 days after IVT.<br><br>**Secondary Efficacy Outcomes:**<br>1) PD on the unaffected side at 1-2 days after IVT.<br>2) Gain (the parameter of CA) on the affected side at 1-2 days after IVT.<br>3) Gain on the unaffected side at 1-2 days after IVT.<br>4) PD on the affected side at 7-10 days after IVT.

	5) PD on the unaffected side at 7-10 days after IVT.<br>6) Gain on the affected side at 7-10 days after IVT.<br>7) Gain on the unaffected side at 7-10 days after IVT.<br>8) NIHSS score at 24 h and 7 days after IVT.<br>9) Proportion of patients with favorable outcomes (mRS score≤ 2) at 90 days.<br>10) Final infarct volume.<br>11) Incidence of hemorrhagic transformation within 24 hours.<br>12) Serum levels of brain-injury biomarkers at 24 h after IVT.<br><br>**Safety Outcomes:**<br>1) Mortality within 90 days.<br>2) All adverse events within 90 days.
**Inclusion Criteria**	1) Age ≥ 18 and < 80 years, both sexes.<br>2) A clear clinical diagnosis of acute ischemic stroke and treatment with standard rt-PA (0.9 mg/kg) IVT within 4.5 h of stroke onset.<br>3) Pre-onset modified Rankin Scale (mRS) score ≤1.<br>4) Baseline National Institute of Health Stroke Scale (NIHSS) score ≥5 and ≤25.<br>5) Glasgow Coma Scale score ≥8.
**Exclusion Criteria**	1) Having received bridging therapy (IVT plus mechanical thrombectomy).<br>2) Previous history of atrial fibrillation or electrocardiographic evidence of atrial fibrillation.<br>3) Contraindications to RIC treatment or previous RIC treatment or similar treatment.<br>4) Pregnancy or breastfeeding.<br>5) Life expectancy of ≤3 months or inability to complete the study for other reasons.<br>6) Unwillingness to be followed up or poor treatment compliance or participation in other clinical studies.<br>7) Had insufficient bilateral temporal bone windows for insonation of the middle cerebral artery.
**Interventions**	All subjects will receive standard clinically routine treatments for acute ischemic stroke and other underlying diseases.<br><br>**RIC group:** RIC (200 mmHg) combined with alteplase IVT group<br>1) Method: external use, cuff fixed on the unaffected upper arm (same position as the blood pressure measuring cuff).<br>2) Treatment pressure: 200 mmHg.<br>3) Treatment mode: 5 min of pressurization followed by 5 min of rest as a cycle, repeated for four cycles, with a total duration

	of 40 min.<br>4) Treatment cycle: 6 and 18–24 h after IVT.<br><br>**Sham-RIC group:** Sham RIC (60 mmHg) combined with alteplase IVT group<br>1) Method: external use, cuff fixed on the unaffected upper arm (same position as the blood pressure measuring cuff).<br>2) Treatment pressure: 60 mmHg.<br>3) Treatment mode: 5 min of pressurization, followed by 5 min of rest as a cycle, repeated for four cycles, with a total duration of 40 min.<br>4) Treatment cycle: 6 and 18–24 h after IVT.
**Follow-up**	Follow-up will continue until 90 days after IVT in both groups.
**Sample Size**	100

2. List of Abbreviations

Abbreviations	Full title
AIS	Acute Ischemic Stroke
AE	Adverse Event
CRF	Case Report Form
CT	Computed Tomography
DWI	Diffusion Weighted Imaging
ICH	Intracranial Hemorrhage
IVT	Intravenous Thrombolysis
mRS	modified Rankin Scale
MRI	Magnetic Resonance Imaging
NIHSS	National Institute of Health Stroke Scale
NRS	Numeric Rating Scale
PD	Phase difference
RIC	Remote Ischemic Conditioning
WBC	White Blood Cell
NEUT	Neutrophil
LYM	Lymphocyte
RBC	Red Blood Cell
HGB	Hemoglobin
PLT	Platelet
PT	Prothrombin Time
TT	Thrombin Time
APTT	Activated Partial Thromboplastin Time
FIB	Fibrinogen
INR	International Normalized Ratio
PTA	Prothrombin activity
PTR	Prothrombin time ratio
Cr	Creatinine
BUN	Blood Urea Nitrogen

AST	Aspartate Transaminase
ALT	Alanine Transaminase
T-BiL	Total Bilirubin
D-BiL	Direct Bilirubin
I-BiL	Indirect Bilirubin
GLU	Glucose
TG	Triglyceride
TCH	Total Cholesterol
HDL-C	High-Density Lipoprotein Cholesterol
LDL-C	Low-Density Lipoprotein Cholesterol
HbA1c	Glycosylated Hemoglobin
hs-CRP	Hypersensitive C-reactive Protein
Hcy	Homocysteine
NT-proBNP	N-terminal pro-B-type natriuretic peptide

3. Background Information

Stroke is a leading cause of global mortality and disability[1]. Intravenous thrombolysis (IVT) within 4.5 h of stroke onset is recognized as one of the most effective treatments for acute ischemic stroke (AIS)[2-4]. Even so, about 2/3 patients still do not benefit from alteplase intravenous thrombolysis[5-6]. Therefore, how to increase the benefit of IVT, improve the success rate of acute ischemic stroke patients and improve the quality of life of patients have become difficult problems for scientists in various countries.

Cerebral hemodynamics is closely related to the prognosis of ischemic stroke patients. As an important index of cerebral hemodynamics, cerebral autoregulation (CA) refers to a complex process in which intracranial arterioles maintain relatively stable cerebral blood by contraction or relaxation when systemic arterial blood pressure changes, causing changes in intracranial pressure, which can avoid hyperperfusion or hypoperfusion of brain tissue[7-9]. Our previous studies have shown that CA impairment is closely associated with 3-month prognosis in patients with ischemic stroke, and early CA decline is an independent risk factor for poor prognosis.

Remote ischemic conditioning (RIC) is a treatment method that gives intermittent non-lethal reversible ischemic stimulation to a remote vascular bed, organ or tissue to activate endogenous protective mechanisms to protect important ischemic organs[10]. The underlying neuroprotective mechanism triggered by RIC induces gene expression, alters pathways, promotes neurogenesis and blood vessel development, reduces oxidative stress and neuronal apoptosis, and inhibits proinflammatory signals in the ischemic brain[11]. In 2019, our team studied the effects of RIC on CA and blood biomarkers in healthy adults. The results showed that the ability of dynamic CA in healthy adults continued to increase within 6 hours to at least 24 hours after RIC[12]; In addition, at least 2 neuroprotective factors and 4 inflammation-related biological markers in blood changed rapidly within a short time after ischemia, thus providing new evidence for RIPC to induce neuroprotection and improve cerebral vascular function. Recently, our team investigated the effects of a series of RIC on dynamic CA and brain damage-related biomarkers, and showed that after four RIC sessions, the CA was significantly higher than baseline in healthy adults, after 7-day RIC, the CA increase lasts for at least 28 days[13]. At the same time, there were no differences in different serum biomarkers associated with brain damage before and after RIC. The study provides new support for the safety and efficacy of RIC.

However, there is still a lack of studies on the relationship between RIC and cerebral hemodynamics in patients with IVT. Based on the above background, our team designed this study to explore the effect of RIC on cerebral hemodynamics in patients with IVT.

4. Study Objective and Outcomes

4.1 Study Objectives

The primary objective of the study is to determine the effect of RIC on cerebral hemodynamics in patients after IVT.

4.2 Study Outcomes

4.2.1 Primary Efficacy Outcome

The primary endpoint is PD on the affected side at 1-2 days after IVT.

4.2.2 Secondary Efficacy Outcomes

1) PD on the unaffected side at 1-2 days after IVT.
2) Gain on the affected side at 1-2 days after IVT.
3) Gain on the unaffected side at 1-2 days after IVT.
4) PD on the affected side at 7-10 days after IVT.
5) PD on the unaffected side at 7-10 days after IVT.
6) Gain on the affected side at 7-10 days after IVT.
7) Gain on the unaffected side at 7-10 days after IVT.
8) NIHSS score at 24 h and 7 days after IVT.
9) Proportion of patients with favorable outcomes (mRS score$\leq$ 2) at 90 days.
10) Final infarct volume.
11) Incidence of hemorrhagic transformation within 24 hours.
12) Serum levels of brain-injury biomarkers at 24 h after IVT.

4.2.3 Safety Outcomes

1) Mortality within 90 days.
2) All adverse events within 90 days.

4.3 Definition of Outcome Parameter

1) Hemorrhagic transformation: Hemorrhagic transformation is determined based on the computer tomography scan conducted 24 hours after IVT, using the European Cooperative Acute Stroke Study (ECASS) criteria[14].

2) Death: All deaths during the study period will be recorded. The cause of each death will be determined based on clinical assessments carried out by the physicians involved in the study.

3) CA assessments: Before the examination, all patients are instructed to relax in the supine position for 10 min in a dedicated quiet examination room with a controlled temperature ranging from 20°C to 24°C. The bilateral cerebral blood flow velocity (CBFV) in the middle cerebral artery is measured using transcranial Doppler (MultiDop X4; DWL, Sipplingen, Germany) sonography. Two 2-MHz probes are fixed with a customized head frame in the bilateral temporal bone window at a depth of 45–60 mm. Simultaneously, arterial blood pressure in the digital artery (measured using a servo-controlled plethysmograph [Finometer Model 1; FMS, Amsterdam, the Netherlands]) is continuously recorded. The end-tidal CO2 is measured using a capnograph (MultiDop X4; DWL) with a nasal cannula. The real-time recordings last 10 min and are stored for further analysis.

4) mRS (see appendix 2).

5) NIHSS (see appendix 3).

5. Sample Size

We estimate that the PD on the affected sides at 1-2 days after IVT will be 30.12° in the sham-RIC group, according to our pre-experimental work. Here, we estimate a 9-degree improvement with RIC based on the results of preliminary observations. Using a two-tailed t-test to compare the difference between means and assuming α=0.05 and statistical power of 80%, the estimate sample size is 80 patients (40 each in the RIC and sham-RIC groups). Considering a 20% loss to follow up, we estimate that 100 patients will be required (50 each in the RIC and sham-RIC groups).

6.Trial Design

This study aims to enroll 100 consecutive patients with acute ischemic stroke who receive IVT therapy. The enrolment follows the principles of randomization and parallel control. The patients will be randomly assigned to RIC group or sham-RIC group, maintaining a 1:1 ratio for a study period of 90 days.

Both groups will receive CA assessments at 1-2 and 7-10 days after IVT. The RIC group will undergo RIC at 6 and 18–24 h after IVT, involving pressurization of the unaffected arm with a cuff at a threshold of 200 mmHg (at the same position as the blood pressure measuring cuff) for 5 min, followed by reperfusion for 5 min. This process will be repeated for four cycles, totaling 40 min.

Patients in the sham-RIC group will undergo sham RIC at 6 and 18–24 h after IVT, which includes pressurization of the unaffected arm with a cuff at 60 mmHg (at the same position as the blood pressure measuring cuff) for 5 min, followed by reperfusion for 5 min. This will be repeated for four cycles, totaling 40 min.

Follow-up will continue until 90 days after IVT in both groups to determine the effect of RIC on cerebral hemodynamics in patients after IVT.

7. Randomization

Randomization is established by a researcher who is not involved in the recruitment and assessment of the participants. A random numerical sequence with a 1:1 allocation ratio is computer-generated by an independent biostatistician using SPSS (IBM Corp., Armonk, NY, USA) before starting the study. The randomization code is concealed using sequentially numbered sealed opaque envelopes. After the baseline assessment and obtaining written informed consent, a researcher who is not involved in the data analysis or clinical ratings open the sealed envelope to identify the group to which the participant is allocated (RIC or sham-RIC group) and perform the treatment.

8. The Flow of the Trial

8.1 Visit 1: Screening and Baseline Stage (-4.5-6 h)

The investigator will carefully complete the case screening form, ensuring eligibility per the criteria. If a case does not meet the criteria, the process should be terminated after documenting the reasons. For eligible cases, participants will sign the “Informed Consent Form”, complete the Case Report Form (CRF), and provide the following information:

1) Demographic details: age, sex, nationality, education level, occupation, home address, and contact number, etc.

2) Previous medical and drug treatment history.

a) Previous medical history: history of smoking and alcohol consumption, stroke (if any, specifying the type of stroke), heart disease, hypertension, diabetes, or lipid disorders, as well as

family history of cerebrovascular disease.

b) Concomitant medications: whether the participant took any medication before the onset, such as antiplatelet drugs, anticoagulants, lipid-lowering drugs, antihypertensive drugs, and antidiabetic drugs, etc. The names, durations, and doses of the medications used, as well as the dosage, total amount, and duration of drugs used in the current episode, should be recorded.

3) Present medical history: onset time (last known as normal), premorbid mRS score, clinical presentation, physical examination (height, weight, waist circumference, pre-thrombolysis NIHSS score).

4) Vital signs: heart rate, blood pressure, respiration, and temperature at baseline.

5) Blood sample collection before thrombolysis (optional):

a) Blood samples (two yellow blood collection tube, one purple blood collection tube):

b) Yellow tube: Separate serum within 2 h of blood collection. For short-term storage, place the serum in a freezer at -80°C. For long-term storage, place the serum in liquid nitrogen.

c) Purple tube: Separate plasma and cellular components as soon as possible after blood collection. For short-term storage, place in a -80°C freezer; for long-term storage, place in liquid nitrogen.

6) Results of the first brain computed tomography (CT)/magnetic resonance imaging (MRI), electrocardiogram (ECG), etc.

7) Laboratory tests:

a) Blood routine examination: White Blood Cell Count (WBC), Absolute Neutrophil Count (NEUT#), Absolute Lymphocyte Count (LYM#), Red Blood Cell Count (RBC), Hemoglobin (HGB), Platelet Count (PLT).

b) Routine coagulation examination: Prothrombin Time (PT), Thrombin Time (TT), Activated Partial Thromboplastin Time (APTT), Fibrinogen (FIB), International Normalized Ratio (INR), Prothrombin activity (PTA), Prothrombin time ratio (PTR).

c) Renal function tests (ask about a history of kidney disease; if no relevant disease occurs, results are not required but should be completed within 24 h): Creatinine (Cr) and blood urea nitrogen (BUN)/urea.

d) Liver function tests (ask about a history of liver disease; if no relevant disease occurs, results are not required but should be completed within 24 h): Aspartate transaminase (AST), alanine transaminase (ALT), total bilirubin (T-BiL), direct bilirubin (D-BiL), indirect bilirubin (I-BiL).

e) Fingerstick blood glucose: Glucose (GLU).

f) Other laboratory texts such as glycosylated Hemoglobin (HbA1c), hypersensitive C-reactive Protein (hs-CRP), Homocysteine (Hcy), etc.

8) Fill in the above information into the CRF for Visit 1.

8.2 Visit 2: Treatment Stage (6-24 h)

Collect and record the following information:

1) Concurrent medications within 24 h after IVT, along with vital signs, NIHSS score at 24 h after IVT.

2) Brain CT result (re-examined at 24 h after IVT), whether hemorrhagic transformation.

3) Blood sample collection:

a) Blood samples (two yellow blood collection tube, one purple blood collection tube:):

b) Yellow tube: Separate serum within 2 h of blood collection. For short-term storage, place

the serum in a freezer at -80°C. For long-term storage, place the serum in liquid nitrogen.

c) Purple tube: Separate plasma and cellular components as soon as possible after blood collection. Short-term storage: place in a freezer at -80°C; long-term storage: place in liquid nitrogen.

4) Complete two times of RIC treatment at 6 and 18-24 h after IVT, recording the treatment site, number of cycles, time, and the occurrence of adverse events (AEs).

5) Fill in the above information into the CRF for Visit 2.

During this period, the investigator should closely observe the patient's condition and note any AEs or recurrence/progression of stroke; if any of these occur, detailed records should be made, and a decision on whether to terminate the study should be made based on established standards. Except for death, withdrawal of informed consent, or a request from the investigator administrative department/ethics committee to terminate the study, follow-up must be completed for patients who discontinue the study.

8.3 Visit 3: Follow-up Stage (1-10 d)

1) Concurrent medications within the hospitalization; recording vital signs, NIHSS score of participants at 7 days after IVT or at discharge. If the patient is still in the hospital, record the scores at 7 days; if the patient is discharged before 7 days, record the corresponding scores at the time of discharge; if the patient is discharged after 7 days, record the corresponding scores at 7 days.

2) Patients in both groups undergo CA assessments at 1-2 and 7-10 days after IVT.

3) Blood pressure variability and heart rate variability results are recorded at 1-2 and 7-10 days after IVT.

4) Brain diffusion-weighted magnetic resonance imaging (MRI+DWI) scan results after two times of RIC treatment, recording the infarct location and volume.

5) Discharge diagnosis and medications information.

6) Fill the above information into the CRF for Visit 3.

During this period, the investigator should closely observe the patient's condition and note any AEs or recurrence/progression of stroke; if any of these occur, detailed records should be made, and a decision on whether to terminate the study should be made based on established standards. Except for death, withdrawal of informed consent, or a request from the investigator administrative department/ethics committee to terminate the study, follow-up must be completed for patients who discontinue the study.

8.4 Visit 4: Follow-up Stage (90±3 d)

This trial requires once follow-up visit after discharge, which will be conducted via telephone and video. The follow-up visit is scheduled at 90±3 days after IVT. During follow-up, it is essential to inquire about the occurrence of endpoints, deaths, and AEs. Additionally, details regarding the patient's medication regimen, and mRS score, should be obtained and documented.

Participants have the right to withdraw from the study at any point. Participants are required to complete the study-designed follow-up visit. Except for death, withdrawal of informed consent, or a request from the investigator administrative department/ethics committee to terminate the study, follow-up must be completed for patients who discontinue the study.

9. Combination Medications

This study does not restrict any combination medications, but all combination medications or

alterations in dosing regimens must be documented in the CRF. Each comorbid medication should include details like the drug name, dosage, purpose, and frequency/duration of use for analysis and reporting during the summary.

10. Patient Population

10.1 Inclusion Criteria

1) Age ≥ 18 and < 80 years, both sexes.
2) A clear clinical diagnosis of acute ischemic stroke and treatment with standard rt-PA (0.9 mg/kg) IVT within 4.5 h of stroke onset.
3) Pre-onset modified Rankin Scale (mRS) score ≤1.
4) Baseline National Institute of Health Stroke Scale (NIHSS) score ≥5 and ≤25.
5) Glasgow Coma Scale score ≥8.

10.2 Exclusion Criteria

1) Having received bridging therapy (IVT plus mechanical thrombectomy).
2) Previous history of atrial fibrillation or electrocardiographic evidence of atrial fibrillation.
3) Contraindications to RIC treatment or previous RIC treatment or similar treatment.
4) Pregnancy or breastfeeding.
5) Life expectancy of ≤3 months or inability to complete the study for other reasons.
6) Unwillingness to be followed up or poor treatment compliance or participation in other clinical studies.
7) Had insufficient bilateral temporal bone windows for insonation of the middle cerebral artery.

10.3 Suspension Criteria

Trial suspension means that participants who have been enrolled in the study cannot continue during the study, and treatment prescribed by the protocol is discontinued early, but safety or post-treatment visits can continue. The goal is to protect participants' rights and interests, ensure trial quality, and prevent unnecessary economic losses. Suspension may occur under the following circumstances:

1) Serious safety issues occur during the trial, and the suspension is deemed necessary by the investigator.
2) Trial should be suspended when participants develop unstable vital signs or coma after treatment.
3) The participants experience complications, AEs, or special physiological changes during the trial that may affect their efficacy and safety assessments.
4) Significant trial design defects or major deviations are identified during implementation.
5) Regulatory authorities or ethics committees request study suspension.
6) Other reasons.

10.4 Dropout Criteria

Determination of dropout: Participants who signed an informed consent form and qualified during screening but does not complete the entire clinical trial should be considered dropouts, including voluntary withdrawal, revoked consent, or being lost to follow-up. The investigator should record the reason for dropout in CRF and retain source documents for reference. The dropout rate should not exceed 20%.

11. Adverse Events

11.1 Definition of Adverse Events

Any adverse medical event that occurs from the time of randomization until the last follow-up, whether related to the study treatment, is considered an AE. Following the regulatory requirements, any undesired event that occurs after a participant has signed an informed consent form to participate in a clinical study until its completion or withdrawal from that clinical study is an AE. The investigator should report all AEs directly observed by the physician or spontaneously reported by the participants, using concise medical terminology. AEs are reactions that are directly or indirectly related to the treatment method. In the case of events for which it is difficult to determine the relationship and prevent omission, any AEs should be recorded in a CRF documenting the time of occurrence, severity, duration, measures taken, and regression of the AE, with a separate statement of its possible relevance to the treatment.

11.2 Observation, Assessment, Recording, and Reporting of Adverse Events

Safety assessments will be conducted for all participants who receive treatment at least once. This includes recording the name of the AE, severity, outcome, relationship with treatment, and measures taken. The following should also be recorded: death, recurrent stroke, termination of treatment for any reason, abnormal laboratory test results, changes in vital signs, and changes in physical examination results. The researcher will assess, sign, and date the relationship with the investigational treatment. AEs will be recorded from randomization until the last follow-up. If an AE occurs, follow-up should continue until it disappears, the patient recovers to baseline levels, or becomes clinically insignificant. All AEs during the study will be recorded in the designated CRF AE form.

11.3 Severity Grading Criteria for Adverse Events

AEs will be reported and graded according to the National Cancer Institute's (NCI) Common Terminology Criteria for Adverse Events (CTCAE) version 5.0. Severity is classified as mild, moderate, severe, extremely severe or death and defined as follows:

1) Mild: No or mild symptoms or diseases, only clinically or diagnostically observed, rapid recovery after stopping the study intervention, and no treatment required.

2) Moderate: Causing temporary damage requires minor, local, or non-invasive treatment; easy recovery; and does not require an extended hospital stay.

3) Severe: Causes serious or medically significant but not immediately life-threatening damage requiring extended hospitalization (more than 7 days) or permanent damage to systems/organs.

4) Extremely severe: Life-threatening conditions (with symptoms such as suffocation, shock, or coma) require emergency rescue treatment.

5) Death: Death related to the AE.

11.4 Association between Adverse Events and Study Treatment

Investigators should assess the potential association between AEs and study treatment, as well as concomitant drug use, based on the following 5-level classification criteria:

1) Related: The occurrence of AEs follows a reasonable temporal sequence with the study treatment; AEs match known types of adverse reactions to the suspected study treatment; the severity of AEs improves or disappears after stopping or reducing the study treatment; the AE reappears upon re-

administration of the study treatment and cannot be explained by the participant's disease or concomitant drug use.
2) Probably related: The occurrence of AEs follows a reasonable temporal sequence with the study treatment; AEs match known types of adverse reactions to the suspected study treatment; the severity of AEs can or cannot be improved or resolved after stopping or reducing the study treatment; the AE can or cannot recur upon re-administration of the study treatment; the patient's clinical condition or other treatments (such as concomitant drug use) could also produce an AE.
3) Probably unrelated: The occurrence of AEs follows a reasonable temporal sequence with the study treatment; AEs do not match known types of adverse reactions to the suspected study treatment; the severity of AEs can or cannot be improved or resolved after stopping or reducing the study treatment; the AE can or cannot recur upon re-administration of the study treatment; and the patient's clinical condition or other treatments (such as concomitant drug use) could also produce an AE.
4) Unrelated: The occurrence of AEs does not follow a reasonable temporal sequence with the study treatment; AEs do not match known types of adverse reactions to the suspected study treatment; the severity of AEs did not improve or disappear after stopping or reducing the study treatment; the AE does not occur upon re-administration of the study treatment; the patient's clinical condition or other treatments (such as concomitant drug use) could produce AEs.
5) Unjudged: A judgment cannot be made due to contradictory information or the fact that the information cannot be supplemented and verified.

11.5 Management of Adverse Events

1) When an AE is discovered, researchers should decide whether to suspend observation based on the patient's condition. In the case of a serious AE, the clinical research unit must take immediate measures to protect the safety of the participants. All AEs will be tracked and investigated in detail, and the process and results of handling will be recorded until proper resolution is achieved or conditions stabilized. If the laboratory test results are abnormal, follow-up should be continued until the results are normal. Follow-up methods can be selected based on the severity of the AEs, including hospitalizations, outpatient visits, home visits, telephone calls, and correspondence.
2) Emergencies, including serious (particularly life-threatening) AEs, should be reported to the provincial, autonomous region, and municipal adverse reaction monitoring professional institutions using the fastest communication method.

11.6 Serious Adverse Events

1) Serious AEs are defined as AEs that lead to the following results:

a) Death

b) Life-threatening (refers to a situation where the subject is at risk of death when an AE occurs, not events that might lead to death under hypothetical worsening conditions)

c) Requires hospitalization or prolongation of hospitalization

d) Causes persistent or significant disability or dysfunction

e) Events that the investigator judges as medical events can also be judged as serious AEs

2) Suppose a subject experiences a serious AE during the trial, regardless of whether it is related to the study treatment. In that case, the investigator will immediately implement appropriate treatment

measures to ensure the safety of the subject. The event must be reported within 24 hours to the principal research center, local ethics committee. Simultaneously, the investigator must complete a serious AE.

3) Serious AEs that are not resolved when the study ends or the subject withdraws prematurely must be followed up until any of the following conditions are met:

a) The event disappears or is resolved;

b) The event stabilizes;

c) The event returns to the baseline level (if baseline values are available);

d) The event alleviates to a clinically insignificant level;

e) The event can be attributed to a drug other than the study treatment, factors unrelated to the study, or when more information is unlikely to be obtained (the patient or caregiver refuses to provide more information, or there is evidence that after the best effort has been made, the patient is still lost to follow-up).

12. Statistical Analysis

Intention-to-treat (ITT) population: According to the basic principle of intention-to-treat (ITT), all randomly assigned patients will be included in the ITT population. The ITT population will be the main analysis population for the efficacy endpoint, and subjects will be analysed according to the treatment group to which they are assigned at randomization.

Per-Protocol (PP) population: The PP population is a subset of ITT population, including all randomized subjects who have been treated in the study without major protocol deviations that may significantly impact the interpretation of efficacy results.

Safety population: The safety population includes all patients who received at least one RIC or sham-RIC treatment.

13. Appendix

13.1 Appendix 1: Study Flow Chart

<table>
<tr><th>Stage Item</th><th>Screening</th><th>Treatment Stage</th><th colspan="2">Follow-up</th></tr>
<tr><th>Visit</th><th>Visit 1</th><th>Visit 2</th><th>Visit 3</th><th>Visit 4</th></tr>
<tr><th>Time</th><th>-4.5 h-6 h</th><th>6 h-24 h</th><th>1 d-10 d</th><th>90±3 d</th></tr>
<tr><td>Signed informed consent</td><td>×</td><td></td><td></td><td></td></tr>
<tr><td>Demographic characteristics</td><td>×</td><td></td><td></td><td></td></tr>
<tr><td>Medical history</td><td>×</td><td></td><td></td><td></td></tr>
<tr><td>Combination medications</td><td>×</td><td>×</td><td>×</td><td>×</td></tr>
<tr><td>Present medical history</td><td>×</td><td></td><td></td><td></td></tr>
<tr><td>Vital signs</td><td>×</td><td>×</td><td>×</td><td></td></tr>
<tr><td>NIHSS score</td><td>×</td><td>×</td><td>×</td><td></td></tr>
<tr><td>mRS score</td><td>×</td><td></td><td></td><td>×</td></tr>
<tr><td>Laboratory tests</td><td colspan="3">×</td><td></td></tr>
<tr><td>ECG</td><td>×</td><td></td><td></td><td></td></tr>
<tr><td>Brain CT scan</td><td>×</td><td>×</td><td></td><td></td></tr>
<tr><td>Brain MRI+DWI</td><td></td><td></td><td>×</td><td></td></tr>
<tr><td>Blood specimen</td><td>× (optional)</td><td>×</td><td></td><td></td></tr>
<tr><td>RIC</td><td></td><td>×</td><td></td><td></td></tr>
<tr><td>CA</td><td></td><td></td><td>×</td><td></td></tr>
<tr><td>Discharge diagnosis</td><td></td><td></td><td>×</td><td></td></tr>
<tr><td>Adverse events</td><td>×</td><td>×</td><td>×</td><td>×</td></tr>
</table>

0 h: Immediately after receiving intravenous thrombolysis.

Laboratory tests include routine blood tests, coagulation routine tests, kidney function, liver function, HbA1c, hs-CRP, hcy, fasting blood glucose, NT-proBNP, uric acid, erythrocyte sedimentation rate, serum cysteine c, blood Lipids, serum ion, etc.

Abbreviation: RIC, remote ischemic conditioning; CA, cerebral autoregulation; NIHSS, National Institute of Health Stroke Scale; mRS: modified Rankin Scale; ECG, Electrocardiograph; CT, computed tomography; MRI+DWI, diffusion-weighted magnetic resonance imaging.

13.2 Appendix 2: Modified Rankin Scale

The mRS is used to measure the patient's functional recovery post-stroke. Please note that only symptoms that occurred after the stroke are considered. A patient is considered able to walk independently if they could walk without external help despite the use of assistive devices.

In cases where both levels appear applicable and further questioning would not lead to a correct choice, a more severe level should be chosen.

Grade	Description
0	No symptoms
1	Symptoms without any incapacity (able to perform all usual activities)
2	Mild incapacity (unable to perform all usual activities but able to look after his/her affairs alone)
3	Moderate incapacity (requires assistance but walks alone)
4	Severe incapacity (requires assistance for walking and physical body needs)
5	Severe incapacity (bedbound, incontinent, permanent surveillance required)
6	Death

13.3 Appendix 3: National Institutes of Health Stroke Scale

Check	Scores	Point
**1a. Level of Consciousness (0-3):** Verbal or painful stimulation of the patient (left to the choice of the examiner)	0 = Aware, responds briskly.<br>1 = Responds to minor stimuli.<br>2 = Responds to repeated or painful stimuli.<br>3 = Stereotypic or flaccid response.	
**1b. Consciousness level (0-2):**<br>- "How old are you?"<br>- "What month is this?"	0 = Correct answer to the 2 questions.<br>1 = One single correct answer or intubated, or severe dysarthria or language barrier.<br>2 = No correct response or aphasic.	
**1c. Consciousness-commands (0-2):**<br>- "Open and close your eyes"<br>- "Close and open your hand"<br>To be imitated if the command is not followed	0 = Performs both tasks correctly.<br>1 = Performs one task correctly.<br>2 = Performs neither task correctly.	
**2. Horizontal oculomotilities (0-2):** Test voluntary and reflex horizontal movements without a heat test (finger following): "Follow my finger".	0 = Normal oculomotility.<br>1 = Conjugate deviation of the eyes which may be reduced by voluntary or reflex activity or isolated damage to a cranial nerve.<br>2 = Complete paralysis of the side.	
**3. Visual field (0-3):**<br>Test the visual field by quadrants (upper and lower) using finger counting or if necessary the menace blink reflex.	0 = Normal visual field.<br>1 = Partial HLH or loss of vision.<br>2 = Complete HLH.<br>3 = Dual HLH or cortical blindness.	
**4. Facial paralysis (0-3):**<br>"Show me your teeth, raise your eyebrows and close your eyes"<br>*To be imitated if command not performed or Pierre Marie and Foix manoeuvre.*	0 = Normal.<br>1 = Slight central facial paralysis (FP).<br>2 = Clear central PF (total inferior).<br>3 = Dual PF or total PF.	
**6. Upper limb motility:**<br>6.1 "Stretch out your arm and left hand" (0-4).<br>6.2 "Stretch out your arm and right hand" (0-4) for 10 seconds.	0 = Normal.<br>1 = Resists a weight (arm falls within 10 sec).<br>2 = Does not resist (arm touches the bed before 10 sec).<br>3 = Does not lift limb (contraction without movement).<br>4 = No movement.	5a Left upper extremity<br>5b Right upper limb

**7. Lower limb motility:**<br>6.1 "Stretch out your left leg" (30°) (0-4)<br>6.2 "Stretch out your right leg" (0-4) for 5 seconds	0 = Normal.<br>1 = Resists a weight (arm falls within 5 sec).<br>2 = Does not resist (arm touches the bed before 5 sec).<br>3 = Does not lift limb (contraction without movement).<br>4 = No movement.	6a Left lower extremity<br>6b Right lower extremity
**7. Limb ataxia (0-2):**<br>"Place your index finger on your nose" "Place your heel on the opposite knee" *Bilateral manoeuvre*	0 = Normal or impossible because of paralysis or aphasia.<br>1 = Ataxia of one limb.<br>2 = Ataxia of 2 limbs.<br>9 = Amputation or joint block.	
**8. Sensitivity (0-2):**<br>Examine sensation to pin prick or withdrawal following nociceptive stimulation if confused or aphasic (arm, leg, face, trunk, bilaterally).	0 = Normal.<br>1 = Hypoesthesia or aphasia or stupor.<br>2 = Severe to total deficit.	
**9. Language (0-3):**<br>"Describe the following scene"<br>"Tell me the name of these objects" "Read these phrases"<br>If visual disturbance, identify the objects in a hand and have the words repeated.<br>*Assess writing in an intubated patient*	0 = Normal.<br>1 = Aphasic but communicates.<br>2 = Quasi-impossible communication.<br>3 Total aphasia, mutism or coma.	
**10. Dysarthria (0-2):**<br>"Repeat the following words" (cf. text 2 p 3)<br>***including** in aphasic patients.*	0 = Normal articulation.<br>1 = Comprehensible.<br>2 = Incomprehensible, anarthria or mutism.<br>9 = Intubation or mechanical obstruction.	
**11. Extinction or negligence:**<br>Test bilateral simultaneous sensitivity<br>Test perception in both temporal visual fields simultaneously Investigate for anosognosia and visuospatial neglect.	0 = No extinction or complete HLH (if sensory extinction) and vice versa or aphasia or gives the impression of understanding.<br>1 = Extinction of one modality.<br>2 = Extinction of several modalities or visuospatial neglect or anosognosia.	

14. Reference

1. Roth GA, Mensah GA, Johnson CO, et al. Global Burden of Cardiovascular Diseases and Risk Factors, 1990-2019: Update From the GBD 2019 Study. J Am Coll Cardiol. 2020;76(25):2982-3021. doi:10.1016/ j. jacc.2020.11.010.

2. Powers WJ, Rabinstein AA, Ackerson T, et al. Guidelines for the Early Management of Patients With Acute Ischemic Stroke: 2019 Update to the 2018 Guidelines for the Early Management of Acute Ischemic Stroke: A Guideline for Healthcare Professionals From the American Heart Association/American Stroke Association. Stroke. 2019;50(12): e344-e418. doi:10.1161/STR.0000000000000211.

3. Berge E, Whiteley W, Audebert H, et al. European Stroke Organisation (ESO) guidelines on intravenous thrombolysis for acute ischaemic stroke. Eur Stroke J. 2021;6(1): I-LXII. doi:10.1177/2396987321989865.

4. Chinese Society of Neurology; Chinese Stroke Society. Chinese Guideline for Endovascular Treatment of Acute Ischemic Stroke 2018. Chin J Neurol. 2018; 51:666-682. doi:10.3969/ j. issn.1673-5765.2018.07.014.

5. Hankey GJ. Stroke. Lancet. 2017;389(10069):641-654. doi:10.1016/S0140-6736(16)30962-X.

6. Emberson J, Lees KR, Lyden P, et al. Effect of treatment delay, age, and stroke severity on the effects of intravenous thrombolysis with alteplase for acute ischaemic stroke: a meta-analysis of individual patient data from randomised trials. Lancet. 2014;384(9958):1929-1935. doi:10.1016/S0140-6736(14)60584-5.

7. MacGregor DG, Carswell HV, Graham DI, et al. Impaired cerebral autoregulation 24 h after induction of transient unilateral focal ischaemia in the rat. Eur J Neurosci. 2000;12(1):58-66.

8. Fan JL, O'Donnell T, Lanford J, et al. Dietary nitrate reduces blood pressure and cerebral artery velocity fluctuations and improves cerebral autoregulation in transient ischemic attack patients. J Appl Physiol (1985). 2020;129(3):547-57.

9. Aaslid R LK, Sorteberg W, Nornes H. Cerebral autoregulation dynamics in humans. Stroke. 1989;20(1):45-52.

10. Murry CE, Jennings RB, Reimer KA. Preconditioning with ischemia: a delay of lethal cell injury in ischemic myocardium. Circulation. 1986;74(5):1124-1136. doi:10.1161/01. cir. 74.5.1124.

11. Weir P, Maguire R, O'Sullivan SE, England TJ. A meta-analysis of remote ischaemic conditioning in experimental stroke. J Cereb Blood Flow Metab. 2021;41(1):3-13. doi: 10.1177/0271678X20924077.

12. Guo ZN, Guo WT, Liu J, et al. Changes in cerebral autoregulation and blood biomarkers after remote ischemic preconditioning. Neurology. 2019;93(1): e8-e19.

13. Qu Y, Zhang P, He QY, et al. The Impact of Serial Remote Ischemic Conditioning on Dynamic Cerebral Autoregulation and Brain Injury Related Biomarkers. Front Physiol. 2022; 13:835173.

14. Hacke W, Kaste M, Fieschi C, et al. Intravenous thrombolysis with recombinant tissue plasminogen activator for acute hemispheric stroke. The European Cooperative Acute Stroke Study (ECASS). JAMA. 1995 Oct 4;274(13):1017-25.

Summary of changes

(From Version 1.0, December 26, 2022, to Version 2.0, March 05, 2023)

Below is the table of changes.

**Sections**	**Protocol Version 1.0 Change From:**	**Protocol Version 2.0 Change To:**	**Rational**
**1. Study Synopsis- Trial Design**	This study aims to enroll 100 consecutive patients with acute ischemic stroke who receive intravenous thrombolytic (IVT) therapy. The enrolment follows the principles of randomization and parallel control. The patients will be randomly assigned to remote ischemic conditioning (RIC) group or sham-RIC group, maintaining a 1:1 ratio for a study period of 90 days.<br>Both groups will receive cerebral autoregulation (CA) assessments at 1-2 and 7-10 days after IVT. The RIC group will undergo RIC at 6 and 18–24 h after IVT, involving pressurization of the unaffected arm with a cuff at a threshold of 200 mmHg (at the same position as the blood pressure measuring cuff) for 5 min, followed by reperfusion for 5 min. This process will be repeated for four cycles, totaling 40 min.<br>Patients in the sham-RIC group will undergo sham RIC at 6 and 18–24 h after IVT, which includes pressurization of the unaffected arm with a cuff at 60 mmHg (at the same position as the	This study aims to enroll 100 consecutive patients with acute ischemic stroke who receive intravenous thrombolytic (IVT) therapy. The enrolment follows the principles of randomization and parallel control. The patients will be randomly assigned to remote ischemic conditioning (RIC) group or sham-RIC group, maintaining a 1:1 ratio for a study period of 90 days.<br>Both groups will receive cerebral autoregulation (CA) assessments at 2 and 7 days after IVT. The RIC group will undergo RIC at 6 and 18–24 h after IVT, involving pressurization of the unaffected arm with a cuff at a threshold of 200 mmHg (at the same position as the blood pressure measuring cuff) for 5 min, followed by reperfusion for 5 min. This process will be repeated for four cycles, totaling 40 min.<br>Patients in the sham-RIC group will undergo sham RIC at 6 and 18–24 h after IVT, which includes pressurization of the unaffected arm with	The time window of the study endpoint was specified by modifying 1-2 days and 7-10 days after IVT to 2 days and 7 days after IVT.

	blood pressure measuring cuff) for 5 min, followed by reperfusion for 5 min. This will be repeated for four cycles, totaling 40 min.<br>Follow-up will continue until 90 days after IVT in both groups to determine the effect of RIC on cerebral hemodynamics in patients after IVT.	a cuff at 60 mmHg (at the same position as the blood pressure measuring cuff) for 5 min, followed by reperfusion for 5 min. This will be repeated for four cycles, totaling 40 min.<br>Follow-up will continue until 90 days after IVT in both groups to determine the effect of RIC on cerebral hemodynamics in patients after IVT.	
**1. Study Synopsis- Outcomes**	**Primary Efficacy Outcome:**<br>The primary endpoint is phase difference (PD, the main parameter of CA) on the affected side at 1-2 days after IVT.<br><br>**Secondary Efficacy Outcomes:**<br>1) PD on the unaffected side at 1-2 days after IVT.<br>2) Gain (the parameter of CA) on the affected side at 1-2 days after IVT.<br>3) Gain on the unaffected side at 1-2 days after IVT.<br>4) PD on the affected side at 7-10 days after IVT.<br>5) PD on the unaffected side at 7-10 days after IVT.<br>6) Gain on the affected side at 7-10 days after IVT.<br>7) Gain on the unaffected side at 7-10 days after IVT.<br>8) NIHSS score at 24 h and 7 days after IVT.<br>9) Proportion of patients with favorable outcomes	**Primary Efficacy Outcome:**<br>The primary endpoint is phase difference (PD, the main parameter of CA) on the affected side at 2 days after IVT.<br><br>**Secondary Efficacy Outcomes:**<br>1) PD on the unaffected side at 2 days after IVT.<br>2) Gain (the parameter of CA) on the affected side at 2 days after IVT.<br>3) Gain on the unaffected side at 2 days after IVT.<br>4) PD on the affected side at 7 days after IVT.<br>5) PD on the unaffected side at 7 days after IVT.<br>6) Gain on the affected side at 7 days after IVT.<br>7) Gain on the unaffected side at 7 days after IVT.<br>8) NIHSS score at 24 h and 7 days after IVT.<br>9) Proportion of patients with favorable outcomes (mRS score≤ 2) at 90 days.<br>10) Final infarct volume.<br>11) Incidence of hemorrhagic transformation	The time window of the study endpoint was specified by modifying 1-2 days and 7-10 days after IVT to 2 days and 7 days after IVT.

	(mRS score≤ 2) at 90 days.<br>10) Final infarct volume.<br>11) Incidence of hemorrhagic transformation within 24 hours.<br>12) Serum levels of brain-injury biomarkers at 24 h after IVT.<br><br>**Safety Outcomes:**<br>1) Mortality within 90 days.<br>2) All adverse events within 90 days.	within 24 hours.<br>12) Serum levels of brain-injury biomarkers at 24 h after IVT.<br><br>**Safety Outcomes:**<br>1) Mortality within 90 days.<br>2) All adverse events within 90 days.	
**4.2 Study Outcomes**	**4.2.1 Primary Efficacy Outcome**<br>The primary endpoint is PD on the affected side at 1-2 days after IVT.<br><br>**4.2.2 Secondary Efficacy Outcomes**<br>1) PD on the unaffected side at 1-2 days after IVT.<br>2) Gain on the affected side at 1-2 days after IVT.<br>3) Gain on the unaffected side at 1-2 days after IVT.<br>4) PD on the affected side at 7-10 days after IVT.<br>5) PD on the unaffected side at 7-10 days after IVT.<br>6) Gain on the affected side at 7-10 days after	**4.2.1 Primary Efficacy Outcome**<br>The primary endpoint is PD on the affected side at 2 days after IVT.<br><br>**4.2.2 Secondary Efficacy Outcomes**<br>1) PD on the unaffected side at 2 days after IVT.<br>2) Gain on the affected side at 2 days after IVT.<br>3) Gain on the unaffected side at 2 days after IVT.<br>4) PD on the affected side at 7 days after IVT.<br>5) PD on the unaffected side at 7 days after IVT.<br>6) Gain on the affected side at 7 days after	The time window of the study endpoint was specified by modifying 1-2 days and 7-10 days after IVT to 2 days and 7 days after IVT.

	IVT.<br>7) Gain on the unaffected side at 7-10 days after IVT.<br>8) NIHSS score at 24 h and 7 days after IVT.<br>9) Proportion of patients with favorable outcomes (mRS score≤ 2) at 90 days.<br>10) Final infarct volume.<br>11) Incidence of hemorrhagic transformation within 24 hours.<br>12) Serum levels of brain-injury biomarkers at 24 h after IVT.<br><br>**4.2.3 Safety Outcomes**<br>1) Mortality within 90 days.<br>2) All adverse events within 90 days.	IVT.<br>7) Gain on the unaffected side at 7 days after IVT.<br>8) NIHSS score at 24 h and 7 days after IVT.<br>9) Proportion of patients with favorable outcomes (mRS score≤ 2) at 90 days.<br>10) Final infarct volume.<br>11) Incidence of hemorrhagic transformation within 24 hours.<br>12) Serum levels of brain-injury biomarkers at 24 h after IVT.<br><br>**4.2.3 Safety Outcomes**<br>1) Mortality within 90 days.<br>2) All adverse events within 90 days.	
**5. Sample Size**	We estimate that the PD on the affected sides at 1-2 days after IVT will be 30.12° in the sham-RIC group, according to our pre-experimental work. Here, we estimate a 9-degree improvement with RIC based on the results of preliminary observations. Using a two-tailed t-test to compare the difference between means and assuming α=0.05 and statistical power of 80%, the estimate sample size is 80 patients (40 each in the RIC and sham-RIC groups). Considering a 20% loss to follow up, we estimate that 100 patients will be	We estimate that the PD on the affected sides at 2 days after IVT will be 30.12° in the sham-RIC group, according to our pre-experimental work. Here, we estimate a 9-degree improvement with RIC based on the results of preliminary observations. Using a two-tailed t-test to compare the difference between means and assuming α=0.05 and statistical power of 80%, the estimate sample size is 80 patients (40 each in the RIC and sham-RIC groups). Considering a 20% loss to follow up, we estimate that 100 patients will be	The time window of the study endpoint was specified by modifying 1-2 days and 7-10 days after IVT to 2 days and 7 days after IVT.

	required (50 each in the RIC and sham-RIC groups).	required (50 each in the RIC and sham-RIC groups).	
**6.Trial Design**	This study aims to enroll 100 consecutive patients with acute ischemic stroke who receive IVT therapy. The enrolment follows the principles of randomization and parallel control. The patients will be randomly assigned to RIC group or sham-RIC group, maintaining a 1:1 ratio for a study period of 90 days.<br>Both groups will receive CA assessments at 1-2 and 7-10 days after IVT. The RIC group will undergo RIC at 6 and 18–24 h after IVT, involving pressurization of the unaffected arm with a cuff at a threshold of 200 mmHg (at the same position as the blood pressure measuring cuff) for 5 min, followed by reperfusion for 5 min. This process will be repeated for four cycles, totaling 40 min.<br>Patients in the sham-RIC group will undergo sham RIC at 6 and 18–24 h after IVT, which includes pressurization of the unaffected arm with a cuff at 60 mmHg (at the same position as the blood pressure measuring cuff) for 5 min, followed by reperfusion for 5 min. This will be repeated for four cycles, totaling 40 min.<br>Follow-up will continue until 90 days after IVT in both groups to determine the effect of RIC	This study aims to enroll 100 consecutive patients with acute ischemic stroke who receive IVT therapy. The enrolment follows the principles of randomization and parallel control. The patients will be randomly assigned to RIC group or sham-RIC group, maintaining a 1:1 ratio for a study period of 90 days.<br>Both groups will receive CA assessments at 2 and 7 days after IVT. The RIC group will undergo RIC at 6 and 18–24 h after IVT, involving pressurization of the unaffected arm with a cuff at a threshold of 200 mmHg (at the same position as the blood pressure measuring cuff) for 5 min, followed by reperfusion for 5 min. This process will be repeated for four cycles, totaling 40 min.<br>Patients in the sham-RIC group will undergo sham RIC at 6 and 18–24 h after IVT, which includes pressurization of the unaffected arm with a cuff at 60 mmHg (at the same position as the blood pressure measuring cuff) for 5 min, followed by reperfusion for 5 min. This will be repeated for four cycles, totaling 40 min.<br>Follow-up will continue until 90 days after	The time window of the study endpoint was specified by modifying 1-2 days and 7-10 days after IVT to 2 days and 7 days after IVT.

	on cerebral hemodynamics in patients after IVT.	IVT in both groups to determine the effect of RIC on cerebral hemodynamics in patients after IVT.	
**8.3 Visit 3: Follow-up Stage**	**8.3 Visit 3: Follow-up Stage (1-10 d)**<br>1) Concurrent medications within the hospitalization; recording vital signs, NIHSS score of participants at 7 days after IVT or at discharge. If the patient is still in the hospital, record the scores at 7 days; if the patient is discharged before 7 days, record the corresponding scores at the time of discharge; if the patient is discharged after 7 days, record the corresponding scores at 7 days.<br>2) Patients in both groups undergo CA assessments at 1-2 and 7-10 days after IVT.<br>3) Blood pressure variability and heart rate variability results are recorded at 1-2 and 7-10 days after IVT.<br>4) Brain diffusion-weighted magnetic resonance imaging (MRI+DWI) scan results after two times of RIC treatment, recording the infarct location and volume.<br>5) Discharge diagnosis and medications information.<br>6) Fill the above information into the CRF for Visit 3.<br>During this period, the investigator should closely	**8.3 Visit 3: Follow-up Stage (1-7 d)**<br>1) Concurrent medications within the hospitalization; recording vital signs, NIHSS score of participants at 7 days after IVT or at discharge. If the patient is still in the hospital, record the scores at 7 days; if the patient is discharged before 7 days, record the corresponding scores at the time of discharge; if the patient is discharged after 7 days, record the corresponding scores at 7 days.<br>2) Patients in both groups undergo CA assessments at 2 and 7 days after IVT.<br>3) Blood pressure variability and heart rate variability results are recorded at 2 and 7 days after IVT.<br>4) Brain diffusion-weighted magnetic resonance imaging (MRI+DWI) scan results after two times of RIC treatment, recording the infarct location and volume.<br>5) Discharge diagnosis and medications information.<br>6) Fill the above information into the CRF for Visit 3.<br>During this period, the investigator should closely	The time window of the study endpoint was specified by modifying 1-2 days and 7-10 days after IVT to 2 days and 7 days after IVT. The follow-up period changed from 1-10 days to 1-7 days.

	observe the patient's condition and note any AEs or recurrence/progression of stroke; if any of these occur, detailed records should be made, and a decision on whether to terminate the study should be made based on established standards. Except for death, withdrawal of informed consent, or a request from the investigator administrative department/ethics committee to terminate the study, follow-up must be completed for patients who discontinue the study.	observe the patient's condition and note any AEs or recurrence/progression of stroke; if any of these occur, detailed records should be made, and a decision on whether to terminate the study should be made based on established standards. Except for death, withdrawal of informed consent, or a request from the investigator administrative department/ethics committee to terminate the study, follow-up must be completed for patients who discontinue the study.	

Statistical Analysis Plan

Effect of Remote Ischemic Conditioning on Cerebral Hemodynamics in Patients After Intravenous Thrombolysis

(RICCH-IVT)

Version 2.0, 05 March 2023

Principal Investigator

Yi Yang, MD, PhD

The First Hospital of Jilin University, Jilin University, Changchun, China

ClinicalTrials.gov registration number: NCT05598658

Contents

LIST OF ABBREVIATIONS **60**

1. INTRODUCTION **61**

1.1 Background to this study 61

1.2 Purpose of the statistical analysis plan 61

2. STUDY OBJECTIVES AND OUTCOMES **61**

2.1 Study Objectives 62

2.2 Study Outcomes 62

2.2.1 Primary efficacy outcome 62

2.2.2 Secondary efficacy outcomes 62

2.2.3 Safety outcomes 62

2.2.4 Case definitions 62

3. STUDY METHODS **63**

3.1 Overall study design 63

3.2 Selection of study population 63

3.2.1 Inclusion criteria 63

3.2.2 Exclusion criteria 63

3.3 Treatments 63

3.4 Method of treatment assignment and randomization 63

3.5 Sample size 64

4. ANALYSIS POPULATION **64**

4.1 Intention-to-Treated (ITT) population 64

4.2 Per-protocol (PP) population 64

4.3 Safety population 64

5. GENERAL ISSUES FOR STATISTICAL ANALYSES **64**

5.1 Analysis software 64

5.2 Presentation of results 64

5.3 Methods for withdrawals 65

5.4 Methods for missing data 65

5.4.1 Baseline covariates 65

5.4.2 Efficacy outcomes 65

5.5 Further exploratory analyses 65

6. STATISTICAL ANALYSES **65**

6.1 Demographics and baseline characteristics..65
6.2 Primary efficacy analyses 65
6.2.1 Crude analysis 66
6.2.2 Covariate adjusted analysis 66
6.3 Secondary efficacy outcomes analyses 66
6.3.1 Analysis of binary outcomes..66
6.3.2 Analysis of continuous outcomes...66
6.4 Safety analyses...67
7. REFERENCE **67**

LIST OF ABBREVIATIONS

Abbreviations	Definitions
AE	Adverse Event
AIS	Acute Ischemic Stroke
CI	Confidence Interval
FCS	Fully Conditional Method
ICH	Intracranial Hemorrhage
ITT	Intention-to-Treat
IVT	Intravenous Thrombolysis
MAR	Missing-at-Random
mRS	modified Rankin Scale
NIHSS	National Institute of Health Stroke Scale
NRS	Numerical Rating Scale
PP	Per-protocol
RIC	Remote Ischemic Conditioning
OR	Odds Ratio
SAE	Serious Adverse Event
SAP	Statistical Analysis Plan
SD	Standard Deviation
PD	Phase difference

1. INTRODUCTION

1.1 Background to this study

Stroke is a leading cause of global mortality and disability[1]. Intravenous thrombolysis (IVT) within 4.5 h of stroke onset is recognized as one of the most effective treatments for acute ischemic stroke (AIS)[2-4]. Even so, about 2/3 patients still do not benefit from alteplase intravenous thrombolysis[5-6]. Therefore, how to increase the benefit of IVT, improve the success rate of acute ischemic stroke patients and improve the quality of life of patients have become difficult problems for scientists in various countries.

Cerebral hemodynamics is closely related to the prognosis of ischemic stroke patients. As an important index of cerebral hemodynamics, cerebral autoregulation (CA) refers to a complex process in which intracranial arterioles maintain relatively stable cerebral blood by contraction or relaxation when systemic arterial blood pressure changes, causing changes in intracranial pressure, which can avoid hyperperfusion or hypoperfusion of brain tissue[7-9]. Our previous studies have shown that CA impairment is closely associated with 3-month prognosis in patients with ischemic stroke, and early CA decline is an independent risk factor for poor prognosis.

Remote ischemic conditioning (RIC) is a treatment method that gives intermittent non-lethal reversible ischemic stimulation to a remote vascular bed, organ or tissue to activate endogenous protective mechanisms to protect important ischemic organs[10]. The underlying neuroprotective mechanism triggered by RIC induces gene expression, alters pathways, promotes neurogenesis and blood vessel development, reduces oxidative stress and neuronal apoptosis, and inhibits proinflammatory signals in the ischemic brain[11]. In 2019, our team studied the effects of RIC on CA and blood biomarkers in healthy adults. The results showed that the ability of dynamic CA in healthy adults continued to increase within 6 hours to at least 24 hours after RIC[12]; In addition, at least 2 neuroprotective factors and 4 inflammation-related biological markers in blood changed rapidly within a short time after ischemia, thus providing new evidence for RIPC to induce neuroprotection and improve cerebral vascular function. Recently, our team investigated the effects of a series of RIC on dynamic CA and brain damage-related biomarkers, and showed that after four RIC sessions, the CA was significantly higher than baseline in healthy adults, after 7-day RIC, the CA increase lasts for at least 28 days[13]. At the same time, there were no differences in different serum biomarkers associated with brain damage before and after RIC. The study provides new support for the safety and efficacy of RIC.

However, there is still a lack of studies on the relationship between RIC and cerebral hemodynamics in patients with IVT. Based on the above background, our team designed this study to explore the effect of RIC on cerebral hemodynamics in patients with IVT.

1.2 Purpose of the statistical analysis plan

The purpose of this statistical analysis plan (SAP) is to outline the planned analyses to be completed to support the completion of the clinical study report. The planned analyses identified in this SAP will be included in regulatory submissions and/or future manuscripts. In addition, exploratory analyses not necessarily identified in this SAP may be conducted to support the clinical development plan. Any post-hoc, or unplanned, analyses outside of this SAP will be clearly identified in the respective clinical study report.

2. STUDY OBJECTIVES AND OUTCOMES

2.1 Study objectives

The primary objectives of the study is to determine the effect of RIC on cerebral hemodynamics in patients after IVT.

2.2 Study outcomes

2.2.1 Primary efficacy outcome

The primary endpoint is phase difference (PD, the main parameter of CA) on the affected side at 2 days after IVT.

2.2.2 Secondary efficacy outcomes

The secondary efficacy outcomes for this study are as follows:

1) PD on the unaffected side at 2 days after IVT.
2) Gain (the parameter of CA) on the affected side at 2 days after IVT.
3) Gain on the unaffected side at 2 days after IVT.
4) PD on the affected side at 7 days after IVT.
5) PD on the unaffected side at 7 days after IVT.
6) Gain on the affected side at 7 days after IVT.
7) Gain on the unaffected side at 7 days after IVT.
8) NIHSS score at 24 h and 7 days after IVT.
9) Proportion of patients with favorable outcomes (mRS score≤ 2) at 90 days.
10) Final infarct volume.
11) Incidence of hemorrhagic transformation within 24 hours.
12) Serum levels of brain-injury biomarkers at 24 h after IVT.

2.2.3 Safety outcomes

1) Mortality within 90 days.
2) All adverse events within 90 days.

2.2.4 Case definitions

1) Hemorrhagic transformation:

Hemorrhagic transformation is determined based on the computer tomography scan conducted 24 hours after IVT, using the European Cooperative Acute Stroke Study (ECASS) criteria[14].

2) Death:

All deaths during the study period will be recorded. The cause of each death will be determined based on clinical assessments carried out by the physicians involved in the study.

3) CA assessments:

Before the examination, all patients are instructed to relax in the supine position for 10 min in a dedicated quiet examination room with a controlled temperature ranging from 20°C to 24°C. The bilateral cerebral blood flow velocity (CBFV) in the middle cerebral artery is measured using transcranial Doppler (MultiDop X4; DWL, Sipplingen, Germany) sonography. Two 2-MHz probes are fixed with a customized head frame in the bilateral temporal bone window at a depth of 45–60 mm. Simultaneously, arterial blood pressure in the digital artery (measured using a servo-controlled plethysmograph [Finometer Model 1; FMS, Amsterdam, the Netherlands]) is continuously recorded. The end-tidal CO2 is measured using a capnograph (MultiDop X4; DWL) with a nasal cannula. The

real-time recordings last 10 min and are stored for further analysis.

3. STUDY METHODS

3.1 Overall study design

The RICCH-IVT study is a single-center, single-blinded, endpoint-blinded, randomized controlled clinical trial in patients with AIS who undergo IVT within 4.5 hours of onset. Patients will be randomized 1:1 to either RIC group or control (sham-RIC) group. It is planned to recruit 100 patients from the First Hospital of Jilin University.

3.2 Selection of study population

3.2.1 Inclusion criteria

1) Age ≥ 18 and < 80 years, both sexes.
2) A clear clinical diagnosis of acute ischemic stroke and treatment with standard rt-PA (0.9 mg/kg) IVT within 4.5 h of stroke onset.
3) Pre-onset modified Rankin Scale (mRS) score ≤1.
4) Baseline National Institute of Health Stroke Scale (NIHSS) score ≥5 and ≤25.
5) Glasgow Coma Scale score ≥8.

3.2.2 Exclusion Criteria

1) Having received bridging therapy (IVT plus mechanical thrombectomy).
2) Previous history of atrial fibrillation or electrocardiographic evidence of atrial fibrillation.
3) Contraindications to RIC treatment or previous RIC treatment or similar treatment.
4) Pregnancy or breastfeeding.
5) Life expectancy of ≤3 months or inability to complete the study for other reasons.
6) Unwillingness to be followed up or poor treatment compliance or participation in other clinical studies.
7) Had insufficient bilateral temporal bone windows for insonation of the middle cerebral artery.

3.3 Treatments

The two trial arms of this study are as follows:
RIC group: RIC, which is given at 6 and 18–24 h after IVT.
Control (sham-RIC) group: sham-RIC, which is given at 6 and 18–24 h after IVT.

Patients in the two groups will receive standard medical care. After randomization, participants in both groups will undergo RIC treatment at 6 h and 18-24 h after IVT using an automated device (BB-RIC-D5/LAPUL Medical Devices Co., Ltd, Beijing, China) placed on the upper arm of the unaffected side. Each RIC will consist of four cycles of arm ischemia with cuffs inflated to a pressure of 200 mmHg (RIC group) or 60 mmHg (control group) for 5 min, followed by reperfusion for another 5 min, for a total procedure time of 40 min. Hospital-based nurses will perform the RIC procedure.

3.4 Method of treatment assignment and randomization

Randomization is established by a researcher who is not involved in the recruitment and assessment of the participants. A random numerical sequence with a 1:1 allocation ratio is computer-generated by an independent biostatistician using SPSS (IBM Corp., Armonk, NY, USA) before

starting the study. The randomization code is concealed using sequentially numbered sealed opaque envelopes. After the baseline assessment and obtaining written informed consent, a researcher who is not involved in the data analysis or clinical ratings opened the sealed envelope to identify the group to which the participant is allocated (RIC or sham-RIC group) and perform the treatment.

3.5 Sample size

We estimate that the PD on the affected sides at 2 days after IVT will be 30.12° in the sham-RIC group, according to our pre-experimental work. Here, we estimate a 9-degree improvement with RIC based on the results of preliminary observations. Using a two-tailed t-test to compare the difference between means and assuming α=0.05 and statistical power of 80%, the estimate sample size is 80 patients (40 each in the RIC and sham-RIC groups). Considering a 20% loss to follow up, we estimate that 100 patients will be required (50 each in the RIC and sham-RIC groups).

4. ANALYSIS POPULATIONS

The following analysis populations are planned for the study:

4.1 Intention-to-Treated (ITT) population

According to the basic principle of intention-to-treat (ITT), all randomly assigned patients will be included in the ITT population. The ITT population will be the main analysis population for the efficacy endpoint, and subjects will be analysed according to the treatment group to which they were assigned at randomization.

4.2 Per-protocol (PP) population

The PP population is a subset of ITT population, including all randomized subjects who have been treated in the study without major protocol deviations that may significantly impact the interpretation of efficacy results. Detailed protocol deviation criteria will be determined before database lock. This population will be used for the supportive analyses. Participants will be excluded from the ITT population if they:

1) Violate inclusion or exclusion criteria;

2) Two CA monitors are not completed;

3) Switched treatment (e.g. a participant is randomised to RIC group but received sham-RIC treatment).

Prior to database lock, the Steering Committee will develop and validate a list of patients to be removed from the randomized patients to create the PP population.

4.3 Safety population

The safety population includes all patients who received at least one RIC or sham-RIC treatment. If the randomization protocol is violated, patients will be classified based on the actual treatment they receive.

5. GENERAL ISSUES FOR STATISTICAL ANALYSES

5.1 Analysis software

All statistical analyses will be performed using SPSS® Software version 19 or higher.

5.2 Presentation of results

In terms of normally distributed continuous data, the statistics provided will include mean, and standard deviation (SD). If the continuous data are not normally distributed, median, lower quartile (Q1), and upper quartile (Q3) will be given, unless other specifications are stated. The summary of categorical data will be expressed in the number of patients and corresponding percentages.

5.3 Methods for withdrawals

If a subject withdraws from the study before their final scheduled follow-up visit, which is set for 90 days post-IVT, they will need to fill out a withdrawal visit section in the Case Report Form. The reason for withdrawing from the study will be recorded. Those participants who withdraw before their final follow-up will still be included in the analysis. However, those who do not receive any treatment after randomization will not be included in the end-point analysis.

5.4 Methods for missing data

All efforts will be made to minimize the amount of missing data, particularly the CA assessment.

5.4.1 Baseline covariates

Missing baseline covariates will be imputed using simple imputation methods in the covariate adjusted analysis based on the covariate distributions, should the missing values for a particular covariate be less than 5%. For a continuous variable, any missing values will be imputed with randomly selected values from a normal distribution, using the mean and standard deviation (SD) computed from the available sample. As for categorical variables, the missing values will be imputed with the multinomial distribution.

5.4.2 Efficacy outcomes

The missing values of the efficacy endpoint will be imputed with multiple permutations.

5.5 Further exploratory analyses

If deemed necessary, further exploratory analysis can be conducted, which will be decided by the Trial Management Group.

6. STATISTICAL ANALYSES

6.1 Demographics and baseline characteristics

Clinical important demography and baseline characteristics at screening will be statistically summarized by treatment group in both the ITT population and PP population. The variables to be summarized include but are not restricted to the following: age, sex, vascular risk factors, hematologic indices, pre-thrombolysis NIHSS score, systolic blood pressure, diastolic blood pressure, heart rate and TOAST subtypes.

Whether the distribution of data conforms to normality is determined by the Shapiro–Wilk test.

6.2 Primary efficacy analyses

All primary efficacy analyses will provide the point estimate of treatment effect along with the corresponding two-sided 95% confidence intervals (CIs). The primary analysis of the primary efficacy will be based on ITT population, which will be supported by the secondary analysis based

on PP population.

The primary endpoint is phase difference (PD, the main parameter of CA) on the affected side at 2 days after IVT.

6.2.1 Crude analysis

Main conclusion will be drawn from the ITT analysis of the primary endpoint. The null and alternative hypotheses to be tested appear below:

H0: $P_{\mathrm{RIC}} = P_{\mathrm{control}}$ versus H1: $P_{\mathrm{RIC}} \neq P_{\mathrm{control}}$

Where P_{RIC} and P_{control} are the RIC and control (sham-RIC) population PD on the affected side at 2 days after IVT of subjects.

The hypothesis of primary outcome will be tested by in the ITT population using a linear regression model to determine β and its 95% CI. In the linear regression model, the CA at 2 days after IVT will be treated as the response variable and the treatment as the only predictor.

6.2.2 Covariate adjusted analysis

Adjusted analyses will be carried out on the primary efficacy outcome to determine whether the treatment effect estimate is affected with the inclusion of covariates at baseline. The following covariates will be adjusted in the linear regression model:

a) Age;

b) Sex;

c) Pre-thrombolysis NIHSS score;

Adjusted β with 95% CI will be derived from the above model.

Imputation for baseline missing covariates (see description in the Section 5.4.1) will be made for covariate adjusted analysis.

6.3 Secondary efficacy outcomes analyses

Secondary efficacy outcomes include PD on the unaffected side at 2 days after IVT, gain on the affected side at 2 days after IVT, gain on the unaffected side at 2 days after IVT, PD on the affected side at 7 days after IVT, PD on the unaffected side at 7 days after IVT, gain on the affected side at 7 days after IVT, gain on the unaffected side at 7 days after IVT, NIHSS score at 24 h and 7 days after IVT, proportion of patients with favorable outcomes (mRS score≤ 2) at 90 days, final infarct volume, incidence of hemorrhagic transformation within 24 hours, serum levels of brain-injury biomarkers at 24 h after IVT.

All secondary efficacy analyses will provide the point estimate of treatment effect with associated two-sided 95% confidence intervals (CIs). Statistical analyses of the secondary efficacy endpoints will primarily be based on the ITT population. Secondarily, the same analyses will be repeated on the PP population.

6.3.1 Analysis of binary outcomes

Binary outcomes will be summarized by number (%) of participants with event by treatment group. The OR and its two-sided 95% CIs between RIC and sham-RIC group will be estimated though logistic regression model.

6.3.2 Analysis of continuous outcomes

PD, gain, NHISS score, final infarct volume, serum levels of brain-injury biomarkers will be treated as a continuous outcome. A GLM with normal distribution and identity link function will be used to compare the difference between the 2 groups. Log transformation may be performed if normality or variance homogeneity assumptions for residuals are violated after visual inspection of their histogram and scatterplots.

6.4 Safety analyses

All safety analyses will be conducted using the safety population only. Adverse events (AEs) or serious adverse events (SAEs) will be restricted to those occurring within 90 days.

Safety analyses will summarize the number (%) of any AEs and SAEs occurring within 90 days. The number of patients with any AEs and SAEs will be analyzed using $\chi 2$ or Fisher's Exact test to compare the difference between treatment groups.

7. Reference

1. Roth GA, Mensah GA, Johnson CO, et al. Global Burden of Cardiovascular Diseases and Risk Factors, 1990-2019: Update From the GBD 2019 Study. J Am Coll Cardiol. 2020;76(25):2982-3021. doi:10.1016/ j. jacc.2020.11.010.

2. Powers WJ, Rabinstein AA, Ackerson T, et al. Guidelines for the Early Management of Patients With Acute Ischemic Stroke: 2019 Update to the 2018 Guidelines for the Early Management of Acute Ischemic Stroke: A Guideline for Healthcare Professionals From the American Heart Association/American Stroke Association. Stroke. 2019;50(12): e344-e418. doi:10.1161/STR.0000000000000211.

3. Berge E, Whiteley W, Audebert H, et al. European Stroke Organisation (ESO) guidelines on intravenous thrombolysis for acute ischaemic stroke. Eur Stroke J. 2021;6(1): I-LXII. doi:10.1177/2396987321989865.

4. Chinese Society of Neurology; Chinese Stroke Society. Chinese Guideline for Endovascular Treatment of Acute Ischemic Stroke 2018. Chin J Neurol. 2018; 51:666-682. doi:10.3969/ j. issn.1673-5765.2018.07.014.

5. Hankey GJ. Stroke. Lancet. 2017;389(10069):641-654. doi:10.1016/S0140-6736(16)30962-X.

6. Emberson J, Lees KR, Lyden P, et al. Effect of treatment delay, age, and stroke severity on the effects of intravenous thrombolysis with alteplase for acute ischaemic stroke: a meta-analysis of individual patient data from randomised trials. Lancet. 2014;384(9958):1929-1935. doi:10.1016/S0140-6736(14)60584-5.

7. MacGregor DG, Carswell HV, Graham DI, et al. Impaired cerebral autoregulation 24 h after induction of transient unilateral focal ischaemia in the rat. Eur J Neurosci. 2000;12(1):58-66.

8. Fan JL, O'Donnell T, Lanford J, et al. Dietary nitrate reduces blood pressure and cerebral artery velocity fluctuations and improves cerebral autoregulation in transient ischemic attack patients. J Appl Physiol (1985). 2020;129(3):547-57.

9. Aaslid R LK, Sorteberg W, Nornes H. Cerebral autoregulation dynamics in humans. Stroke. 1989;20(1):45-52.

10. Murry CE, Jennings RB, Reimer KA. Preconditioning with ischemia: a delay of lethal cell injury in ischemic myocardium. Circulation. 1986;74(5):1124-1136. doi:10.1161/01. cir. 74.5.1124.

11. Weir P, Maguire R, O'Sullivan SE, England TJ. A meta-analysis of remote ischaemic conditioning in experimental stroke. J Cereb Blood Flow Metab. 2021;41(1):3-13. doi:

10.1177/0271678X20924077.
12. Guo ZN, Guo WT, Liu J, et al. Changes in cerebral autoregulation and blood biomarkers after remote ischemic preconditioning. Neurology. 2019;93(1): e8-e19.
13. Qu Y, Zhang P, He QY, et al. The Impact of Serial Remote Ischemic Conditioning on Dynamic Cerebral Autoregulation and Brain Injury Related Biomarkers. Front Physiol. 2022; 13:835173.
14. Hacke W, Kaste M, Fieschi C, et al. Intravenous thrombolysis with recombinant tissue plasminogen activator for acute hemispheric stroke. The European Cooperative Acute Stroke Study (ECASS). JAMA. 1995 Oct 4;274(13):1017-25.

Statistical Analysis Plan

Effect of Remote Ischemic Conditioning on Cerebral Hemodynamics in Patients After Intravenous Thrombolysis

(RICCH-IVT)

Version 1.0, 26 December 2022

Principal Investigator

Yi Yang, MD, PhD

The First Hospital of Jilin University, Jilin University, Changchun, China

ClinicalTrials.gov registration number: NCT05598658

Contents

LIST OF ABBREVIATIONS **72**

1. INTRODUCTION **73**

1.1 Background to this study 73

1.2 Purpose of the statistical analysis plan 73

2. STUDY OBJECTIVES AND OUTCOMES **73**

2.1 Study Objectives 74

2.2 Study Outcomes 74

2.2.1 Primary efficacy outcome 74

2.2.2 Secondary efficacy outcomes 74

2.2.3 Safety outcomes 74

2.2.4 Case definitions 74

3. STUDY METHODS **75**

3.1 Overall study design 75

3.2 Selection of study population 75

3.2.1 Inclusion criteria 75

3.2.2 Exclusion criteria 75

3.3 Treatments 75

3.4 Method of treatment assignment and randomization 75

3.5 Sample size 76

4. ANALYSIS POPULATION **76**

4.1 Intention-to-Treated (ITT) population 76

4.2 Per-protocol (PP) population 76

4.3 Safety population 76

5. GENERAL ISSUES FOR STATISTICAL ANALYSES **76**

5.1 Analysis software 76

5.2 Presentation of results 76

5.3 Methods for withdrawals 77

5.4 Methods for missing data 77

5.4.1 Baseline covariates 77

5.4.2 Efficacy outcomes 77

5.5 Further exploratory analyses 77

6. STATISTICAL ANALYSES **77**

6.1 Demographics and baseline characteristics..77
6.2 Primary efficacy analyses 77
6.2.1 Crude analysis 78
6.2.2 Covariate adjusted analysis 78
6.3 Secondary efficacy outcomes analyses 78
6.3.1 Analysis of binary outcomes 78
6.3.2 Analysis of continuous outcomes..79
6.4 Safety analyses..79
7. REFERENCE 79

LIST OF ABBREVIATIONS

Abbreviations	Definitions
AE	Adverse Event
AIS	Acute Ischemic Stroke
CI	Confidence Interval
FCS	Fully Conditional Method
ICH	Intracranial Hemorrhage
ITT	Intention-to-Treat
IVT	Intravenous Thrombolysis
MAR	Missing-at-Random
mRS	modified Rankin Scale
NIHSS	National Institute of Health Stroke Scale
NRS	Numerical Rating Scale
PP	Per-protocol
RIC	Remote Ischemic Conditioning
OR	Odds Ratio
SAE	Serious Adverse Event
SAP	Statistical Analysis Plan
SD	Standard Deviation
PD	Phase difference

1. INTRODUCTION

1.1 Background to this study

Stroke is a leading cause of global mortality and disability[1]. Intravenous thrombolysis (IVT) within 4.5 h of stroke onset is recognized as one of the most effective treatments for acute ischemic stroke (AIS)[2-4]. Even so, about 2/3 patients still do not benefit from alteplase intravenous thrombolysis[5-6]. Therefore, how to increase the benefit of IVT, improve the success rate of acute ischemic stroke patients and improve the quality of life of patients have become difficult problems for scientists in various countries.

Cerebral hemodynamics is closely related to the prognosis of ischemic stroke patients. As an important index of cerebral hemodynamics, cerebral autoregulation (CA) refers to a complex process in which intracranial arterioles maintain relatively stable cerebral blood by contraction or relaxation when systemic arterial blood pressure changes, causing changes in intracranial pressure, which can avoid hyperperfusion or hypoperfusion of brain tissue[7-9]. Our previous studies have shown that CA impairment is closely associated with 3-month prognosis in patients with ischemic stroke, and early CA decline is an independent risk factor for poor prognosis.

Remote ischemic conditioning (RIC) is a treatment method that gives intermittent non-lethal reversible ischemic stimulation to a remote vascular bed, organ or tissue to activate endogenous protective mechanisms to protect important ischemic organs[10]. The underlying neuroprotective mechanism triggered by RIC induces gene expression, alters pathways, promotes neurogenesis and blood vessel development, reduces oxidative stress and neuronal apoptosis, and inhibits proinflammatory signals in the ischemic brain[11]. In 2019, our team studied the effects of RIC on CA and blood biomarkers in healthy adults. The results showed that the ability of dynamic CA in healthy adults continued to increase within 6 hours to at least 24 hours after RIC[12]; In addition, at least 2 neuroprotective factors and 4 inflammation-related biological markers in blood changed rapidly within a short time after ischemia, thus providing new evidence for RIPC to induce neuroprotection and improve cerebral vascular function. Recently, our team investigated the effects of a series of RIC on dynamic CA and brain damage-related biomarkers, and showed that after four RIC sessions, the CA was significantly higher than baseline in healthy adults, after 7-day RIC, the CA increase lasts for at least 28 days[13]. At the same time, there were no differences in different serum biomarkers associated with brain damage before and after RIC. The study provides new support for the safety and efficacy of RIC.

However, there is still a lack of studies on the relationship between RIC and cerebral hemodynamics in patients with IVT. Based on the above background, our team designed this study to explore the effect of RIC on cerebral hemodynamics in patients with IVT.

1.2 Purpose of the statistical analysis plan

The purpose of this statistical analysis plan (SAP) is to outline the planned analyses to be completed to support the completion of the clinical study report. The planned analyses identified in this SAP will be included in regulatory submissions and/or future manuscripts. In addition, exploratory analyses not necessarily identified in this SAP may be conducted to support the clinical development plan. Any post-hoc, or unplanned, analyses outside of this SAP will be clearly identified in the respective clinical study report.

2. STUDY OBJECTIVES AND OUTCOMES

2.1 Study objectives

The primary objectives of the study is to determine the effect of RIC on cerebral hemodynamics in patients after IVT.

2.2 Study outcomes

2.2.1 Primary efficacy outcome

The primary endpoint is phase difference (PD, the main parameter of CA) on the affected side at 1-2 days after IVT.

2.2.2 Secondary efficacy outcomes

The secondary efficacy outcomes for this study are as follows:

1) PD on the unaffected side at 1-2 days after IVT.
2) Gain (the parameter of CA) on the affected side at 1-2 days after IVT.
3) Gain on the unaffected side at 1-2 days after IVT.
4) PD on the affected side at 7-10 days after IVT.
5) PD on the unaffected side at 7-10 days after IVT.
6) Gain on the affected side at 7-10 days after IVT.
7) Gain on the unaffected side at 7-10 days after IVT.
8) NIHSS score at 24 h and 7 days after IVT.
9) Proportion of patients with favorable outcomes (mRS score $\leqslant$ 2) at 90 days.
10) Final infarct volume.
11) Incidence of hemorrhagic transformation within 24 hours.
12) Serum levels of brain-injury biomarkers at 24 h after IVT.

2.2.3 Safety outcomes

1) Mortality within 90 days.
2) All adverse events within 90 days.

2.2.4 Case definitions

1) Hemorrhagic transformation:

Hemorrhagic transformation is determined based on the computer tomography scan conducted 24 hours after IVT, using the European Cooperative Acute Stroke Study (ECASS) criteria[14].

2) Death:

All deaths during the study period will be recorded. The cause of each death will be determined based on clinical assessments carried out by the physicians involved in the study.

3) CA assessments:

Before the examination, all patients are instructed to relax in the supine position for 10 min in a dedicated quiet examination room with a controlled temperature ranging from 20°C to 24°C. The bilateral cerebral blood flow velocity (CBFV) in the middle cerebral artery is measured using transcranial Doppler (MultiDop X4; DWL, Sipplingen, Germany) sonography. Two 2-MHz probes are fixed with a customized head frame in the bilateral temporal bone window at a depth of 45–60 mm. Simultaneously, arterial blood pressure in the digital artery (measured using a servo-controlled plethysmograph [Finometer Model 1; FMS, Amsterdam, the Netherlands]) is continuously recorded. The end-tidal CO2 is measured using a capnograph (MultiDop X4; DWL) with a nasal cannula. The

real-time recordings last 10 min and are stored for further analysis.

3. STUDY METHODS

3.1 Overall study design

The RICCH-IVT study is a single-center, single-blinded, endpoint-blinded, randomized controlled clinical trial in patients with AIS who undergo IVT within 4.5 hours of onset. Patients will be randomized 1:1 to either RIC group or control (sham-RIC) group. It is planned to recruit 100 patients from the First Hospital of Jilin University.

3.2 Selection of study population

3.2.1 Inclusion criteria

1) Age ≥ 18 and < 80 years, both sexes.
2) A clear clinical diagnosis of acute ischemic stroke and treatment with standard rt-PA (0.9 mg/kg) IVT within 4.5 h of stroke onset.
3) Pre-onset modified Rankin Scale (mRS) score ≤1.
4) Baseline National Institute of Health Stroke Scale (NIHSS) score ≥5 and ≤25.
5) Glasgow Coma Scale score ≥8.

3.2.2 Exclusion Criteria

1) Having received bridging therapy (IVT plus mechanical thrombectomy).
2) Previous history of atrial fibrillation or electrocardiographic evidence of atrial fibrillation.
3) Contraindications to RIC treatment or previous RIC treatment or similar treatment.
4) Pregnancy or breastfeeding.
5) Life expectancy of ≤3 months or inability to complete the study for other reasons.
6) Unwillingness to be followed up or poor treatment compliance or participation in other clinical studies.
7) Had insufficient bilateral temporal bone windows for insonation of the middle cerebral artery.

3.3 Treatments

The two trial arms of this study are as follows:
RIC group: RIC, which is given at 6 and 18–24 h after IVT.
Control (sham-RIC) group: sham-RIC, which is given at 6 and 18–24 h after IVT.

Patients in the two groups will receive standard medical care. After randomization, participants in both groups will undergo RIC treatment at 6 h and 18-24 h after IVT using an automated device (BB-RIC-D5/LAPUL Medical Devices Co., Ltd, Beijing, China) placed on the upper arm of the unaffected side. Each RIC will consist of four cycles of arm ischemia with cuffs inflated to a pressure of 200 mmHg (RIC group) or 60 mmHg (control group) for 5 min, followed by reperfusion for another 5 min, for a total procedure time of 40 min. Hospital-based nurses will perform the RIC procedure.

3.4 Method of treatment assignment and randomization

Randomization is established by a researcher who is not involved in the recruitment and assessment of the participants. A random numerical sequence with a 1:1 allocation ratio is computer-generated by an independent biostatistician using SPSS (IBM Corp., Armonk, NY, USA) before

starting the study. The randomization code is concealed using sequentially numbered sealed opaque envelopes. After the baseline assessment and obtaining written informed consent, a researcher who is not involved in the data analysis or clinical ratings opened the sealed envelope to identify the group to which the participant is allocated (RIC or sham-RIC group) and perform the treatment.

3.5 Sample size

We estimate that the PD on the affected sides at 1-2 days after IVT will be 30.12° in the sham-RIC group, according to our pre-experimental work. Here, we estimate a 9-degree improvement with RIC based on the results of preliminary observations. Using a two-tailed t-test to compare the difference between means and assuming $\alpha=0.05$ and statistical power of 80%, the estimate sample size is 80 patients (40 each in the RIC and sham-RIC groups). Considering a 20% loss to follow up, we estimate that 100 patients will be required (50 each in the RIC and sham-RIC groups).

4. ANALYSIS POPULATIONS

The following analysis populations are planned for the study:

4.1 Intention-to-Treated (ITT) population

According to the basic principle of intention-to-treat (ITT), all randomly assigned patients will be included in the ITT population. The ITT population will be the main analysis population for the efficacy endpoint, and subjects will be analysed according to the treatment group to which they were assigned at randomization.

4.2 Per-protocol (PP) population

The PP population is a subset of ITT population, including all randomized subjects who have been treated in the study without major protocol deviations that may significantly impact the interpretation of efficacy results. Detailed protocol deviation criteria will be determined before database lock. This population will be used for the supportive analyses. Participants will be excluded from the ITT population if they:

1) Violate inclusion or exclusion criteria;

2) Two CA monitors are not completed;

3) Switched treatment (e.g. a participant is randomised to RIC group but received sham-RIC treatment).

Prior to database lock, the Steering Committee will develop and validate a list of patients to be removed from the randomized patients to create the PP population.

4.3 Safety population

The safety population includes all patients who received at least one RIC or sham-RIC treatment. If the randomization protocol is violated, patients will be classified based on the actual treatment they receive.

5. GENERAL ISSUES FOR STATISTICAL ANALYSES

5.1 Analysis software

All statistical analyses will be performed using SPSS® Software version 19 or higher.

5.2 Presentation of results

In terms of normally distributed continuous data, the statistics provided will include mean and standard deviation (SD). If the continuous data are not normally distributed, median, lower quartile (Q1), and upper quartile (Q3) will be given, unless other specifications are stated. The summary of categorical data will be expressed in the number of patients and corresponding percentages.

5.3 Methods for withdrawals

If a subject withdraws from the study before their final scheduled follow-up visit, which is set for 90 days post-IVT, they will need to fill out a withdrawal visit section in the Case Report Form. The reason for withdrawing from the study will be recorded. Those participants who withdraw before their final follow-up will still be included in the analysis. However, those who do not receive any treatment after randomization will not be included in the end-point analysis.

5.4 Methods for missing data

All efforts will be made to minimize the amount of missing data, particularly the CA assessment.

5.4.1 Baseline covariates

Missing baseline covariates will be imputed using simple imputation methods in the covariate adjusted analysis based on the covariate distributions, should the missing values for a particular covariate be less than 5%. For a continuous variable, any missing values will be imputed with randomly selected values from a normal distribution, using the mean and standard deviation (SD) computed from the available sample. As for categorical variables, the missing values will be imputed with the multinomial distribution.

5.4.2 Efficacy outcomes

The missing values of the efficacy endpoint will be imputed with multiple permutations.

5.5 Further exploratory analyses

If deemed necessary, further exploratory analysis can be conducted, which will be decided by the Trial Management Group.

6. STATISTICAL ANALYSES

6.1 Demographics and baseline characteristics

Clinical important demography and baseline characteristics at screening will be statistically summarized by treatment group in both the ITT population and PP population. The variables to be summarized include but are not restricted to the following: age, sex, vascular risk factors, hematologic indices, pre-thrombolysis NIHSS score, systolic blood pressure, diastolic blood pressure, heart rate and TOAST subtypes.

Whether the distribution of data conforms to normality is determined by the Shapiro–Wilk test.

6.2 Primary efficacy analyses

All primary efficacy analyses will provide the point estimate of treatment effect along with the corresponding two-sided 95% confidence intervals (CIs). The primary analysis of the primary efficacy will be based on ITT population, which will be supported by the secondary analysis based

on PP population.

The primary endpoint is phase difference (PD, the main parameter of CA) on the affected side at 1-2 days after IVT.

6.2.1 Crude analysis

Main conclusion will be drawn from the ITT analysis of the primary endpoint. The null and alternative hypotheses to be tested appear below:

H0: $P_{\mathrm{RIC}} = P_{\mathrm{control}}$ versus H1: $P_{\mathrm{RIC}} \neq P_{\mathrm{control}}$

Where P_{RIC} and P_{control} are the RIC and control (sham-RIC) population PD on the affected side at 1-2 days after IVT of subjects.

The hypothesis of primary outcome will be tested by in the ITT population using a linear regression model to determine β and its 95% CI. In the linear regression model, the PD on the affected side at 1-2 days after IVT will be treated as the response variable and the treatment as the only predictor.

6.2.2 Covariate adjusted analysis

Adjusted analyses will be carried out on the primary efficacy outcome to determine whether the treatment effect estimate is affected with the inclusion of covariates at baseline. The following covariates will be adjusted in the linear regression model:

a) Age;

b) Sex;

c) Pre-thrombolysis NIHSS score;

Adjusted β with 95% CI will be derived from the above model.

Imputation for baseline missing covariates (see description in the Section 5.4.1) will be made for covariate adjusted analysis.

6.3 Secondary efficacy outcomes analyses

Secondary efficacy outcomes include PD on the unaffected side at 1-2 days after IVT, gain on the affected side at 1-2 days after IVT, gain on the unaffected side at 1-2 days after IVT, PD on the affected side at 7-10 days after IVT, PD on the unaffected side at 7-10 days after IVT, gain on the affected side at 7-10 days after IVT, gain on the unaffected side at 7-10 days after IVT, NIHSS score at 24 h and 7 days after IVT, proportion of patients with favorable outcomes (mRS score≤ 2) at 90 days, final infarct volume, incidence of hemorrhagic transformation within 24 hours, serum levels of brain-injury biomarkers at 24 h after IVT.

All secondary efficacy analyses will provide the point estimate of treatment effect with associated two-sided 95% confidence intervals (CIs). Statistical analyses of the secondary efficacy endpoints will primarily be based on the ITT population. Secondarily, the same analyses will be repeated on the PP population.

6.3.1 Analysis of binary outcomes

Binary outcomes will be summarized by number (%) of participants with event by treatment group. The OR and its two-sided 95% CIs between RIC and sham-RIC group will be estimated though logistic regression model.

6.3.2 Analysis of continuous outcomes

PD, gain, NHISS score, final infarct volume, serum levels of brain-injury biomarkers will be treated as a continuous outcome. A GLM with normal distribution and identity link function will be used to compare the difference between the 2 groups. Log transformation may be performed if normality or variance homogeneity assumptions for residuals are violated after visual inspection of their histogram and scatterplots.

6.4 Safety analyses

All safety analyses will be conducted using the safety population only. Adverse events (AEs) or serious adverse events (SAEs) will be restricted to those occurring within 90 days.

Safety analyses will summarize the number (%) of any AEs and SAEs occurring within 90 days. The number of patients with any AEs and SAEs will be analyzed using $\chi 2$ or Fisher's Exact test to compare the difference between treatment groups.

7. Reference

1. Roth GA, Mensah GA, Johnson CO, et al. Global Burden of Cardiovascular Diseases and Risk Factors, 1990-2019: Update From the GBD 2019 Study. J Am Coll Cardiol. 2020;76(25):2982-3021. doi:10.1016/ j. jacc.2020.11.010.
2. Powers WJ, Rabinstein AA, Ackerson T, et al. Guidelines for the Early Management of Patients With Acute Ischemic Stroke: 2019 Update to the 2018 Guidelines for the Early Management of Acute Ischemic Stroke: A Guideline for Healthcare Professionals From the American Heart Association/American Stroke Association. Stroke. 2019;50(12): e344-e418. doi:10.1161/STR.0000000000000211.
3. Berge E, Whiteley W, Audebert H, et al. European Stroke Organisation (ESO) guidelines on intravenous thrombolysis for acute ischaemic stroke. Eur Stroke J. 2021;6(1): I-LXII. doi:10.1177/2396987321989865.
4. Chinese Society of Neurology; Chinese Stroke Society. Chinese Guideline for Endovascular Treatment of Acute Ischemic Stroke 2018. Chin J Neurol. 2018; 51:666-682. doi:10.3969/ j. issn.1673-5765.2018.07.014.
5. Hankey GJ. Stroke. Lancet. 2017;389(10069):641-654. doi:10.1016/S0140-6736(16)30962-X.
6. Emberson J, Lees KR, Lyden P, et al. Effect of treatment delay, age, and stroke severity on the effects of intravenous thrombolysis with alteplase for acute ischaemic stroke: a meta-analysis of individual patient data from randomised trials. Lancet. 2014;384(9958):1929-1935. doi:10.1016/S0140-6736(14)60584-5.
7. MacGregor DG, Carswell HV, Graham DI, et al. Impaired cerebral autoregulation 24 h after induction of transient unilateral focal ischaemia in the rat. Eur J Neurosci. 2000;12(1):58-66.
8. Fan JL, O'Donnell T, Lanford J, et al. Dietary nitrate reduces blood pressure and cerebral artery velocity fluctuations and improves cerebral autoregulation in transient ischemic attack patients. J Appl Physiol (1985). 2020;129(3):547-57.
9. Aaslid R LK, Sorteberg W, Nornes H. Cerebral autoregulation dynamics in humans. Stroke. 1989;20(1):45-52.
10. Murry CE, Jennings RB, Reimer KA. Preconditioning with ischemia: a delay of lethal cell injury in ischemic myocardium. Circulation. 1986;74(5):1124-1136. doi:10.1161/01. cir. 74.5.1124.
11. Weir P, Maguire R, O'Sullivan SE, England TJ. A meta-analysis of remote ischaemic

conditioning in experimental stroke. J Cereb Blood Flow Metab. 2021;41(1):3-13. doi: 10.1177/0271678X20924077.
12. Guo ZN, Guo WT, Liu J, et al. Changes in cerebral autoregulation and blood biomarkers after remote ischemic preconditioning. Neurology. 2019;93(1): e8-e19.
13. Qu Y, Zhang P, He QY, et al. The Impact of Serial Remote Ischemic Conditioning on Dynamic Cerebral Autoregulation and Brain Injury Related Biomarkers. Front Physiol. 2022; 13:835173.
14. Hacke W, Kaste M, Fieschi C, et al. Intravenous thrombolysis with recombinant tissue plasminogen activator for acute hemispheric stroke. The European Cooperative Acute Stroke Study (ECASS). JAMA. 1995 Oct 4;274(13):1017-25.

Details of Statistical Analysis Plan Change

Section	Version 1.0	Version 2.0
**Protocol version and date**	Version 1.0 of December 26, 2022	Version 2.0 of March 05, 2023
**SAP Version**	1.0	2.0
**Version Date**	26 December 2022	05 March 2023
**2.2 Study outcomes**	**2.2.1 Primary efficacy outcome**<br>The primary endpoint is phase difference (PD, the main parameter of CA) on the affected side at 1-2 days after IVT.<br><br>**2.2.2 Secondary efficacy outcomes**<br>The secondary efficacy outcomes for this study are as follows:<br>1) PD on the unaffected side at1- 2 days after IVT.<br>2) Gain (the parameter of CA) on the affected side at 1-2 days after IVT.<br>3) Gain on the unaffected side at 1-2 days after IVT.<br>4) PD on the affected side at 7-10 days after IVT.<br>5) PD on the unaffected side at 7-10 days after IVT.<br>6) Gain on the affected side at 7-10 days after IVT.<br>7) Gain on the unaffected side at 7-10 days after IVT.<br>8) NIHSS score at 24 h and 7 days after IVT.<br>9) Proportion of patients with favorable outcomes (mRS score⩽ 2) at 90 days.<br>10) Final infarct volume.<br>11) Incidence of hemorrhagic transformation within 24 hours.<br>12) Serum levels of brain-injury biomarkers at 24 h after IVT.	**2.2.1 Primary efficacy outcome**<br>The primary endpoint is phase difference (PD, the main parameter of CA) on the affected side at 2 days after IVT.<br><br>**2.2.2 Secondary efficacy outcomes**<br>The secondary efficacy outcomes for this study are as follows:<br>1) PD on the unaffected side at 2 days after IVT.<br>2) Gain (the parameter of CA) on the affected side at 2 days after IVT.<br>3) Gain on the unaffected side at 2 days after IVT.<br>4) PD on the affected side at 7 days after IVT.<br>5) PD on the unaffected side at 7 days after IVT.<br>6) Gain on the affected side at 7 days after IVT.<br>7) Gain on the unaffected side at 7 days after IVT.<br>8) NIHSS score at 24 h and 7 days after IVT.<br>9) Proportion of patients with favorable outcomes (mRS score⩽ 2) at 90 days.<br>10) Final infarct volume.<br>11) Incidence of hemorrhagic transformation within 24 hours.<br>12) Serum levels of brain-injury biomarkers at 24 h after IVT.

	**2.2.3 Safety outcomes**<br>1) Mortality within 90 days.<br>2) All adverse events within 90 days.	**2.2.3 Safety outcomes**<br>1) Mortality within 90 days.<br>2) All adverse events within 90 days.
**3.5 Sample size**	We estimate that the PD on the affected sides at 1-2 days after IVT will be 30.12° in the sham-RIC group, according to our pre-experimental work. Here, we estimate a 9-degree improvement with RIC based on the results of preliminary observations. Using a two-tailed t-test to compare the difference between means and assuming α=0.05 and statistical power of 80%, the estimate sample size is 80 patients (40 each in the RIC and sham-RIC groups). Considering a 20% loss to follow up, we estimate that 100 patients will be required (50 each in the RIC and sham-RIC groups).	We estimate that the PD on the affected sides at 2 days after IVT will be 30.12° in the sham-RIC group, according to our pre-experimental work. Here, we estimate a 9-degree improvement with RIC based on the results of preliminary observations. Using a two-tailed t-test to compare the difference between means and assuming α=0.05 and statistical power of 80%, the estimate sample size is 80 patients (40 each in the RIC and sham-RIC groups). Considering a 20% loss to follow up, we estimate that 100 patients will be required (50 each in the RIC and sham-RIC groups).
**6.2 Primary efficacy analyses**	All primary efficacy analyses will provide the point estimate of treatment effect along with the corresponding two-sided 95% confidence intervals (CIs). The primary analysis of the primary efficacy will be based on ITT population, which will be supported by the secondary analysis based on PP population.<br>The primary endpoint is phase difference (PD, the main parameter of CA) on the affected side at 1-2 days after IVT.	All primary efficacy analyses will provide the point estimate of treatment effect along with the corresponding two-sided 95% confidence intervals (CIs). The primary analysis of the primary efficacy will be based on ITT population, which will be supported by the secondary analysis based on PP population.<br>The primary endpoint is phase difference (PD, the main parameter of CA) on the affected side at 2 days after IVT.
**6.2.1 Crude analysis**	Main conclusion will be drawn from the ITT analysis of the primary endpoint. The null and alternative hypotheses to be tested appear below:<br>H0: $P_{\mathrm{RIC}} = P_{\mathrm{control}}$ versus H1: $P_{\mathrm{RIC}} \neq P_{\mathrm{control}}$<br>Where P_{RIC} and P_{control} are the RIC and control (sham-RIC)	Main conclusion will be drawn from the ITT analysis of the primary endpoint. The null and alternative hypotheses to be tested appear below:<br>H0: $P_{\mathrm{RIC}} = P_{\mathrm{control}}$ versus H1: $P_{\mathrm{RIC}} \neq P_{\mathrm{control}}$<br>Where P_{RIC} and P_{control} are the RIC and control (sham-RIC)

	population PD on the affected side at 1-2 days after IVT of subjects.<br>The hypothesis of primary outcome will be tested by in the ITT population using a linear regression model to determine β and its 95% CI. In the linear regression model, the CA at 1-2 days after IVT will be treated as the response variable and the treatment as the only predictor.	population PD on the affected side at 2 days after IVT of subjects.<br>The hypothesis of primary outcome will be tested by in the ITT population using a linear regression model to determine β and its 95% CI. In the linear regression model, the CA at 2 days after IVT will be treated as the response variable and the treatment as the only predictor.
**6.3 Secondary efficacy outcomes analyses**	Secondary efficacy outcomes include PD on the unaffected side at 1-2 days after IVT, gain on the affected side at 1-2 days after IVT, gain on the unaffected side at 1-2 days after IVT, PD on the affected side at 7-10 days after IVT, PD on the unaffected side at 7-10 days after IVT, gain on the affected side at 7-10 days after IVT, gain on the unaffected side at 7-10 days after IVT, NIHSS score at 24 h and 7 days after IVT, proportion of patients with favorable outcomes (mRS score≤ 2) at 90 days, final infarct volume, incidence of hemorrhagic transformation within 24 hours, serum levels of brain-injury biomarkers at 24 h after IVT.<br>All secondary efficacy analyses will provide the point estimate of treatment effect with associated two-sided 95% confidence intervals (CIs). Statistical analyses of the secondary efficacy endpoints will primarily be based on the ITT population. Secondarily, the same analyses will be repeated on the PP population.	Secondary efficacy outcomes include PD on the unaffected side at 2 days after IVT, gain on the affected side at 2 days after IVT, gain on the unaffected side at 2 days after IVT, PD on the affected side at 7 days after IVT, PD on the unaffected side at 7 days after IVT, gain on the affected side at 7 days after IVT, gain on the unaffected side at 7 days after IVT, NIHSS score at 24 h and 7 days after IVT, proportion of patients with favorable outcomes (mRS score≤ 2) at 90 days, final infarct volume, incidence of hemorrhagic transformation within 24 hours, serum levels of brain-injury biomarkers at 24 h after IVT.<br>All secondary efficacy analyses will provide the point estimate of treatment effect with associated two-sided 95% confidence intervals (CIs). Statistical analyses of the secondary efficacy endpoints will primarily be based on the ITT population. Secondarily, the same analyses will be repeated on the PP population.
